# Structural osteoarthritis pathogenesis correlates with distinct pain and dysfunction profiles after ACL injury in rats

**DOI:** 10.1038/s41684-026-01762-1

**Published:** 2026-07-01

**Authors:** Nicholas M. Pancheri, Nisha Kyathsandra, Jake Heinonen, Emily Sverdrup, Sanique M. South, Sruthi Ranganathan, Kaitlyn A. Link, Angela SP Lin, Nick J. Willett, Robert E. Guldberg

**Affiliations:** 1 Phil and Penny Knight Campus for Accelerating Scientific Impact, University of Oregon, Eugene, OR USA; 2 Oregon Health & Science University, Portland, OR USA; 3 The Veterans Affairs Portland Health Care System, Portland, OR USA

**Keywords:** Preclinical research, Cartilage, Bone, Osteoarthritis

## Abstract

Post-traumatic osteoarthritis (PTOA) frequently arises following knee injury, such as anterior cruciate ligament (ACL) rupture. Small animal models are critical for studying PTOA pathology and translating therapeutic interventions. Here our objective was to identify key structural PTOA pathologies and evaluate their associations with aberrant pain and function after a clinically relevant ACL injury in a small animal model. This work leveraged a suite of quantitative cartilage and bone analysis techniques using high-resolution contrast-enhanced microcomputed tomography to investigate the associations between pain-related behaviors and structural osteoarthritis pathogenesis throughout the knee joint in rats. We hypothesized that ACL rupture would increase pain sensitization and limb dysfunction and that this would correlate with the development of clinically relevant osteophytes and cartilage lesions at moderate (4 weeks) and severe disease stages (8 weeks). We showed that ACL rupture induced hyperalgesia and prolonged hindlimb weight-bearing dysfunction; these pain-related behaviors were strongly correlated with changes in the tibiofemoral subchondral bone plate, but not with osteophyte formation. Moreover, we show that patella bone pathology correlates strongly with pain sensitivity and limb function. Joint degeneration initially manifested in the posterior aspect of the medial tibiofemoral compartments, as indicated by full-thickness femoral cartilage lesions and tibial osteophytes at 4 weeks and corresponding tibial cartilage hypertrophy and subchondral sclerosis by 8 weeks. The established outcome parameters and association of structural pathogenesis with pain and dysfunction provide a foundation for studying fundamental PTOA disease etiology and testing therapeutic efficacy in a rodent preclinical model.

## Abstract (web summary)

Anterior cruciate ligament rupture in rat induces pain sensitization and limb dysfunction, correlating with tibiofemoral subchondral and patellar bone changes. These findings provide new insights into post-traumatic osteoarthritis disease etiology.

## Main

Osteoarthritis (OA) is the most common degenerative joint disease, affecting 300–500 million people worldwide, and there are currently no US Food and Drug Administration-approved treatments that can prevent, modify or reverse disease progression^1,2^. OA-associated impairment and pain contribute to reduced wages, unplanned early retirement and even a younger age of death^3–6^. This is particularly burdensome for patients with post-traumatic OA (PTOA), a subtype of OA diagnosed following joint injury or trauma, predominantly in the knee^5^. These patients go on to rapidly experience the same debilitating symptoms as primary OA but much earlier in life^6^. Unfortunately, there are no curative treatments for primary OA or PTOA, and it remains unclear which joint tissues should be targeted as primary mediators of pathogenesis, pain and function.

The anterior cruciate ligament (ACL) is a critical structure that maintains knee joint stability and function. ACL tears are one of the most common traumatic knee injuries; ACL injury increases the risk of developing PTOA by four- to tenfold^7,8^, with 21–50% of patients going on to experience PTOA symptoms^9–11^. This burden is exacerbated by knowledge gaps regarding how PTOA pathologies manifest after injury and how or whether these pathologies drive aberrant pain and function. Small animal models are commonly used for preclinical therapeutic testing before translation into larger animal models and eventually clinical populations. Rats are an attractive model organism for PTOA research because their size and joint architecture support the use of high-resolution imaging techniques to quantify tissue and cellular level changes and are generally more amenable to behavioral, locomotor and pain-related analyses compared with mice^12^. Surgical or chemical induction of PTOA in rats is the primary approach for rapidly producing consistent arthritic joint pathology, and the pathological and behavioral changes have been rigorously characterized by our group and others^13–16^. However, these induction models do not represent initiation of PTOA in human clinical populations and therefore introduce confounding factors when trying to link OA structural pathology to pain and function.

Multiple techniques exist to non-invasively induce a preclinical knee injury, such as an ACL rupture (ACLr), as an initiating stimulus that drives arthritic joint pathology in animal models. These modalities have been employed in mice to screen drugs, study the effects of exercise and investigate mechanistic insights (for example, genetic manipulations), but comparatively little work has leveraged this in rat models. A handful of studies together report on rat non-invasive ACL injury models, with early attempts^17^ to integrate contrast-enhanced microcomputed tomography (µCT)^18–20^, measures of pain sensitization^21^ and disease biomarkers at various time points^22^. However, none of these parameters was comprehensively assessed together in a single study, and there are no prior high-dimensional analyses for structural, biomarker and pain data taken together. These foundational works have begun to define the OA progression timeline after ACL injury and serve as justification for our comprehensive analysis at a moderate (4 weeks) and severe (8 weeks) disease stage.

**Fig. 1:**
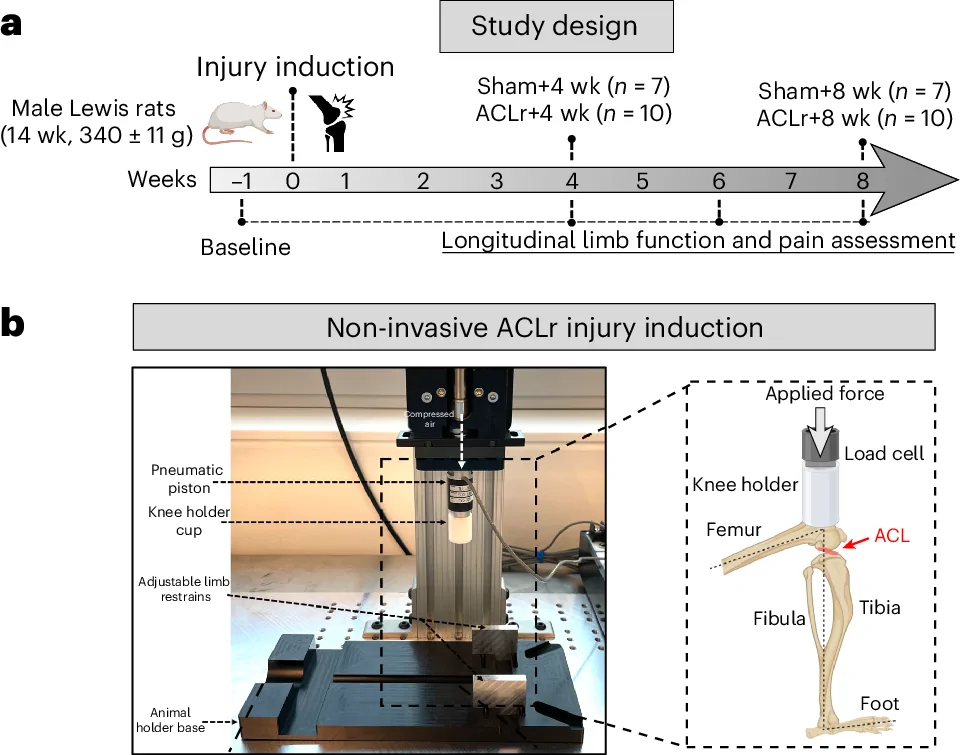
Study design and non-invasive injury modality. **a**, Skeletally mature male Lewis rats (*N* = 27) received a non-invasive knee injury to induce ACLr (*n* = 10 per group) or a sham injury (*n* = 7 per group), and structure changes were assessed after 4 or 8 weeks via contrast-enhanced µCT. Pain-related behavioral functions (von Frey and limb weight bearing) were longitudinally assessed at 4, 6 and 8 weeks, with baseline measurements recorded immediately before surgery. **b**, A non-invasive ACLr was delivered via a custom platform to deliver a single uniaxially load to the fixed hindlimb of each animal (loading rate 5 N s^−1^). wk, week. Figure created in BioRender; Lab, G. https://biorender.com/datlqb5 (2026).

The objectives of this work were to (1) characterize tissue pathology in multiple joint tissue compartments (tibia, femur and patella) and pain-related behavioral changes at two PTOA stages after non-invasive ACLr in rats and (2) identify salient OA disease features at multiple disease stages, and how these features relate to aberrant pain and function. We selected 4 and 8 weeks after ACLr to represent early-moderate and moderate-severe PTOA, respectively (Fig. 1). These time points represent critical time frames for preclinical research, including studying disease etiology, investigating physical rehabilitation regimens and testing of both prophylactic and regenerative disease-modifying OA drugs. We hypothesized that ACLr would increase hindlimb pain sensitization and limb function and that this would correlate with the development of clinically relevant osteophytes and cartilage lesions as markers of disease severity at both moderate (4 weeks) and severe (8 weeks) disease stages. Outcomes of this work benefit OA and sports injury research by identifying key pathological and behavioral markers of PTOA after a relevant injury, informing novel therapeutic development and testing, and supporting the fundamental study of OA etiopathogenesis.

## Results

### Spontaneous and evoked pain-related behaviors

#### Von Frey

**Fig. 2:**
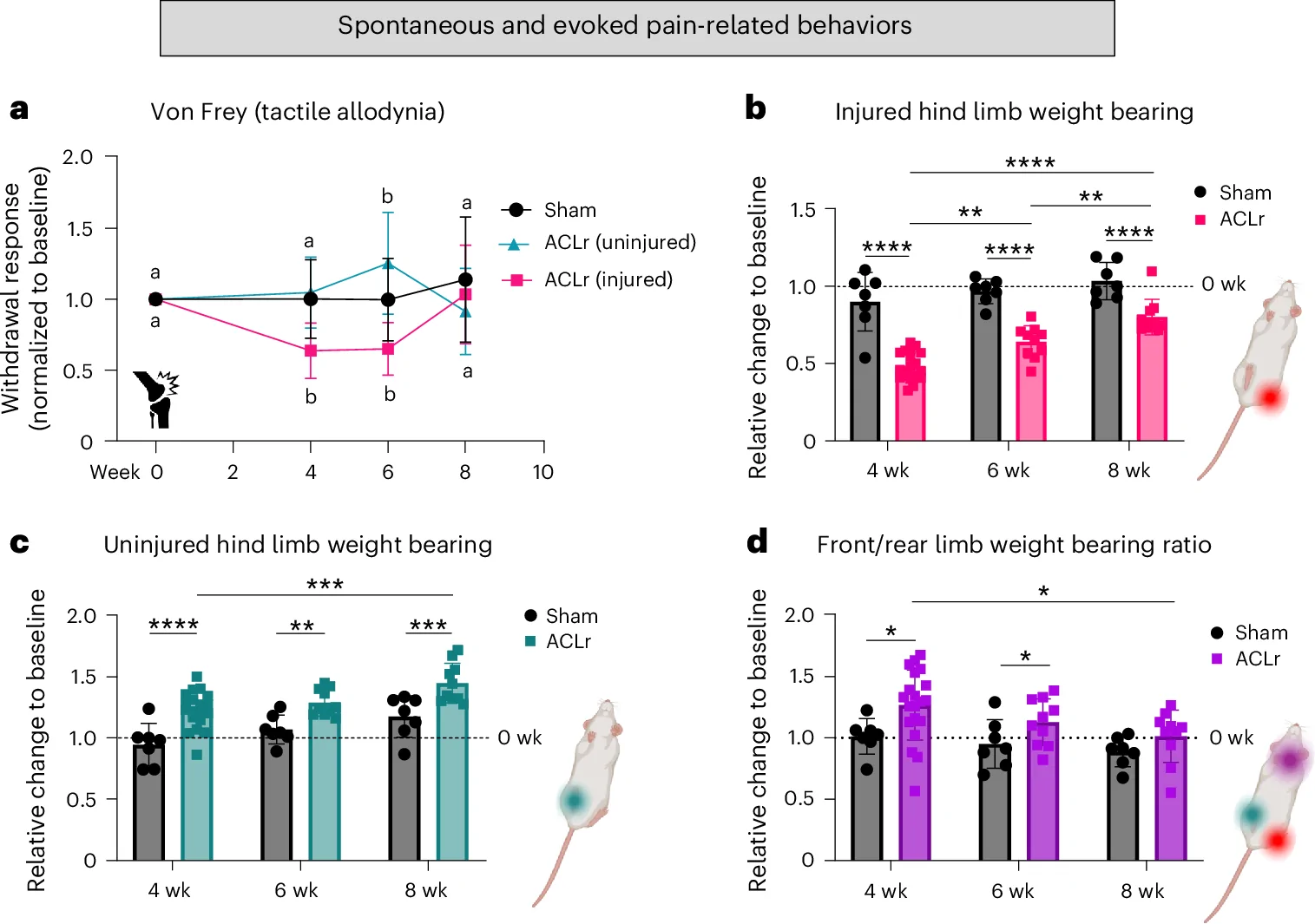
Spontaneous and evoked pain-related behaviors. Spontaneous and evoked pain-related behaviors were assessed before injury, and then at 4, 6 and 8 weeks (sham (left limb), *n* = 7; ACLr, *n* = 20 at 4 weeks and *n* = 10 at 8 weeks). **a**, Animals exhibited increased withdrawal responses indicative of hyperallodynia at 4 and 6 weeks after ACLr, which resolved to baseline levels by 8 weeks. Different letters (for example, a versus b) denote statistically significant differences between groups. **b**,**c**, Significantly reduced hindlimb weight bearing was recorded at all time points after injury (**b**), which corresponded to contralateral limb offloading (**c**). **d**, Compensatory shifts in the ratio of front-to-rear limb weight-bearing behavior were significant at 4 and 6 weeks but returned to sham and baseline levels by 8 weeks. In **a**–**d**, data are presented as mean ± standard deviation. Mixed-effects model with Tukey’s post-hoc test (*P* < 0.05). *P = 0.05, **P = 0.005, ***P = 0.0005, ****P = 0.00005. wk, weeks. Figure created in BioRender; Lab, G. https://biorender.com/datlqb5 (2026).

ACLr significantly decreased the paw withdrawal threshold in injured (ipsilateral) limbs 4 and 6 weeks post-injury compared with baseline (*P* < 0.001, *P* < 0.001) and sham measurements (*P* = 0.029, *P* < 0.001), indicating increased pain sensitization (Fig. 2a). Injured ACLr limbs recovered to baseline and sham levels after 8 weeks. Sham treatment did not significantly affect behavioral measures. Injured ACLr limbs had significantly reduced withdrawal responses compared with uninjured (contralateral) limbs at 4 and 6 weeks (*P* < 0.001, *P* = 0.002) (Fig. 2a).

#### Weight-bearing behavior

ACLr significantly reduced weight bearing on injured limbs at 4, 6 and 8 weeks compared with sham (*P* < 0.001, *P* < 0.001, *P* < 0.001) and baseline levels (*P* < 0.001, *P* < 0.001, *P* < 0.001) (Fig. 2b). Weight bearing on the ACLr injured limb significantly increased at 8 weeks compared with injured limb weight bearing at 4 and 6 weeks (*P* < 0.001, *P* = 0.001) (Fig. 2b). Uninjured contralateral limbs exhibited correspondingly increased weight bearing at 4, 6 and 8 weeks compared with sham (*P* < 0.001, *P* = 0.006, *P* < 0.001) and remained elevated compared with baseline (*P* < 0.001, *P* < 0.001, *P* < 0.001) through the duration of the study (Fig. 2c). The ratio of weight bearing on the rear hindlimbs together relative to weight bearing on the forelimbs initially increased at 4 weeks compared with baseline (*P* < 0.001) but decreased at 8 weeks, and it was significantly increased at 4 and 6 weeks compared with sham (*P* = 0.012, *P* = 0.005) (Fig. 2d). The sham procedure did not significantly affect weight bearing.

### Representative structural pathologies (2D tomograms, histology)

#### Femur

**Fig. 3:**
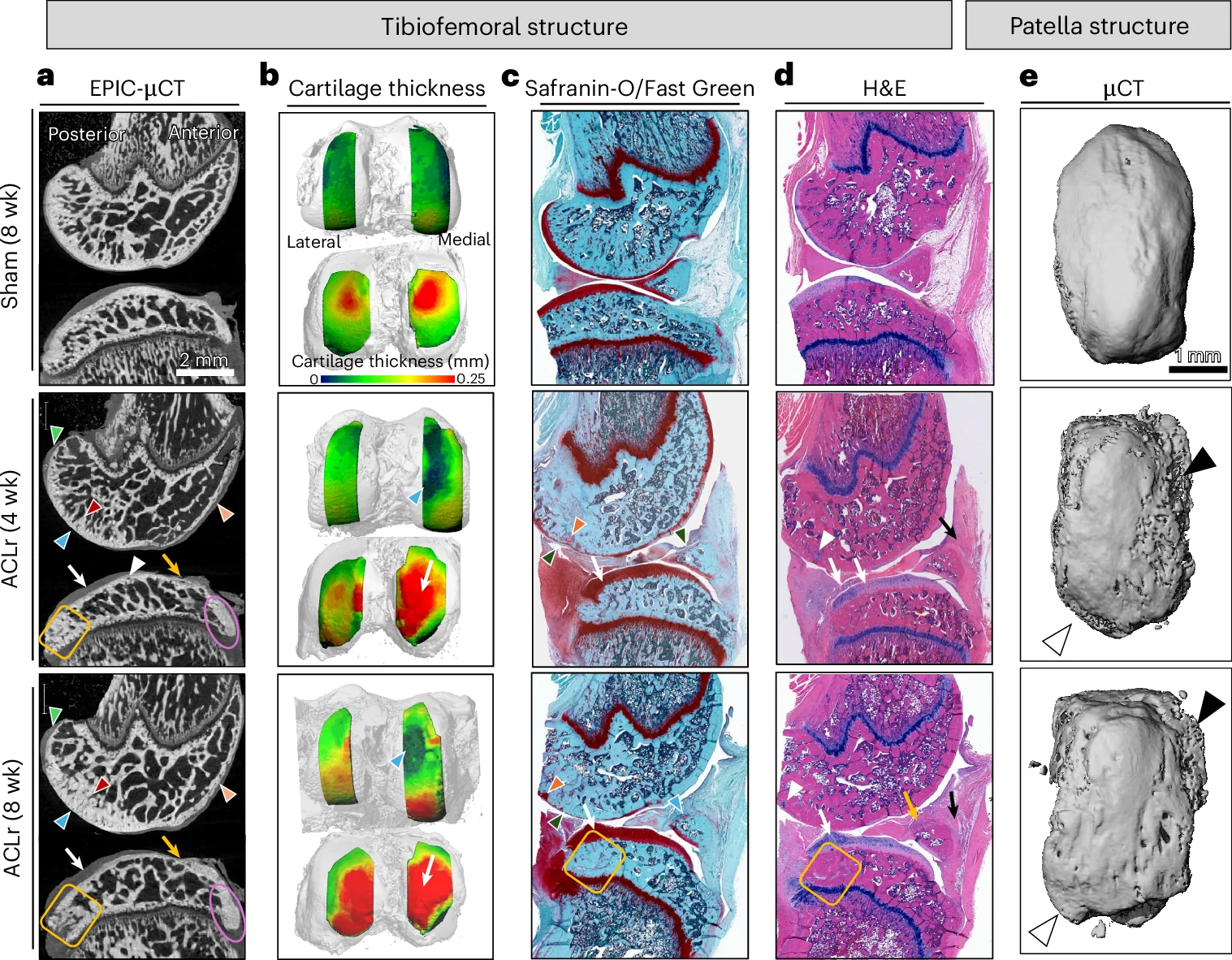
Representative structural pathologies. Structural adaptations throughout the joint in sham controls or 4 or 8 weeks after ACLr. **a**, Representative 2D tomograms bisecting the medial tibiofemoral compartment. ACLr induced focal changes in femora, with posterior cartilage thickening (green triangle), cartilage lesions (blue triangle), subchondral sclerosis (red triangle) and thinning (orange triangle). Tibia had cartilage thickening (white arrow), degeneration of the posterior medial horn (yellow box), widespread osteophyte formation (yellow arrow) and remodeling of the anterior tibial cap (purple circle). **b**, Medial and lateral tibial plateaus and femoral condylar cartilage thickness maps overlaid onto the corresponding epiphyseal bone following µCT reconstruction. Tibia had cartilage thickening (white arrow) and femora exhibited cartilage lesions (blue triangle). Green, thinnest cartilage regions; red, thickest cartilage regions (maximum of 0.25 mm thickness). **c**, Safranin-O/Fast Green histological stain. Femora had reduced proteoglycan content on the medial condyle (red stain) and bone lesions (orange triangle). Tibia displayed increased proteoglycan content on the medial plateau and posterior bone densification (yellow box). Menisci had reduced proteoglycan content on the articulating surface (dark green triangle). Red stain, proteoglycan content; blue, collagen or mineral. **d**, Hematoxylin and eosin (H&E) histological stain. Femora had loss of cells on the articulating condylar cartilage and bone lesions (white triangle). Tibia had cellular hypertrophy and hyperplasia in the posterior cartilage (increased eosin staining intensity), along with cartilage swelling (white arrow). Meniscal calcification began at 4 weeks and was obvious cranially on the anterior aspect of the medial compartment by 8 weeks (yellow arrow), along with synovial hyperplasia (black arrow). **e**, Computed tomography reconstruction of patella with osteophytes (white triangle) and bone resorption (black triangle) evident at 4 and 8 weeks. EPIC-µCT, equilibrium partitioning of ionic contrasting agent µCT. wk, week.

Degenerative PTOA-associated changes were observed in tibiae and femora (Fig. 3a) at 4 and 8 weeks post-injury, with no pathology in sham. Posterior subchondral bone initially exhibited thinner subchondral plates at 4 weeks before developing into a sclerotic phenotype at 8 weeks (red triangle). Full-thickness cartilage lesions were correspondingly observed in the regions of subchondral remodeling (blue triangle) and in the cartilage thickness heatmaps (Fig. 3b). Cartilage appeared thicker in the regions directly adjacent to the observed lesions and was most prominent in the posterior region (green triangle). Roughening of the subchondral plate at the cartilage–bone interface toward the anterior aspect of the medial femoral condyle was first noted at 4 weeks and was more severe at 8 weeks (orange triangle).

#### Tibia

Remodeling of the posterior tibial horn was observed at 4 and 8 weeks post-injury (reduced trabecular structure) (Fig. 3a, yellow square). By 8 weeks, resorption of this same region was clear by the interdigitation of surrounding connective tissue (yellow square). Anterior subchondral bone at 4 weeks developed into marginal tissues (osteophytes) by 8 weeks (yellow arrows), along with extension of anterior subchondral bone toward the proximal aspect of the tibia (purple circles). Cartilage thickened in the posterior medial and lateral plateaus at 4 weeks and continued to increase through 8 weeks (white arrow), which was corroborated with the cartilage thickness heatmaps (Fig. 3b).

#### Whole-joint histology

At 4 and 8 weeks post-injury, proteoglycan staining (Fig. 3c, pink stain) and cellularity (Fig. 3d, purple stain) were severely diminished in the medial posterior femoral condyle compared with sham. Conversely, increased proteoglycan content was observed in the posterior medial tibial plateau (Fig. 3d) in the same region of thickened cartilage noted in the tomograms. Synovial hypertrophy, thickening of the joint capsule and meniscal ossification were clearly observed in injured limbs in both posterior and anterior regions (Fig. 3c,d). Representative histological evidence of ACLr was confirmed in additional sections in the coronal plane, along with collagen fibrillation and mineralization of the meniscus (Supplementary Fig. 1a,b).

#### Patella

ACLr induced a phenotypic change in patella from a smooth oval morphology phenotype toward a more rectangular, jagged geometry starting at 4 weeks, which was heightened by 8 weeks (Fig. 3e). Porous changes to the external cortical bone highlight bone resorption (black triangle); marginal osteophytes were also observed at 4 and 8 weeks post-injury (white triangle).

### Structural morphometric quantifications

#### Femur

**Fig. 4:**
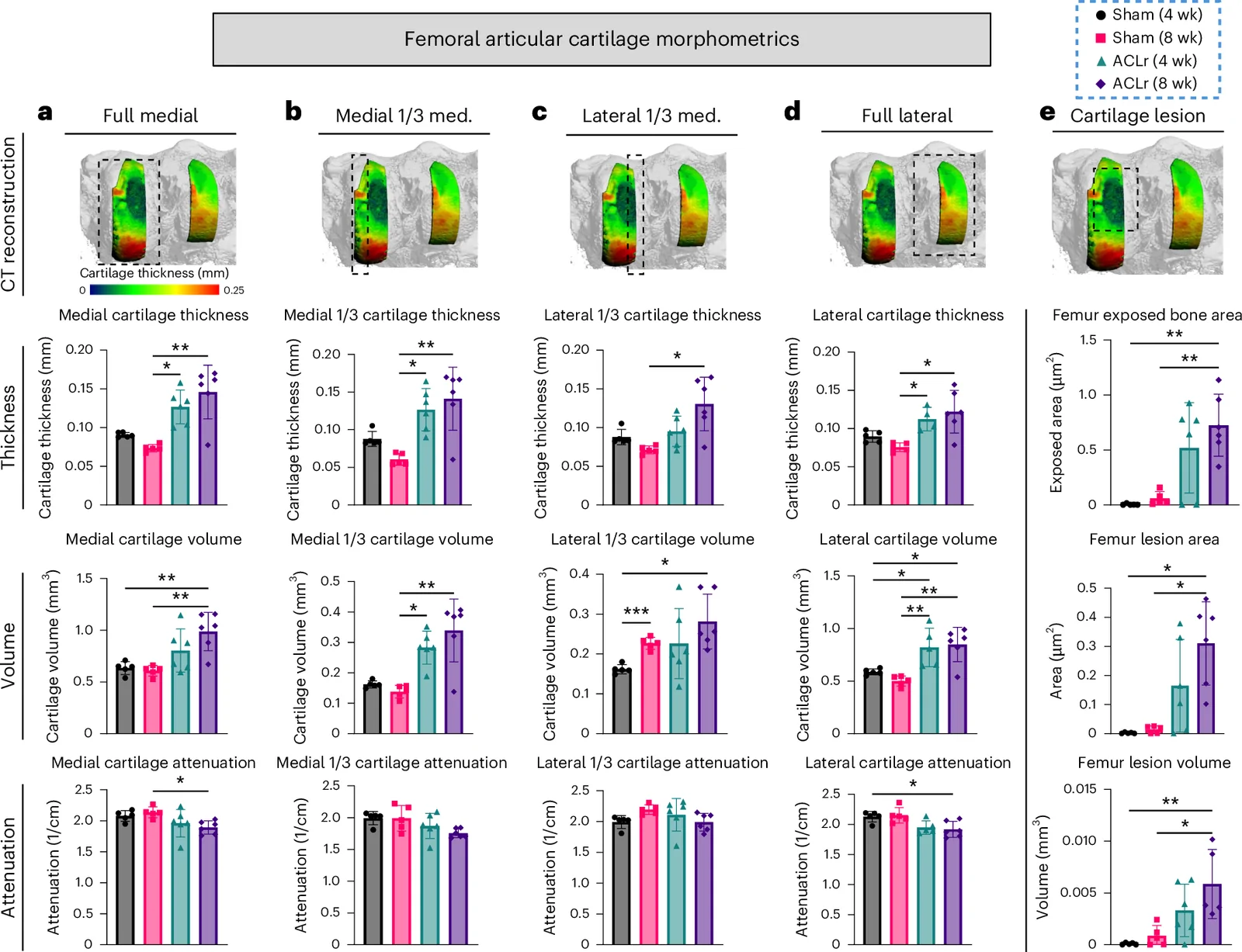
Femoral structural morphometric quantifications. **a**–**d**, Quantified morphometric analyses of femoral articular cartilage. Cartilage thickness increased along the full medial condyle (**a**) and in both medial (**b**) and lateral (**c**) subcompartments of the medial condyle, as well as throughout the lateral condyle (**d**). **e**, Exposed bone area in the medial condyle. In **a**–**e**, data are presented as mean ± standard deviation (sham, *n* = 5; ACLr, *n* = 6). One-way ANOVA with Tukey’s post-hoc test or Brown–Forsythe and Welch ANOVA tests with Dunnett T3 multiple comparisons test was used, depending upon sample distributions (Supplementary Statistics). *P = 0.05, **P = 0.005, ***P = 0.0005, ****P = 0.00005. med., medial; wk, week.

Cartilage thickness and volume were significantly increased at 8 weeks post-injury compared with sham (*P* = 0.002, *P* = 0.003), and thickness showed a trend toward increase at 4 weeks compared with sham in the full medial femoral condyle, while X-ray attenuation was not affected. (Fig. 4a). Cartilage thickness also significantly increased when the medial or lateral 1/3 subcompartments were isolated in the medial femoral condyles at 8 weeks compared with sham (*P* = 0.043, *P* = 0.039), and both trended toward significance at 4 weeks (Fig. 4b,c). In the full lateral femoral condyle, cartilage volume and thickness significantly increased at 4 weeks (*P* = 0.042, *P* = 0.048) and 8 weeks compared with sham (*P* = 0.047, *P* = 0.002) (Fig. 4d). Cartilage attenuation decreased at 8 weeks compared with sham (*P* = 0.045) (Fig. 4d), which corresponded to the increased histological red staining (proteoglycans) observed in the deep cartilage zones surrounding femoral lesion areas (Supplementary Fig. 1a). The medial condyle for ACLr limbs had significantly increased exposed bone area, lesion area and lesion volume at 8 weeks compared with sham (*P* = 0.006, *P* = 0.015, *P* = 0.011) (Fig. 4e).

#### Tibia

**Fig. 5:**
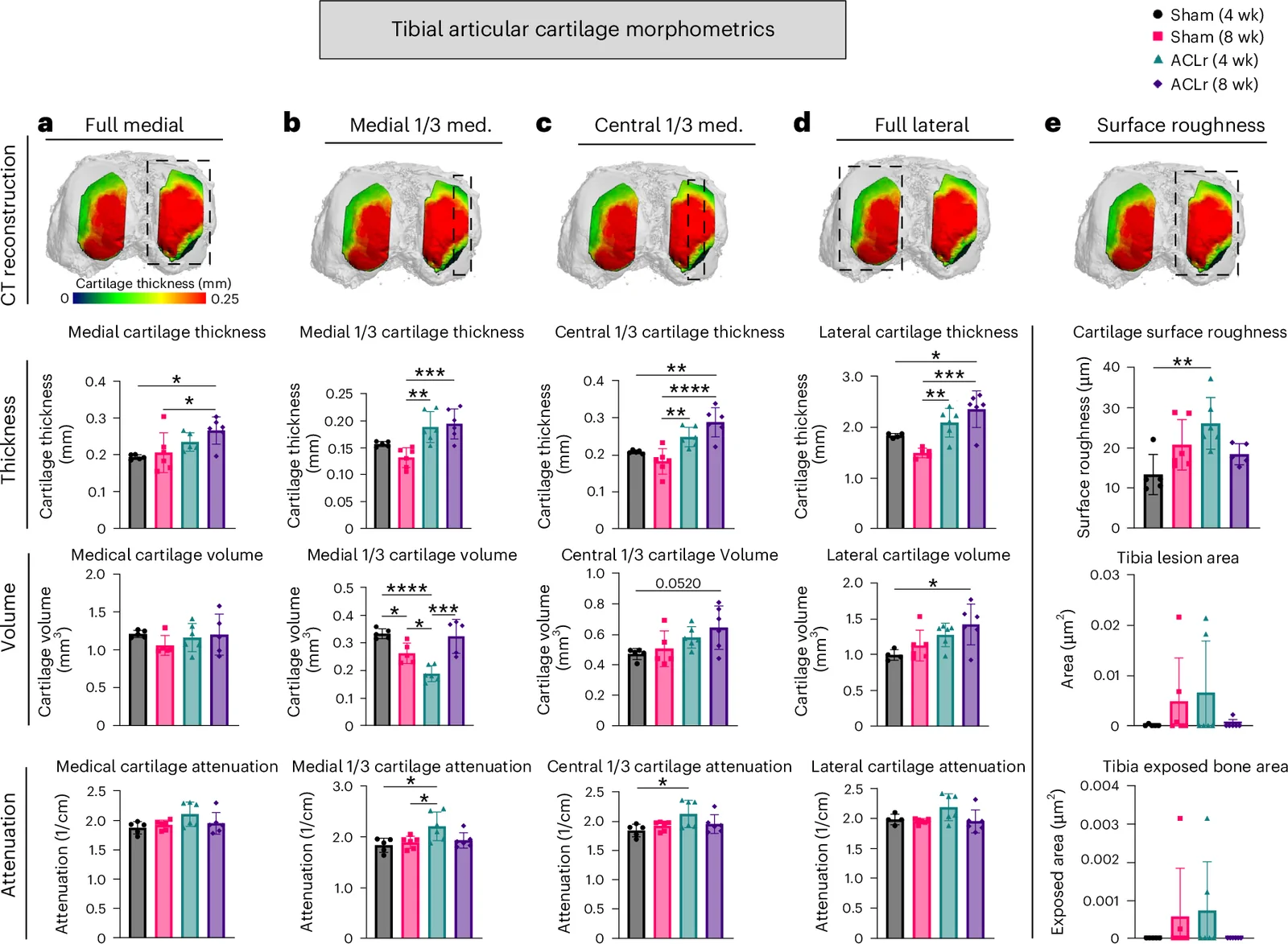
Tibial structural morphometric quantifications. **a**–**d**, Quantified morphometric analyses of tibial articular cartilage. Cartilage thickness, volume and attenuation were not affected across the full medial tibial plateau (**a**), but focal changes in the medial (**b**) and central (**c**) subcompartments captured increased thickness at 4 and 8 weeks, as well as throughout the lateral condyle (**d**) at 8 weeks. **e**, Surface roughness significantly increased at 4-weeks with no measurable lesions. In **a**–**e**, data are presented as mean ± standard deviation (sham, *n* = 5; ACLr, *n* = 6). One-way ANOVA with Tukey’s post-hoc test or Brown–Forsythe and Welch ANOVA tests with Dunnett T3 multiple comparisons test was used, depending upon sample distributions (Supplementary Statistics). *P = 0.05, **P = 0.005, ***P = 0.0005, ****P = 0.00005. med., medial; wk, week.

Medial tibial cartilage thickness, volume and X-ray attenuation were not significantly affected when considered over the full medial tibial compartment (Fig. 5a). When the medial 1/3 of the medial tibial plateau was isolated and evaluated, cartilage thickness increased at 4 weeks and 8 weeks compared with sham (*P* = 0.002, *P* < 0.001) along with X-ray attenuation at 4 weeks (*P* = 0.018) (Fig. 5b). Cartilage volume decreased at 4 weeks (*P* < 0.001) compared with sham but was no longer significantly different at 8 weeks (Fig. 5b). When the central 1/3 of the medial tibial plateau was separately isolated, cartilage volume was unaffected, but cartilage thickness increased at 4 weeks and 8 weeks compared with sham (*P* = 0.001, *P* < 0.001) (Fig. 5c). In the full lateral tibial plateau, cartilage thickness and volume increased significantly at 8 weeks compared with sham (*P* < 0.001, *P* = 0.025) and cartilage attenuation trended towards an increase at 4 weeks compared with 8 weeks (Fig. 5d). Tibial cartilage surface roughness throughout the full medial plateau increased significantly by 4 weeks (*P* = 0.005) compared with sham, and there were no quantifiable lesions (Fig. 5e).

#### Bone and mineral morphometrics

**Fig. 6:**
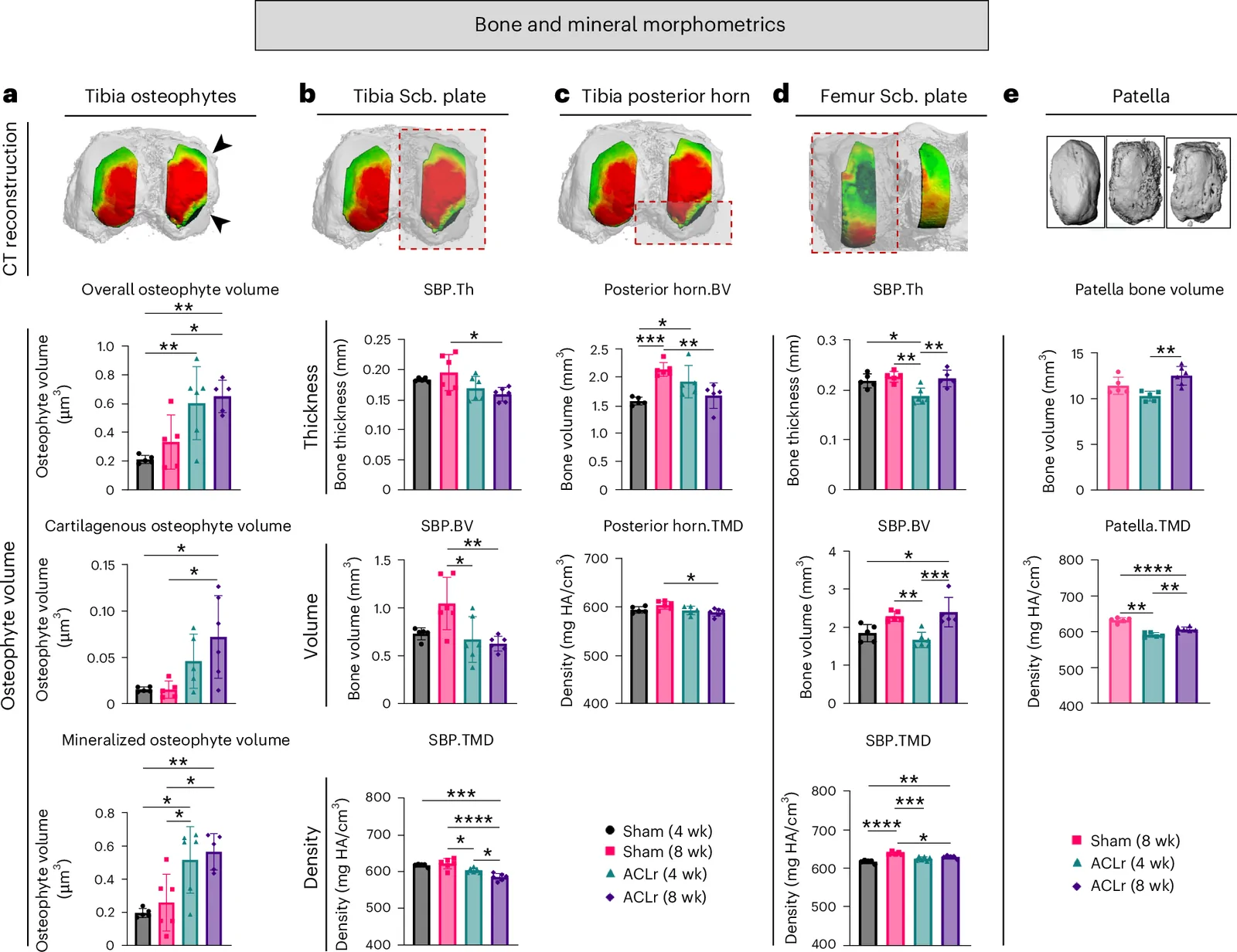
Bone and mineral morphometrics. **a**, Tibial osteophytes (black arrowheads) were significantly increased at 4 and 8 weeks. **b**, Tibial subchondral plate thickness (SBP.Th), volume (SBP.BV) and total mineral density (SBP.TMD) reduced over time. **c**, The posterior tibial horn had decreased bone volume starting at 4 weeks, and reduced density by 8 weeks, which is in good agreement with observed structural pathologies. **d**, Femoral subchondral plate thickness, volume and density decreased at 4 weeks. **e**, Patella bone volume decreased at 4 weeks and significantly increased at 8 weeks, while bone density decreased at both time points, with a greater reduction at 8 weeks compared with 4 weeks. The 4-week sham patella were lost during tissue isolation and excluded. In **a**–**e**, data are presented as mean ± standard deviation (sham, *n* = 5; ACLr, *n* = 6). One-way ANOVA with Tukey’s post-hoc test or Brown–Forsythe and Welch ANOVA tests with Dunnett T3 multiple comparisons was used, depending upon sample distributions (Supplementary Statistics). *P = 0.05, **P = 0.005, ***P = 0.0005, ****P = 0.00005. HA, hydroxyapatite; Scb., subchondral; wk, week.

Tibial mineralized, cartilaginous and overall osteophyte volumes increased both at 4 weeks (*P* = 0.005, *P* = 0.008, *P* = 0.021) and 8 weeks (*P* = 0.005, *P* = 0.021, *P* = 0.046) compared with sham (Fig. 6a). Tibial subchondral plate thickness (SBP.Th) and volume (SBP.BV) decreased at 8 weeks compared with sham controls (*P* = 0.021, *P* = 0.015), while tissue mineral density (SBP.TMD) decreased at 8 weeks compared with sham controls (*P* < 0.001) and 4-week ACLr (*P* = 0.016). (Fig. 6b). Medial posterior horn bone volume significantly increased at 4 weeks compared with the sham (*P* = 0.049) but then comparatively decreased at 8 weeks (*P* = 0.004) (Fig. 6c). Total mineral density (Posterior Horn.TMD) decreased at 8 weeks (*P* = 0.021) and trended toward significance at 4 weeks (Fig. 6c). Femur subchondral plate thickness and volume decreased at 4 weeks (*P* = 0.014, *P* = 0.015) (Fig. 6d). Overall, patella bone density (Patella.TMD) significantly decreased at 4 weeks and 8 weeks compared with sham (*P* < 0.001, *P* < 0.001), and density at 4 weeks was significantly less than at 8 weeks (*P* = 0.008). Patella bone volume at 4 and 8 weeks post-injury was not different from sham but was significantly greater at 8 weeks than at 4 weeks (*P* = 0.003) (Fig. 6e).

### Dimensionality reduction statistical model of key OA structural and functional parameters

**Fig. 7:**
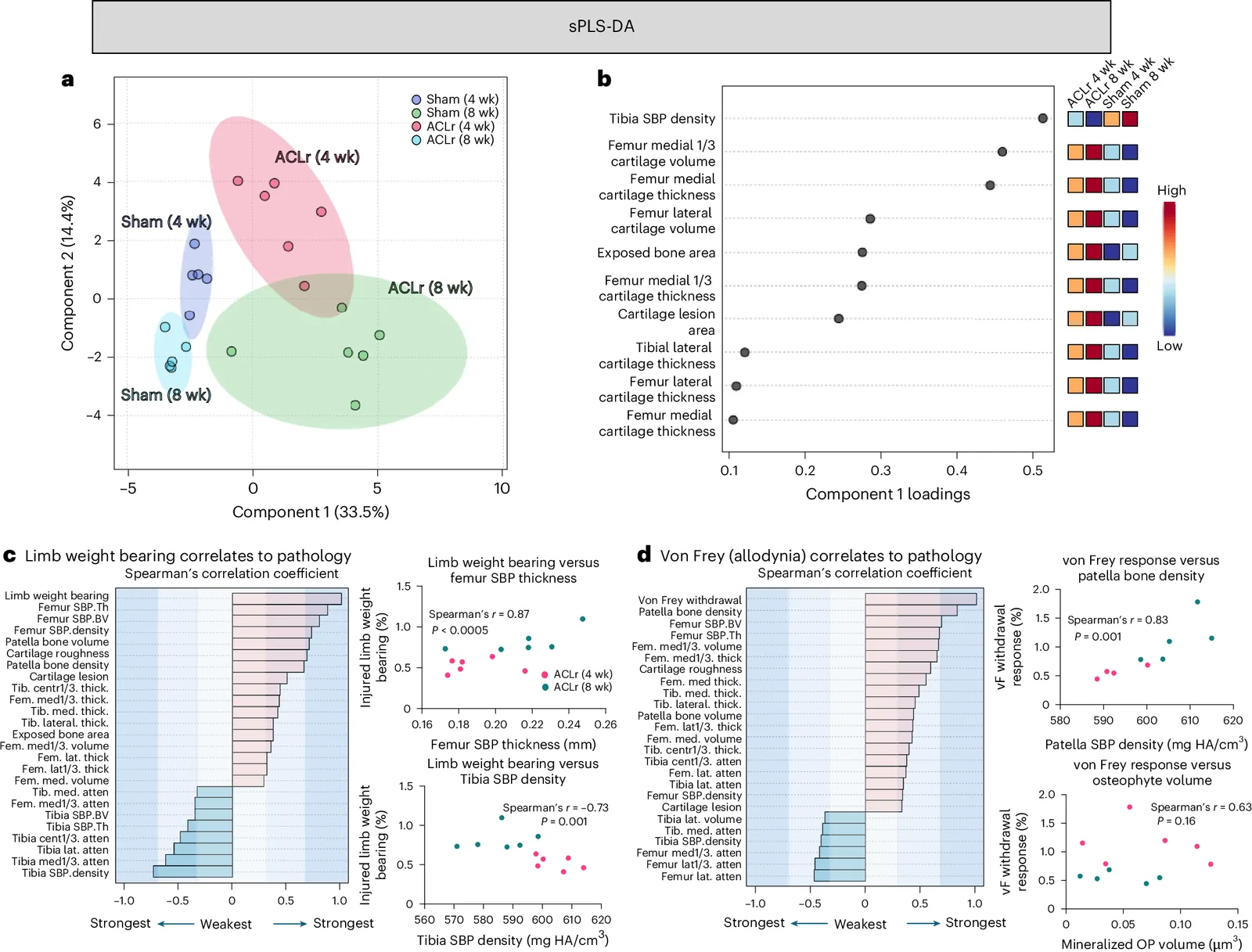
Dimensionality reduction statistical model. Identifying key pathologies via sPLS-DA and Spearman’s correlations. **a**, ACLr animals separated from sham controls along component 1 on the scores plot (data presented standardized with 95% confidence intervals). Early-moderate and moderate-severe disease pathologies were separated along component 2. **b**, Loadings plot for component 1 describing tibial SBP density and femoral cartilage degeneration as the most important pathological features when separating diseased and healthy animals. **c**, Injured limb weight-bearing function correlated with femoral and tibial SBP bone pathologies (ACLr, *n* = 6 per group). **d**, Pain sensitization correlated with patella bone pathology and femoral cartilage degeneration but not osteophytes (ACLr, *n* = 6 per group). Statistically significant correlations reported (*P* < 0.05). OP, osteophyte; vF, von Frey; wk, week.

Groups with OA pathology (green, red) clearly separated from sham controls (purple, blue) along component 1 with sparse partial least squares-discriminant analysis (sPLS-DA), which accounted for ~33% of the total observed variance (Fig. 7a). Distinct clusters for moderate and severe OA progression separated along component 2 and highlight a change in pathology throughout the joint with disease progression. Changes in tibial subchondral plate bone density and medial femur cartilage degeneration parameters had the greatest weight in the loading plot for component 1 (Fig. 7b). Diseased tibial and femoral subchondral plate bone parameters (density, volume and thickness) had statistically meaningful (*P* < 0.05) strong monotonic correlations with weight-bearing function (Fig. 7c and Supplementary Fig. 2a,b). However, the medial femur cartilage parameters identified as important features from the sPLS-DA loading plots showed weak-to-mild and nonsignificant correlations with limb weight-bearing function. Femoral and patella bone parameters had statistically meaningful (*P* < 0.05) moderate-to-strong monotonic correlations with pain sensitization (Fig. 7d). For pain sensitization, the medial femur cartilage parameters were also moderately strongly correlated. Osteophytes did not have a statistically meaningful correlation with any pain-related behaviors.

## Discussion

The objective of this work was to comprehensively characterize OA pathology after ACL injury in a rat model, identify key pathophysiological features during disease progression and describe the relationships between structural pathology and pain and function. ACLr increased hindlimb pain sensitivity and impaired limb weight-bearing function, both of which were strongly correlated with bone pathologies in multiple joint compartments. Animals exhibited increased paw withdrawal responses at 4 and 6 weeks that were resolved to sham levels by 8 weeks, which is indicative of limb hyperallodynia. Chronic weight-bearing deficiency and asymmetry were measured over 8 weeks, suggesting continued offloading to the contralateral uninjured hindlimb. These data describe a small animal model of PTOA that captures an acute phase of injury and early disease at 4 weeks after ACLr and transitions to a chronic disease phenotype and severe pathology by 8 weeks. This agrees with observations in patients with PTOA that experience hypersensitivity and pain during weight bearing in the disease initiation phase. Only a single study has previously assessed limb sensitivity after non-invasive ACLr in rats, which, in contrast to our results, reported a prolonged increase in von Frey responses through 12 weeks^21^.

Our work also corroborates behavioral pain findings in surgically induced rat models (for example, the medial meniscal transection (MMT) surgery), although with more profound behavioral adaptations. One MMT study in mature Sprague-Dawley rats found significantly reduced paw withdrawal responses and weight bearing in injured limbs through the endpoint (4 weeks), although reductions in weight bearing were less dramatic than the ones we recorded at that same time point (that is, ~10% reduction in weight bearing after MMT compared with ~50% after ACLr)^23^. Similar responses were recorded in seminal MMT work with male Lewis rats that also showed no effect of MMT-induced OA on mechanical hyperalgesia or thermal allodynia^13^. Prolonged hyperallodynia and weight-bearing dysfunction has also been observed after surgical ACL transection in Wistar rats through 18 weeks after injury^24^. Our work improves the understanding of how pain sensitization and behaviors develop in the context of ACLr, which is a leading risk factor for developing PTOA^25,26^. Together, these data suggest the importance of studying the effects of disease on pain behaviors, especially as a measure of therapeutic efficacy. It is well regarded in the clinic that patient pain does not strictly correlate with structural tissue disease pathology; this underscores the need to study relationships between pain-related behaviors and structural tissue changes within the context of preclinical models of PTOA progression. The relatively limited literature characterizing the relationship between pathology and function has primarily used moderate knee compression rather than a more clinically relevant full rupture, and has attempted to compare semi-quantitative histological techniques in a single joint region with von Frey responses, rather than performing quantitative analyses across specific joint compartments^26^. Our work expands upon earlier work by Heegde et al. showing that histological OA scores correlate with von Frey responses by identifying subchondral bone plate pathology in the medial tibiofemoral compartment as a key feature associated with pain behaviors (von Frey and limb weight-bearing) after compressive knee injury in mice^27^. This is in good agreement with a growing body of works showing that pathological nerve sprouts colocalize with tibial subchondral sclerosis and osteophyte formation^28–30^. Moreover, we show that patella pathology correlates strongly with pain sensitivity and limb function.

High-resolution µCT is a powerful tool for quantifying hard tissues in small animal models, such as rats, and can be combined with contrast agents for quantifying soft tissue pathologies^31^. A wide body of literature has shown the utility of this technique for quantifying knee joint bone and cartilage changes to investigate both fundamental disease phenotypes and prospective disease-modifying OA drugs^14,16,19,20,32–38^. Little work has leveraged contrast-enhanced µCT for applications in non-invasive knee injury models for PTOA. This work leverages both contrast-enhanced µCT and behavioral assays to correlate quantitative tissue pathology to pain and function. Previously, Maerz et al. provided the first contrast-enhanced µCT characterization of murine (rat) knee joints after a non-invasive ACLr to cause PTOA and compared it with surgical ACL transection^19,20^. Our cartilage and bone pathologies are consistent with this work but improve upon it by adding distinguishing OA pathologies, such as osteophytes and cartilage lesion metrics, which are highly relevant to clinical imaging diagnoses. To do this, we used well-established volumes of interest (VOIs) in rat PTOA models^14,16,19,20,32–38^ and also developed novel VOIs specifically tailored to capture pathology unique to ACLr.

The medial tibiofemoral compartment accumulates degenerative changes in both preclinical models and clinical populations^16,39–41^. Our model captured these changes in articular cartilage, osteophyte formation and subchondral bone remodeling as early as 4 weeks after ACLr. Quantitative consideration of a full suite of bone and cartilage parameters in tibia, femur and patella compartments led us to generate a sPLS-DA model to identify the key features in distinguishing moderate and severe stages of OA disease progression. We show that tibial subchondral plate density is a clear discriminator of both early OA disease initiation and severity progression, and that cartilage degeneration in the femur rather than tibia may be particularly relevant for ACLr preclinical studies. Degeneration was most severe in the posterior aspect of the medial tibial plateau and femur, which is observed in human clinical populations with ACL deficiency and subsequent subluxation of the proximal tibia relative to the femur (that is, anterior tibial ‘thrust’)^39,42,43^. We also quantify full-thickness cartilage lesions on the medial femoral condyle, which remain poorly described in clinical settings. These lesions and corresponding regions of subchondral sclerosis may serve as critical VOIs to consider when investigating novel therapeutics for PTOA. This work is in agreement with previous observations in this model by Maerz et al., who showed that VOIs should be carefully selected to capture focal changes in cartilage and bone thickening and thinning that are overlooked when performing whole-joint analyses^19^.

This work presents an analysis of chronic patellar degeneration at early and severe stages of disease. Patellofemoral pain and degeneration frequently accompany OA^44^ but it is widely ignored in preclinical animal models or restricted to cartilage changes in the trochlear groove. Only a single study has previously investigated the patella in a preclinical non-invasive joint destabilization model and in the acute phase (2 weeks) after injury^18^. Given the importance of the entire joint in disease propagation, and in light of recent studies demonstrating that the infrapatellar fat pad is a mediator of joint disease^45–47^, the poor quality of patellar bone and strong correlation with pain sensitization identified here warrant further investigation as a potential therapeutic target.

Although this study provides a comprehensive analysis of joint degeneration following preclinical ACLr, it has some limitations. As with most non-invasive injury models, it remains unclear whether concomitant joint microdamage occurs during injury induction that may contribute to intersubject variability; however, we aimed to minimize this limitation via rigorous injury characterization in the preliminary pilot work (Supplementary Methods 1 and Supplementary Fig. 3). Furthermore, functional and pain-related behavioral assessment is challenging and influenced by a number of external factors, which have been reviewed in detail^48^. Confounding factors were minimized by maintaining consistent testing conditions and having the same operator perform von Frey and weight-bearing tests at all time points (for example, always at the end of the ‘dark cycle’, same room light and temperature settings) and in a blinded and randomized order. Other limitations for interpreting pain-related behavior in Lewis rats may include an altered hypothalamic–pituitary–adrenal axis and corresponding heightened pain responses compared with outbred rat strains^49^. Our results are reported as relative changes from baseline measurements to account for innate differences between animals. Another potential weakness is that quantitative morphometrics were not directly compared with histological scoring^50^, although this comparison was not a primary goal of this study and would have required much higher animal numbers to achieve statistical power. Follow-up studies should further consider histopathological and immunohistochemistry to elucidate underlying molecular mechanisms. In addition, sex-based differences were not accounted for in this study design and may limit generalization of these conclusions. Future studies should apply this model to female rats to investigate how sex-based differences may affect relationships between structural disease progression and pain perception. Another limitation of this work may include generalizability of these findings to disease progression after ACL reconstruction, which was not assessed in this study; however, it is important to note that surgical reconstruction does not alter rates of PTOA development^51^. Despite these limitations, we believe these findings provide valuable insight into PTOA after ACLr.

Ultimately, this work establishes foundational quantitative methods for measuring OA progression after a physiologically relevant ACL injury in a preclinical rodent model and ties them to aberrant pain and function. These metrics can be used for investigating the effects of potential disease-modifying treatments on hyperallodynia, functional limb asymmetries and cartilage and bone degeneration in a variety of joint tissues at multiple disease stages. Future studies should leverage these techniques and expand this model to include relevant physical rehabilitation interventions and study fundamental joint disease etiology.

## Methods

### Animal model and study design

Skeletally mature (14-week-old) male Lewis rats (Charles River Laboratories) were selected for their propensity to develop OA in previous knee injury models^19,20,22^ and surgical destabilization models^13–16^. Females were excluded from this study because male Lewis rats at this age are notably heavier than females, which likely results in different disease progression timelines in this model. The heavier males represented a desirable starting point for characterizing disease progression in the ACLr model because of a potentially more rapid onset of disease pathology associated with greater body weights and joint loading forces. Animals were cohoused (two animals per cage) with ad libitum food and water access in a temperature-controlled animal facility on a 12-h light/dark cycle with standard housing (Supplementary Methods 1). All animal work was conducted under the University of Oregon Institutional Animal Care and Use Committee-approved protocols (21–19). Baseline pain-related behavioral measurements (weight-bearing behavior and von Frey) were collected 1 day before ACLr (following a 10-min acclimation period) and repeated 4, 6 and 8 weeks post-injury to assess OA disease progression (Fig. 1a). Animals were euthanized at 4 weeks (early-moderate PTOA phenotype) or 8 weeks (moderate-severe PTOA phenotype).

A nonsurvival pilot experiment (*n* = 10) was initially conducted to characterize injury mode, location and rupture forces (Supplementary Fig. 3). Based on these results, a sample size of *n* = 10 for ACLr was selected (longitudinal measures: *n* = 10 per group for pain-related behaviors; post-mortem measures: *n* = 6 computed tomography, *n* = 4 histology) and *n* = 7 for sham (longitudinal measures: *n* = 7 per group for pain-related behaviors; post-mortem measures: *n* = 5 computed tomography, *n* = 2 histology). Treatment groups were randomized via a random number generator (Microsoft Excel) on the day of the procedure, and extended-release buprenorphine 0.5 mg/kg (ZooPharm Wildlife Pharmaceuticals) was administered subcutaneously before injury. Male Lewis rats (340 ± 11 g) were anesthetized with 3–5% isoflurane and maintained via nose cone at 1.5–3.0% throughout the procedure. Animals were placed in dorsal recumbency upon a custom machined platform with the ankle tucked into a mounting recess. A three-dimensionally printed knee cup was lowered to hold the knee in flexion^22^. Custom software^22^ (Labview, National Instruments) was used to control uniaxial loading through the knee cup via a pneumatic piston. A static preload (5 N) was first applied to the left hind limb and maintained for 30 s, followed by a loading ramp of 5 N/s until either the distal femur cranially translated out of the knee pocket or a maximum load of 65 N was achieved. All animals ruptured at or below 65 N in this study. Translation of the tibia frontally was confirmed immediately after loading in each animal via video recording and playback. Joint laxity and internal–external tibial rotation were assessed before and immediately after injury. Sham animals received only the 5 N static preload. Animals were monitored every 24 h through 5 days post-injury with a health history form (outward appearance, weight, mentation and grimace characterization) and repeated weekly thereafter. At 4 weeks, ACL injury (*n* = 10) and sham (*n* = 7) rats were randomly selected for euthanasia, and tissues were collected; the remaining sham and injury animals were euthanized at 8 weeks. Euthanasia occurred via CO_2_ asphyxiation, followed by a secondary method of cervical dislocation, and confirmed by the absence of vital signs. No animals were excluded or euthanized early, based upon humane endpoint criteria (loss of >20% body weight, lameness or clinical dehydration).

### Pain-related spontaneous weight-bearing behavior

Pain-related weight-bearing behavior was assessed via the Bioseb DWB 2.0 system (Bioseb). Rats were allowed to freely roam in a transparent cage for 5 min, during which an integrated video monitoring system and high-resolution force sensor array quantitatively recorded weight distribution for each paw. Automated scoring was manually validated by the same researcher blinded to treatment groups. Weight bearing on each paw is reported as a percentage of individual animal weight (wt%) or a ratio of weight bearing on the ipsilateral/contralateral hindlimbs (as a measure of weight-bearing asymmetry) and relative to the baseline weight-bearing measurements to account for behavioral differences between animals. Weight bearing was assessed immediately before von Frey testing to avoid the confounding effects of limb sensitization.

### Mechanical (tactile) allodynia: electronic von Frey

Pain-related sensitization was evaluated in both ipsilateral and contralateral hindpaws using the Bioseb Electronic von Frey system (EvF) (Bioseb) with measures at baseline (1 day before surgery), 4 weeks, 6 weeks and 8 weeks post-injury. Animals were placed into clear acrylic enclosures atop a stainless-steel mesh grate and underwent a 10-min acclimatization period. Afterwards, an instrumented electronic von Frey filament was slowly and continuously applied to the flat plantar region of the hindlimb during stationary behavior until eliciting a response (determined as full or partial withdrawal of the hindlimb), and the final response force was recorded. Each limb (ipsilateral, contralateral) was recorded in triplicate with a restoration period between limbs (~10 min). Results for each limb are reported relative to individual baseline measures to account for behavioral differences between animals. Each testing time point was conducted by the same trained researcher blinded to the treatment group for each animal to minimize inter-researcher variability, and all recordings were conducted at the same time of day (end of light cycle).

### Knee joint structural pathology

#### Contrast enhanced μCT

Knee joint tissues (*n* = 6 per group) were dissected immediately post-mortem and fixed in 10% formalin. Surrounding soft tissues of the tibiae, femora and patellae were carefully removed, and the proximal ends of the tibiae and femora were immersed in a 37.5% Conray (iothalamate meglumine) solution (62.5% ion-free phosphate-buffered saline) for 30 min^52^. Equilibrium partitioning of ionic contrasting agent µCT (EPIC-μCT) was used to measure cartilage degeneration and osteophyte formation^16^. Tibiae and femora were scanned via a Scanco VivaCT80 (Scanco Medical) at 45 kVp, 177 μA, 8 W and 16 μm resolution in a randomized order. Patellae were scanned without the use of a contrast agent at the same settings. Scanning and evaluation sample order was randomized and conducted blinded to the treatment group.

#### Evaluation

Medial and lateral articular cartilage was contoured in the sagittal plane and globally thresholded from bone and air to quantify cartilage volume, cartilage thickness and attenuation (a measure inversely proportional to glycosaminoglycan content^31^). Medial tibiofemoral compartments were further subdivided into medial, central or lateral regions. Tibial osteophytes were manually contoured in the coronal plane and thresholded for either cartilage or bone to yield a cartilaginous and mineralized osteophyte volume. Quantitative evaluations included assessments of average articular cartilage thickness, volume and attenuation (a surrogate metric of proteoglycan loss), osteophyte volumes (total, mineralized and cartilaginous), surface roughness and exposed bone area. Bone volume and total mineral density (TMD) in the tibial horn and tibial and femoral medial subchondral plate (SBP) were manually contoured and evaluated (Supplementary Methods 2 and Supplementary Fig. 4). Total patellar bone volume and density were determined with standard evaluation scripts. Serial sagittal sections of femora and tibiae were exported as two-dimensional (2D) grayscale TIFF images and processed with a custom MATLAB R2023a (MathWorks) algorithm to calculate cartilaginous surface roughness, exposed bone area, lesion area and lesion volume^34^ (Supplementary Fig. 4). In brief, cartilage regions of interest were fitted with a three-dimensional polynomial surface (quadratic–quartic), and the root mean square error of the deviation between the fitted surface and the articular cartilage surface was defined as the roughness (Sq) of the articular cartilage surface. Tibiae and femora were stored in ion-free phosphate-buffered saline following scanning.

### Representative whole-joint histology

Hindlimbs (*n* = 4 per ACLr group, *n* = 2 sham) were dissected and fixed in 10% formalin. Whole joints were decalcified in Immunocal (10% formic acid), trimmed, paraffin embedded and sectioned in the coronal or sagittal plane through the cruciates (5 μm slices per section; Supplementary Methods 3) (Inotiv). Slides were stained with hematoxylin and eosin (H&E) or safranin-O Fast Green and imaged at 10× with a Leica Aperio Versa 200 Slide Scanner (Leica Biosystems).

### Statistical methods

Univariate statistical analyses were conducted in GraphPad Prism 10.3.0 (GraphPad Software). For analyses involving multiple factors (weight-bearing behavior and electronic von Frey) such as time points and treatments, a mixed-effects model (repeated measures) was fully fit without a Geisser–Greenhouse correction followed by Tukey’s multiple comparison for post-hoc analysis. Full statistical parameters for null hypothesis testing (*F* statistic, confidence intervals, effect sizes, degrees of freedom and *P* values) are reported for each test (Supplementary Statistics). For morphometric analyses, one-way analysis of variance (ANOVA) was used when data were normally distributed with equal standard deviations, followed by Tukey’s post-hoc test. When standard deviation was not equal, Brown–Forsythe and Welch ANOVA tests were used, followed by Dunnett T3 multiple comparisons. When residuals were not normally distributed, the Kruskal–Wallis test followed by Dunn’s multiple comparison was used. Monotonic relationships between pain-related behaviors and tissue pathology were assessed in ACLr animals via Spearman’s rank correlations to negate assumptions on normality and linearity. Statistical significance was defined as *P* < 0.05. Supervised dimensionality reduction was conducted with sPLS-DA owing to the relatively large feature set compared with the small sample size (Metaboanalyst 6.0)^53^. In brief, treatment type was used for sample classification (for example, Sham+4wk, Sham+8wk, ACLr+4wk and ACLr+8wk), data were standardized and LASSO penalization was applied to select the top ten features. Leave-one-out cross-validation was used to evaluate the classification error rate for each component (Supplementary Methods 4 and 5 and Supplementary Fig. 5). Components explaining the highest variance were evaluated on an *X*–*Y* score plot, with 95% confidence included for each class. Loadings plots were generated for the top five components to indicate pathological parameters driving class separation.

### Reporting summary

Further information on research design is available in the Nature Portfolio Reporting Summary linked to this article.

## Online content

Any methods, additional references, Nature Portfolio reporting summaries, source data, extended data, supplementary information, acknowledgements, peer review information; details of author contributions and competing interests; and statements of data and code availability are available at 10.1038/s41684-026-01762-1.

## Supplementary information

Supplementary Information Supplementary Figs. 1–5, Methods 1–5 and Supplementary Statistics 1.

Reporting Summary

Supplementary Data 1 Reporting to the ARRIVE Guidelines for animal research.

## Data Availability

The data that support the findings of this study are available from the corresponding author upon request.
