# Supplementary material for: Structural osteoarthritis pathogenesis correlates with distinct pain and dysfunction profiles after ACL injury in rats
Source: Lab Anim (NY). 2026 Jul 1;55(9):344–53. doi: 10.1038/s41684-026-01762-1 (PMC13518234; doi:10.1038/s41684-026-01762-1)
Supplement: Supplementary file 1 [file 41684_2026_1762_MOESM1_ESM.pdf]

lab animal

Supplementary information

https://doi.org/10.1038/s41684-026-01762-1

Structural osteoarthritis pathogenesis correlates with distinct pain and dysfunction profiles after ACL injury in rats

In the format provided by the authors and unedited

Supplemental Documentation and Figures:

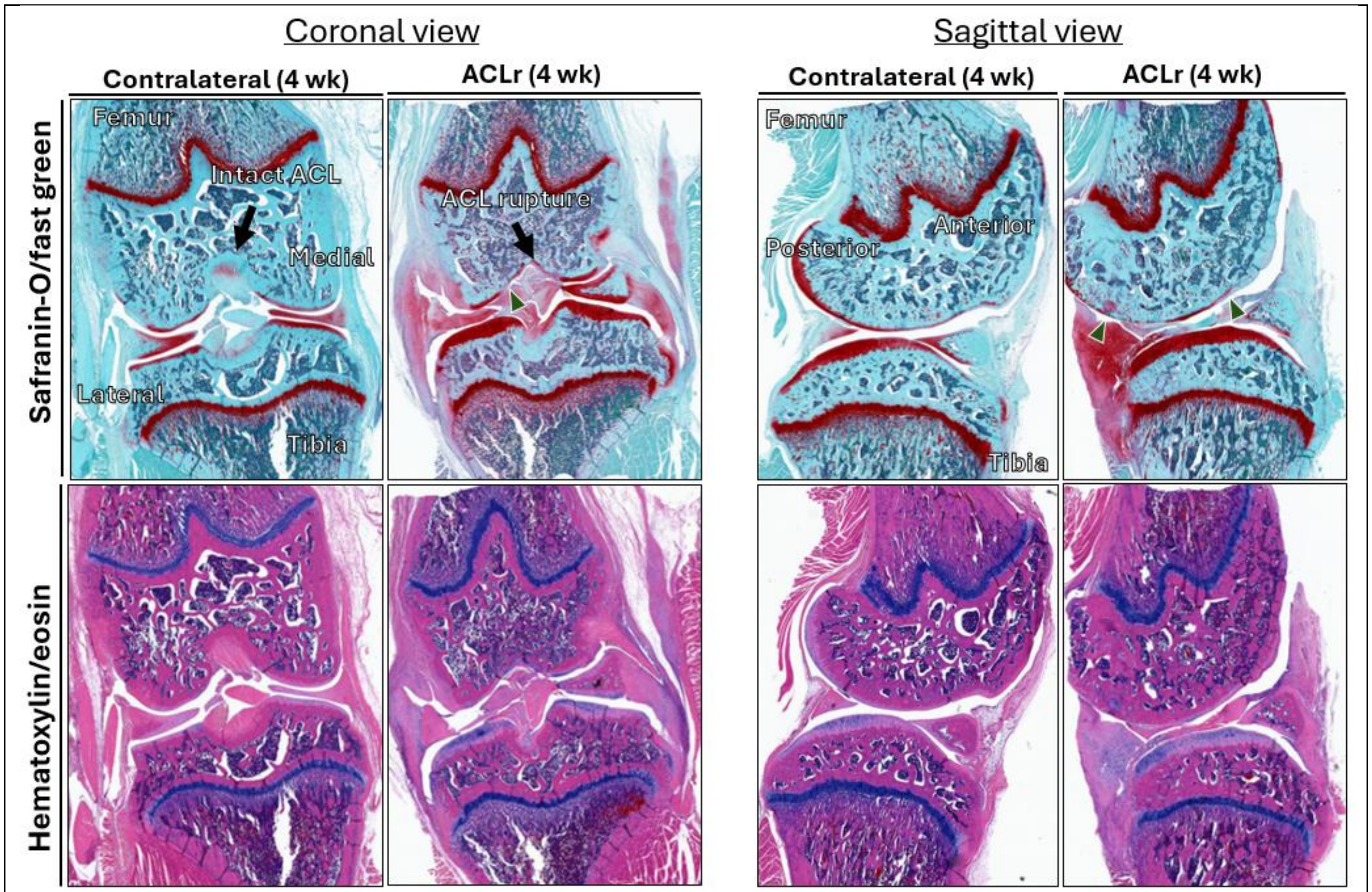

Suppl. Figure 1A: 4-week Safranin-O/fast green and hematoxylin/eosin histological staining of whole tibiofemoral compartments sectioned in the coronal (through the cruciate ligaments) or sagittal (in the medial 1/3 of the medial plateau) in ACLr injured limbs and contralateral uninjured limbs. Coronal view sections show intact versus ruptured ACL (black arrows), and both views show surface proteoglycan loss with ACLr (green triangle).

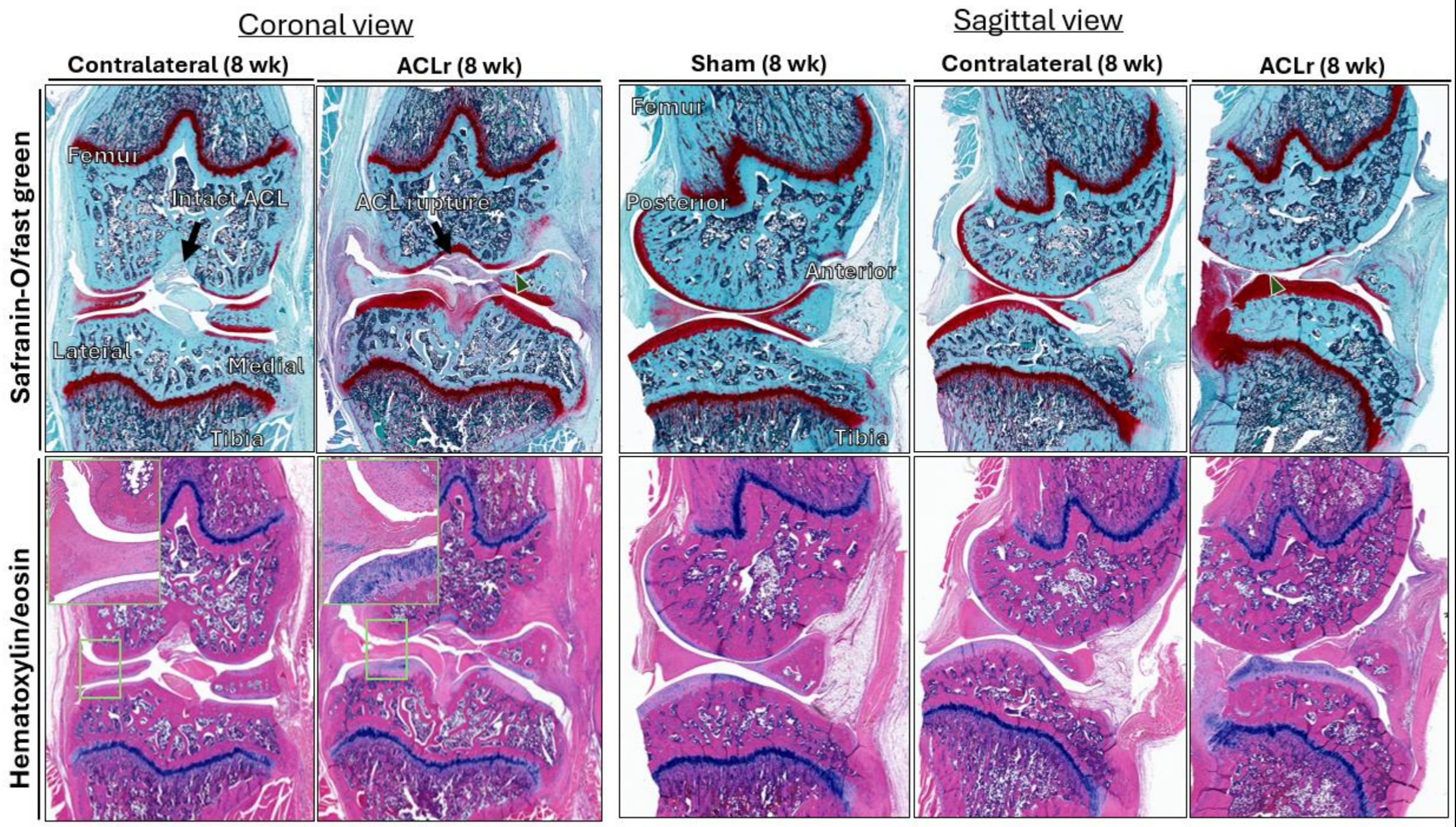

Suppl. Figure 1B: 8-week Safranin-O/fast green and hematoxylin/eosin histological staining of whole tibiofemoral compartments sectioned in the coronal (through the cruciate ligaments) or sagittal (in the medial 1/3 of the medial plateau) in ACLr injured limbs, contralateral uninjured limbs, and compared to Sham control animals (sagittal sections only). Coronal view sections show intact versus ruptured ACL (black arrows) and surface proteoglycan loss (green triangle). hematoxylin/eosin coronal sections at 8-week also include magnified (20x) regions of synovitis and cellular hypertrophy.

Correlation Analysis for morphometrics and pain behaviors

Spearman's Rank Correlation Heatmap for Correlations greater than 0.5 (ρ>0.5)

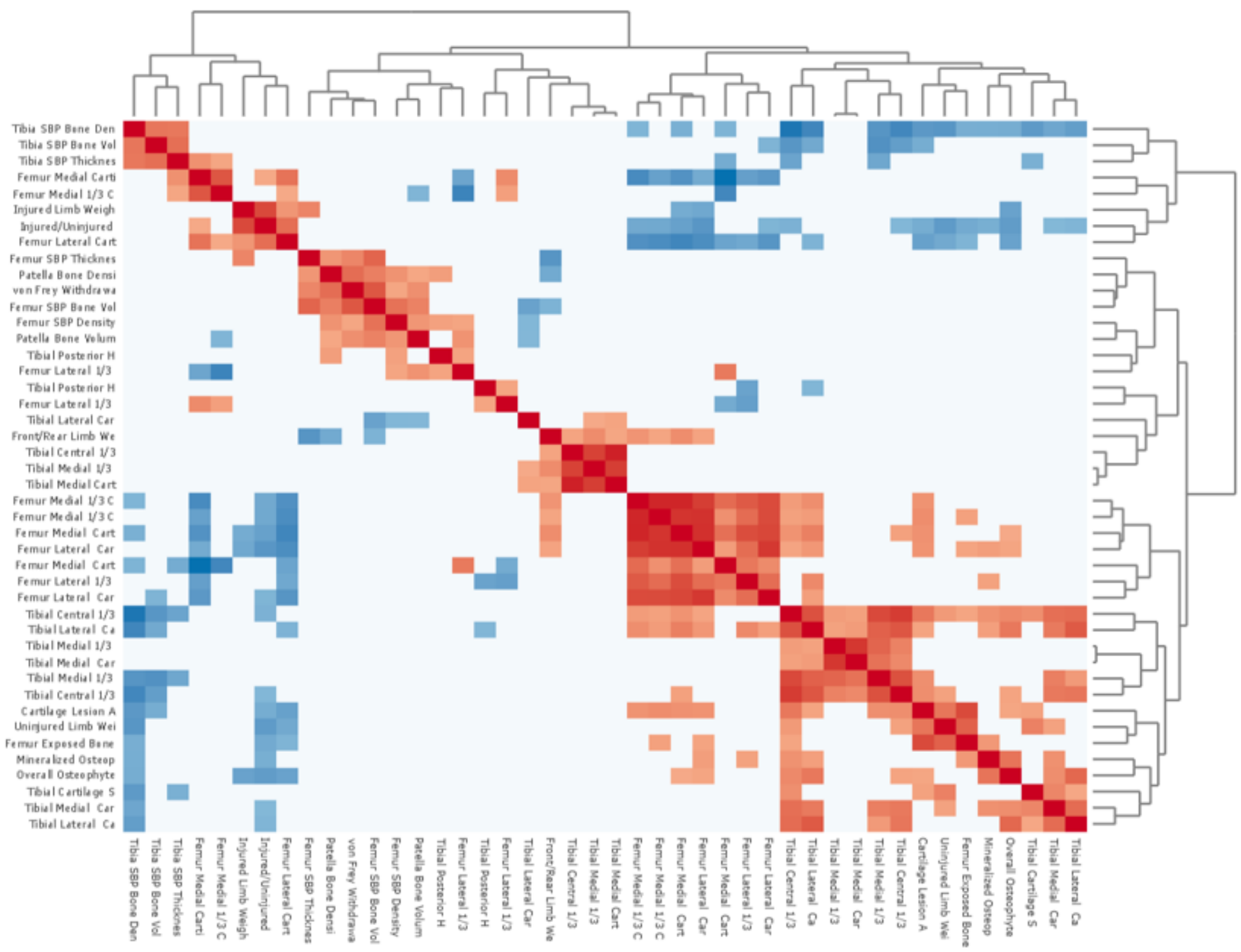

<u>Suppl. Figure 2A</u>: Spearman's Correlation Coefficient Heatmap was calculated for morphometric parameters to pain and functional metrics. Correlations are displayed for coefficient values (rho>0.5).

Correlation Analysis for morphometrics and pain behaviors

Features	correlation	p-value
von Frey Withdrawal	1	0
Patella Bone Density	0.82517	0.001719
Femur SBP Bone Volume	0.68531	0.017305
Femur SBP Thickness	0.66434	0.022159
Femur Medial 1/3 Cartilage Volume	0.65734	0.023981
Femur Medial 1/3 Cartilage Thickness	0.64336	0.027955
Tibial Cartilage Surface Roughness	0.58844	0.044145
Femur Medial Cartilage Thickness	0.54545	0.070679
Tibial Medial Cartilage Thickness	0.48252	0.11537
Femur Lateral Cartilage Attenuation	-0.46154	0.13384
Femur Lateral 1/3 Cartilage Attenuation	-0.45455	0.14041
Tibial Lateral Cartilage Thickness	0.44755	0.14721
Patella Bone Volume	0.43357	0.16145
Femur Lateral 1/3 Cartilage Thickness	0.42657	0.1689
Femur Medial Cartilage Volume	0.41958	0.17658
Femur Medial 1/3 Cartilage Attenuation	-0.41259	0.18448
Tibia SBP Density	-0.3986	0.20097
Tibial Central 1/3 Cartilage Thickness	0.39161	0.20956
Femur Medial Cartilage Attenuation	-0.38462	0.21839
Tibial Central 1/3 Cartilage Volume	0.37063	0.23673
Tibial Lateral Cartilage Attenuation	-0.36364	0.24626
Femur Lateral Cartilage Thickness	0.36364	0.24626
Tibial Lateral Cartilage Volume	0.34266	0.27623
Femur SBP Density	0.33566	0.28669
Cartilage Lesion Area	0.32867	0.29739
Tibial Medial 1/3 Cartilage Attenuation	-0.31469	0.31947
Femur Lateral Cartilage Volume	0.29371	0.35433
Tibia SBP Bone Volume	0.27671	0.38394
Femur Lateral 1/3 Cartilage Volume	0.27273	0.39123
Tibial Medial Cartilage Volume	0.23776	0.4571
Mineralized Osteophytes	0.16783	0.60373
Tibial Medial 1/3 Cartilage Volume	0.16783	0.60373
Tibial Posterior Horn Bone Density	0.16783	0.60373
Tibial Medial 1/3 Cartilage Thickness	0.11888	0.71618
Tibial Medial Cartilage Attenuation	-0.11888	0.71618
Femur Exposed Bone Area	0.11189	0.73278
Tibial Central 1/3 Cartilage Attenuation	-0.1049	0.74948
Tibia SBP Thickness	0.048951	0.88634
Overall Osteophytes	0.028021	0.93111
Tibial Posterior Horn Bone Volume	0.006993	0.99123

Features	correlation (rho)	p-value
Injured Limb Weight Bearing %	1	0
Femur SBP Bone Volume	0.89369	8.93E-05
Femur SBP Thickness	0.84686	0.00050996
Patella Bone Volume	0.69649	0.011853
Tibia SBP Density	-0.64005	0.024977
Patella Bone Density	0.61185	0.034485
Tibial Medial 1/3 Cartilage Attenuation	-0.5711	0.052438
Femur SBP Density	0.5443	0.067307
Cartilage Lesion Area	0.52847	0.077337
Tibial Central 1/3 Cartilage Thickness	0.48406	0.1108
Tibial Cartilage Surface Roughness	0.44847	0.14366
Femur Medial 1/3 Cartilage Volume	0.44605	0.1461
Femur Exposed Bone Area	0.44118	0.15109
Tibial Lateral Cartilage Attenuation	-0.39951	0.19821
Tibial Medial Cartilage Thickness	0.38049	0.22241
Femur Lateral 1/3 Cartilage Thickness	0.37385	0.23125
Tibial Central 1/3 Cartilage Attenuation	-0.37209	0.23364
Tibia SBP Bone Volume	-0.36238	0.24701
Tibia SBP Thickness	-0.35397	0.25897
Tibial Medial Cartilage Volume	0.34665	0.26965
Femur Medial Cartilage Volume	0.33216	0.2915
Femur Medial 1/3 Cartilage Attenuation	-0.32761	0.29856
Tibial Medial Cartilage Attenuation	-0.32284	0.30607
Femur Medial 1/3 Cartilage Thickness	0.30475	0.33547
Femur Medial Cartilage Thickness	0.30295	0.33848
Mineralized Osteophytes	0.29153	0.35789
Femur Lateral Cartilage Thickness	0.28973	0.361
Tibial Medial 1/3 Cartilage Thickness	0.28494	0.36935
Tibial Lateral Cartilage Thickness	0.25329	0.427
Tibial Central 1/3 Cartilage Volume	0.24479	0.44319
Femur Medial Cartilage Attenuation	-0.22212	0.48777
Femur Lateral 1/3 Cartilage Attenuation	-0.20442	0.52393
Tibial Medial 1/3 Cartilage Volume	0.20123	0.53057
Femur Lateral 1/3 Cartilage Volume	0.15613	0.62798
Tibial Posterior Horn Bone Density	0.12662	0.69496
Femur Lateral Cartilage Volume	0.11634	0.7188
Femur Lateral Cartilage Attenuation	-0.06137	0.84973
Tibial Lateral Cartilage Volume	0.058896	0.85573
Tibial Posterior Horn Bone Volume	0.055578	0.86379
Overall Osteophytes	0.0034599	0.99149

Suppl. Figure 2B: Correlation table comparing morphometric features with either pain sensitivity (von Frey) or left limb weight bearing function (DWB). Statistically meaningful ($p>0.05$) correlations with a correlation coefficient ($rho>0.5$) were further included as the main body text.

Supplemental Methods 1 - Animal Model and Study Design: Skeletally mature male Lewis rats (Charles River Laboratories, Wilmington, MA) were selected for their propensity to develop PTOA in previous ACL injury models[1–3] and surgical PTOA models[4–7]. Animals were co-housed with the same cagemate through the duration of the study with *ad libitum* food and water access in a temperature-controlled animal facility on a 12-hour light/dark cycle. All animal work was

conducted under the University of Oregon IACUC approved protocols (21-19). All animals were co-housed (2 animals/cage, the same paired animals through the duration of the study) using our standard housing procedures. Briefly, rats were housed in Tecniplast individually ventilated cage (Green Line GR900), with 1/8" pelleted cellulose bedding with Teklad Global 16% Protein Rodent Diet. Dietary enrichment consisted of BioServ Yogurt drops or Cheezitz (no more than 1 cracker/week). Water was acidified and sterilized, and then 6% sodium hyperchlorite was added to create a 5-10 ppm solution. Additional enrichment included NylaBones, PVC tubing, and Ancare Nestlets. Baseline pain-related behavioral measurements (weight bearing behavior, von Frey) were collected 1 day prior to procedure (Fig. 1A) and repeated 4-, 6-, and 8-weeks post-injury. Animals were euthanized at either 4-weeks (early mild PTOA phenotype) or 8-weeks (moderate-severe PTOA phenotype). Euthanasia occurred via CO2 asphyxiation with a secondary measure of cervical dislocation and confirmed by vital signs absent (VSA). Humane endpoints were considered a 10% drop in body weight or… but not needed in this study.

<u>Preliminary pilot study</u>: A non-survival pilot experiment (n=10) was initially conducted to characterize injury mode and location. Male Lewis rats (350 ± 9 g) were induced with 3-5% isoflurane and maintained under anesthesia via nose cone at 1.5-3% throughout the procedure. As previously described by Brown et al. 2020, animals were placed supine upon a custom machined platform and the ankle was tucked into a mounting pocket (20^{θ} pronation, 36^{θ} lateral rotation, 15^{θ} dorsiflexion). A 3D printed knee cup was lowered to snugly maintain contact with the knee in flexion[3]. Custom software[3] (Labview, National Instruments, Austin, TX) was used to control uniaxial loading transmitted through the knee cup via a pneumatic piston. A static preload (5 N) was first applied to the left hind limb and maintained for 30 seconds, followed by a loading ramp of 5 N/s until either the distal femur cranially translated out of the knee pocket (resulting

from release of the ACL after rupture) or a maximum load of 65 N was achieved, in which case the loading regimen was repeated for a maximum of two total repetitions. Translation of the tibia was confirmed immediately after loading in each animal via video recording and playback. Joint laxity and internal-external tibial rotation were assessed before and immediately after injury while animals were still anesthetized. Animals were immediately euthanized via CO_2 asphyxiation (primary) and cervical dislocation (secondary), imaged via X-ray (Faxitron, Hologic, Tucson, AZ) for potential bone fractures, and knee joints were dissected to confirm rupture. location, and identify and concomitant soft tissue damage or hematoma formation.

Joint laxity and rupture validation: Joint laxity was measured using a custom numeric scaling in three different planes of motion (anterior-posterior, medial-lateral, and cranial-caudal translation) and both internal and external joint rotation on a scale of 0-3 (0=baseline laxity immediately prior to injury, 1=moderately increased laxity, 2=high increased laxity, 3=completely free moving). Joint laxity in all three planes and rotational range of motion was summated for each animal (scored as a total 0-10) to determine a Physical Evaluation Score. Joints were carefully dissected with a tabletop dissection microscope to visually validate ACL injury and determine a Rupture Score (0=intact, 1=minor rupture or >50% of ACL intact, 2=partial rupture or >80% of ACL intact, 3=full rupture or 0% ACL intact) (A). Of the 10 animals in the preliminary pilot study, 70% exhibited an obvious complete ACL rupture (n=7/10, score=3), in 20% of cases pieces of the ACL fascia remained attached but the bulk ligament tissue was ruptured (n=2/10, score=2), and in 10% of animals the ACL was clearly necked and stretched to less than 50% of the originally ligament width (n=1/10, score=1). Physical Evaluation or Rupture Scores were linearly regressed against each other, failure load, or animal weights to correlate joint injury to injury modality. No significant relationships were determined (B). Failure location all according

in the proximal 1/3 of the distal ACL (towards the femoral insertion point). No bony fragments were identified within the knee joint (indicative of an avulsion from the femur/tibia).

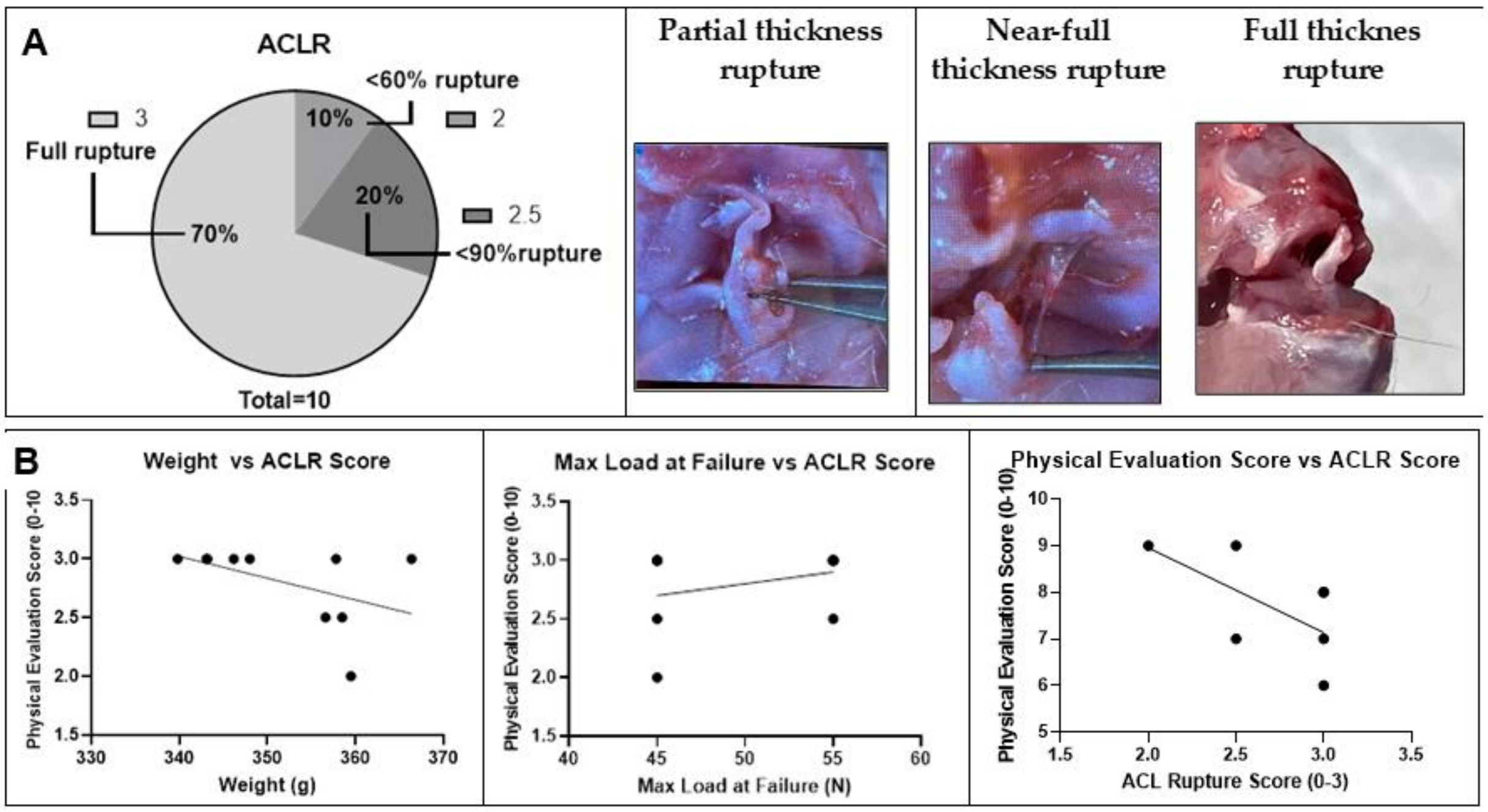

Suppl. Figure 3: (A) Joints were carefully dissected with a tabletop dissection microscope to visually validate ACL injury and determine a Rupture Score (0=intact, 1=minor rupture or >50% of ACL intact, 2=partial rupture or >80% of ACL intact, 3=full rupture or 0% ACL intact) (A). Of the 10 animals in the preliminary pilot study, 70% exhibited an obvious complete ACL rupture (n=7/10, score=3), in 20% of cases pieces of the ACL fascia remained attached but the bulk ligament tissue was ruptured (n=2/10, score=2), and in 10% of animals the ACL was clearly necked and stretched to less than 50% of the originally ligament width (n=1/10, score=1). (B) Physical Evaluation or Rupture Scores were linearly regressed against each other, failure load, or animal weights to correlate joint injury to injury modality. No significant relationships were determined.

Characterization study: Based upon the preliminary non-survival study, a sample size of n=10 for ACL rupture was selected (longitudinal measures: n=10/group for pain-related behaviors; post-mortem measures: n=7 computed tomography, n=3 histology) and n=7 for Sham (longitudinal measures: n=7/group for pain-related behaviors; post-mortem measures: n=6 computed tomography, n=1 histology). Sham only the 5 N static preload. Treatment groups were randomized immediately on the day of surgery, and Buprenorphine (0.5-1.5 mg/kg) was administered subcutaneously prior to injury as an analgesic agent. ACL injury was induced via the methods described above. Animals were monitored every 24 hrs through 5 days post-injury, and pain and grimace and gait scales used to monitor mentation and well-being, and repeated weekly. At 4-weeks, 10 ACL injury rats were randomly selected for euthanasia and tissues were harvested, and the remaining Sham and injury animals were euthanized at 8-weeks. ACLr was confirmed in all animals during tissue isolation immediately prior to CT imaging (ACL was no longer continuously attached to the femur). A 4-week Sham timepoint was not initially included in the study design but added in a subsequent study with age and weight matched male Lewis rats.

Supplemental Methods 2 – CT Morphometrics and Contouring: Evaluation scripts and contours were applied or adapted from previously established methods developed and rigorously characterized to be compatible with the medial meniscal transection Lewis rat model (same strain, age, and weights)[7–10]. Further, this set of cartilage and bone measurements are well recognized to correlate with histological and physical (e.g., needle probe testing) tissue assessments and have high reproducibility[7,10,11] . Consistent anatomical locations throughout the joint were used to ensure that the Volumes of Interest (VOI) for each evaluation parameter was consistently maintained across time and treatment. In cases where pathologies unique to the

ACLr (and distinct from MMT) where noted (e.g., degeneration of the tibial posterior medial horn), novel evaluation methods were developed and validated across samples.

Suppl. 2a-b - Tibial Morphometrics: For the cartilage evaluations, similarly structural morphologies were qualitatively observed between the ACLr limbs and previous MMT work[7–10], lead us to keep evaluation parameters similar. Briefly, tibial cartilage measurements began in the medial tibial compartment in the sagittal plane(A), when the posterior load bearing surface was 1.5 mm from divot ($) to peak (&). The VOI was expanded to follow the divot as it shifted more posterior throughout the medial edge of the medial plateau (B), using the anterior divot as one contouring bound and the most posterior aspect of the medial horn (#) as the posterior bound, and continued until the 1.5 mm measurement returned on the leading medial edge. The total number of slices was divided into thirds to isolate a "medial", "central", and "lateral" aspect of the medial plateau for evaluation. The same evaluation bounds were mirrored for analysis of the lateral plateau. Cartilage 3D reconstructions are shown as a heat map of cartilage thickness (relative to .250 mm thickness) and overlayed with the entire mineralized joint components (C) For the posterior Horn Volume, the same range of slices identified in the medial cartilage evaluations was modified to add a rectangular VOI starting at the most posterior aspect of the medial horn (#) as the posterior bound and expanding 2 mm anteriorly (D). A custom component labeling and greyscale masking process was used to evaluate the largest interconnected bone piece (the posterior horn) and separate growth plate or unconnected trabecular structures. Bone total mineral density, volume, and deviation was evaluated in the component labelled posterior horn throughout the medial slice range.

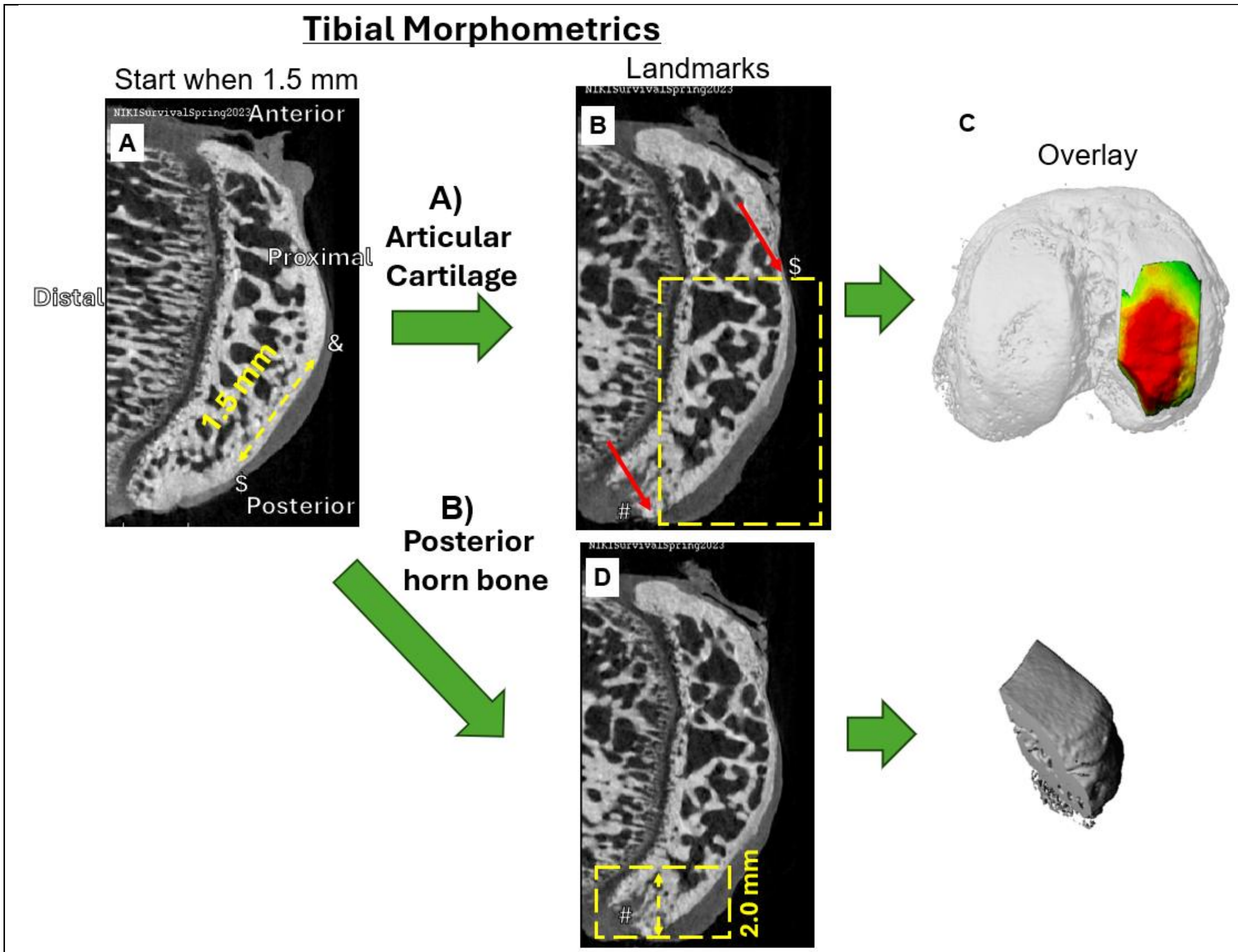

<u>Suppl. Figure 4A:</u> (A) Cartilage measurements were calculated in the sagittal view of the medial tibial plateau. Briefly, tibial cartilage measurements began in the medial tibial compartment in the sagittal plane (A), when the posterior load bearing surface was 1.5 mm from divot ($) to peak (&). The VOI was expanded to follow the divot as it shifted more posterior throughout the medial edge of the medial plateau (B), using the anterior divot as one contouring bound and the most posterior aspect of the medial horn (#) as the posterior bound, and continued until the 1.5 mm measurement returned on the leading medial edge. (B) Tibial medial posterior horn bone volume was calculated in the posterior aspect of the tibia using the same range and bounds as the cartilage measurements.

Suppl. 2c - Femoral morphometrics: For the cartilage evaluations, femora were carefully qualitatively compared using 2D sagittal planes and histology to identify consistent medial and lateral compartmental anatomical landmarks regardless of treatment for contouring and evaluation. Starting when the distal-proximal distance of the posterior medial femoral compartment in the sagittal plane was 1.5 mm long (A), a rectangular VOI was used to measure cartilage parameters from the anterior femoral divot ($) to the most posterior aspect of the condyle (#) (B). Contours were continued through the medial condyle, with the VOI expanding to stay withing the identified boundaries. Measurements were finished 2 slices after either a ligament insertion point (red circle) (C) or the medial cortical bone became observable (blue circle). The same evaluation bounds were mirrored for analysis of the lateral plateau. For the femoral subchondral plate, he same range of slices and bounds in the sagittal plane were used from the cartilage analysis, but the subchondral plate was manually contoured away from the trabecular bone within the bounds between the anterior femoral divot ($) to the most posterior aspect of the condyle (#) (E). Contouring continued throughout the condyle and ended at the same slice as the cartilage measurements.

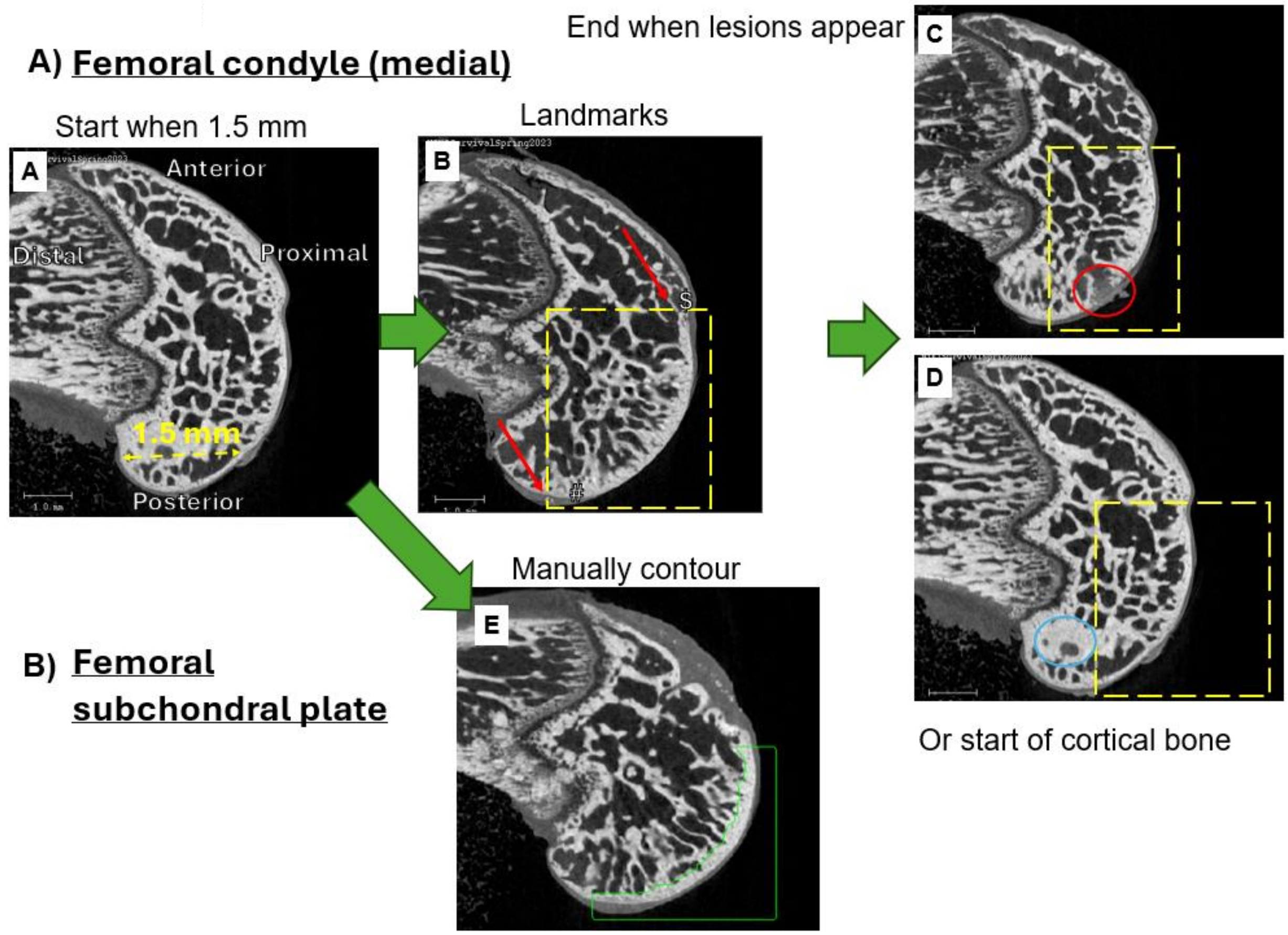

Suppl. Figure 4B-C: (A) Cartilage measurements were calculated in the sagittal view of the medial femoral condyle, ending when either the medial ligamentous insertion or cortical bone appear. Starting when the distal-proximal distance of the posterior medial femoral compartment in the sagittal plane was 1.5 mm long (A), a rectangular VOI was used to measure cartilage parameters from the anterior femoral divot ($) to the most posterior aspect of the condyle (#).Measurements were finished 2 slices after either a ligament insertion point (red circle) (C) or the medial cortical bone became observable (blue circle). The same evaluation bounds were mirrored for analysis of the lateral plateau. (B) Femoral subchondral plate measurements were calculated over the same range and bounds as the cartilage measures but with manual contouring of the subchondral plate.

Suppl. 2d - Cartilage surface roughness and lesion area: Serial sagittal sections of femora and tibiae were exported as 2D grayscale TIFF images throughout the bounds identified in the cartilage morphometric analysis and processed with a previously developed custom MATLAB R2023a (MathWorks, Natick, MA) algorithm to calculate cartilaginous surface roughness, exposed bone area, lesion volume, and lesion area[9] (A,B). Briefly, cartilage regions of interest were fitted with a 3D polynomial surface (quadratic-quartic) (C) and the root mean square error of the deviation between the fitted surface and articular cartilage surface was defined as the roughness (Sq) of the articular cartilage surface (D). Exposed bone, lesion area and lesion volume were calculated via a pixel thresholding intensity between the cartilage and bone interface of the femoral condyle or tibial plateau. A relative proximity measure was used to define exposed bone regions, where a proximity of 3 or fewer pixels between a pixel thresholded as cartilage and one thresholded as bone was considered a region of fully eroded cartilage. Lesion volume was defined as regions experiencing greater than 50% cartilage thickness loss, and lesion area was defined as the surface area of the eroded regions experiencing less than 50% loss of cartilage thickness.

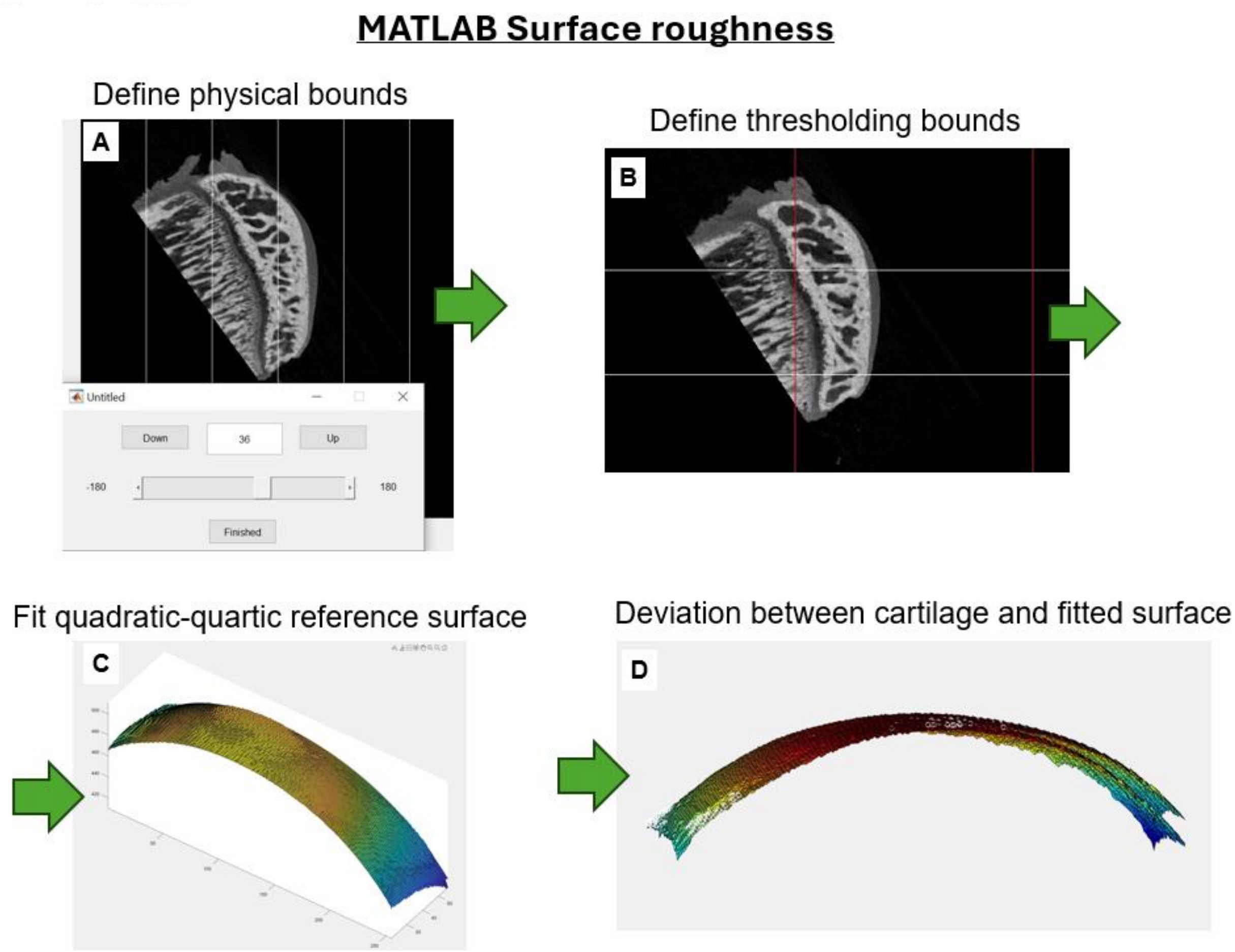

Suppl. Figure 4D: (A) Imported TIF files of the medial plateau or cartilage were aligned and (B) physical bounds marked to ensure cartilage and bone were present in all analyzed TIF slices. Once these images were thresholded for cartilage and bone, (C) a polynomial quadratic-quartic reference cartilage surface was fitted for each sample, and then (D) the deviation between the fitted surface and thresholded cartilage surface was used to calculate the surface roughness measurements.

Suppl. 2e - Tibial mineralized and cartilaginous osteophytes: Osteophytes along the leading medial periphery of the medial tibia plateau were manually contoured in the coronal plane as previously described in detail[12]. Starting when the flat plateau of the medial tibial

plateau was 1.5 mm across (A) and between the intercondylar inflection point (&) and medial divot ($), marginal tissues were manually contoured (B) to include peripheral cartilaginous or mineralized components (example is a Sham treatment) from the peripheral until the medial divot ($), with dissection tissue artefact carefully excluded (C) (red arrow). Contours continued until the plateau returned to 1.5 mm in length from the inflection point to divot (D). A cartilaginous and mineralized component were thresholded based upon density to quantify volume and added for a total osteophyte volume. For the tibial subchondral plate, the same range of slices and bounds in the coronal plane were used from the osteophyte analysis, but the subchondral plate was manually contoured away from the trabecular bone within the bounds between the intercondylar inflection point (&) and medial divot ($) (A). Contouring continued throughout the slice range, with careful recording of pathological bone pores (red arrow) (B) and bone density gradients (blue arrow) (C) (example is an ACLr treatment). Contouring ended at the same slice as the osteophyte measure.

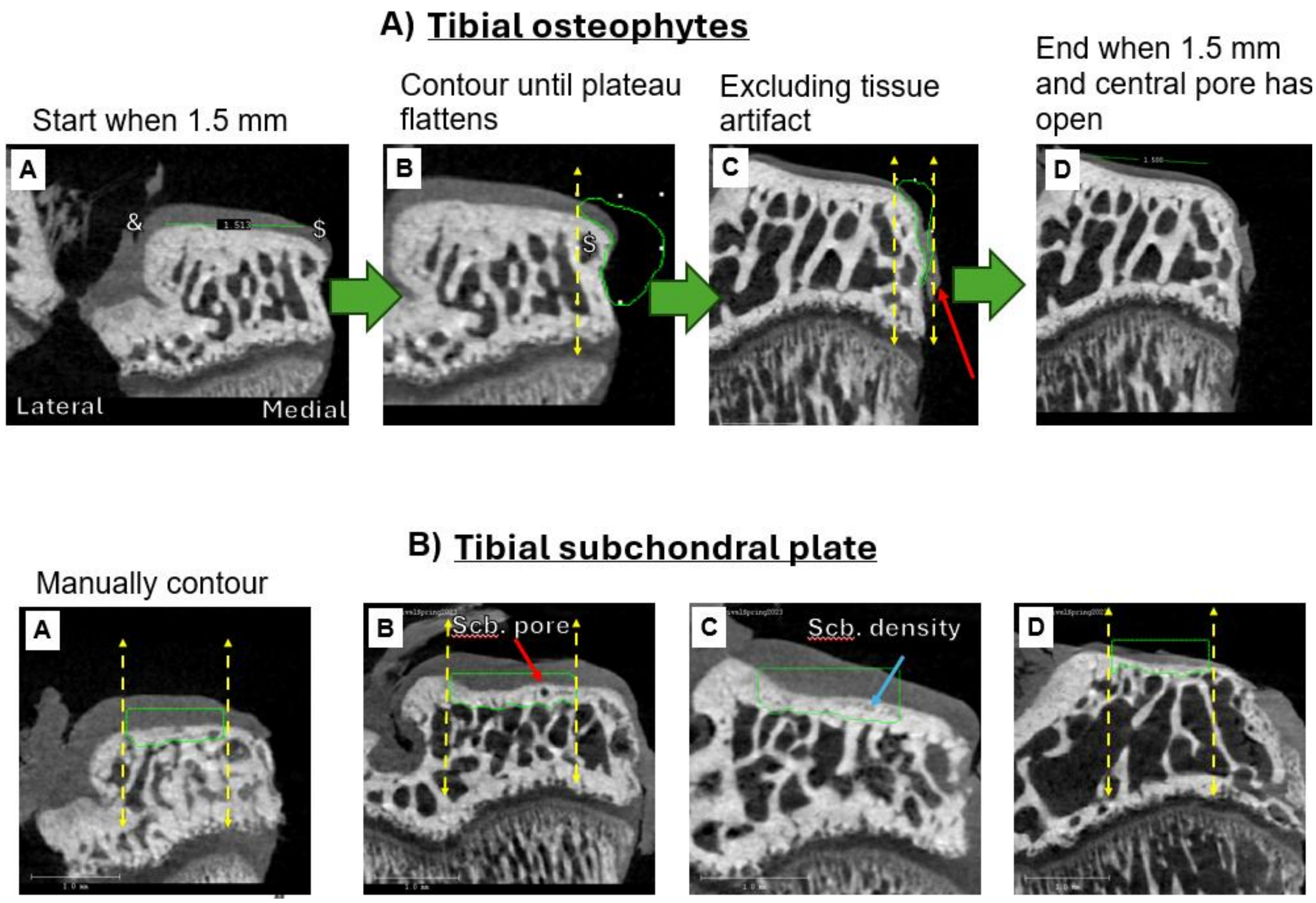

Suppl. Figure 4E: (A) Peripheral tibial osteophytes were manually contoured in the coronal plane from the anterior towards the posterior aspect. Starting when the flat plateau of the medial tibial plateau was 1.5 mm across (A) and between the intercondylar inflection point (&) and medial divot ($), marginal tissues were manually contoured (B) to include peripheral cartilaginous or mineralized components (example is a Sham treatment) from the peripheral until the medial divot ($), with dissection tissue artefact carefully excluded (C) (red arrow). Contours continued until the plateau returned to 1.5 mm in length from the inflection point to divot (D). (B) Tibial subchondral plate was manually contoured across the same range and bounds as the osteophyte measurements, with special attention to subchondral plate bone pores (red arrow) and regions of visible changes in local plate mineral density (blue arrow).

Supplemental Methods 3 – Sagittal and Coronal Histology: Knee joint tissues (n=4/ACLr group, n=1 Sham) were dissected immediately post-mortem and fixed in 10% formalin for at least two weeks, with the solution replaced after three days. Whole joints were decalcified in Immunocal (10% formic acid), trimmed in half in the frontal plane, embedded in paraffin, and sectioned either in coronal or sagittal planes (Inotiv, Boulder, CO). Each coronal block was sectioned into 5 um slices at 200 steps in four levels throughout the joint (one at central, one anterior to central, two posteriors to central) to generate 3 slices per level. Each sagittal block was sectioned into 5 um slices at 200 steps in five levels throughout the joint to capture the anterior cruciate, posterior cruciate, or anterior and posterior cruciate ligaments in each level to generate 3 slices per level. In both cases, slides at each level were stained with hematoxylin & eosin (H&E), safranin-O Fast Green (Saf-O/FG) or left unstained. Whole slides were imaged on a Leica Aperio Versa 200 Slide Scanner with 10X magnification (Leica Biosystems, Deer Park, IL) at the University of Oregon.

***Supplemental Methods 4 – Advanced Biostatistics*:** Sparse Partial Least Squares-Discriminant Analysis (sPLS-DA) was selected as a supervised dimensionality reduction technique to support inferential statistics regarding key pathologies after ACL rupture. Techniques were conducted using the established Metaboanalyst pathway (Metaboanalyst 6.0, Montreal, QC, Canada)[26] for sPLS-DA (feature size of 10, top 5 principle components).

Suppl. 4a – Calculating sPLS-DA error and model ensemble: The generated model was assessed using Leave-One-Out Cross Validation (LOOCV) rather than a k-fold validation method (e.g., 10-fold cross validation) because of the relatively small sample data set. Although the authors acknowledge LOOCV results in a potentially higher model variance with a reduced bias. With this method Error rates for Components 1 and 2 were 22%, while subsequent

components 3-5 had a similar variance of 18% (Fig. 4a). A pairwise ensemble of each component score plot highlights that Component 1 (33%) effectively separates ACLr from Sham controls when paired with all components (Fig. 4a).

Suppl. 4b – Loading Plot for Components 1-3 and Synchronized 3-Dimensionality: Component 1 drove separation between OA and Sham control groups with tibial SBP density, and medial femoral cartilage degeneration as determining features (Fig. 4b). 4-week (mild OA) and 8-week (severe OA) ACLr separation was clear along Component 2 with changes to femur SBP bone density and volume, pain sensitization, and patella degeneration serving as determining features. A synchronized 3D Score Plot summarizes separation between groups with the first 3 principal components.

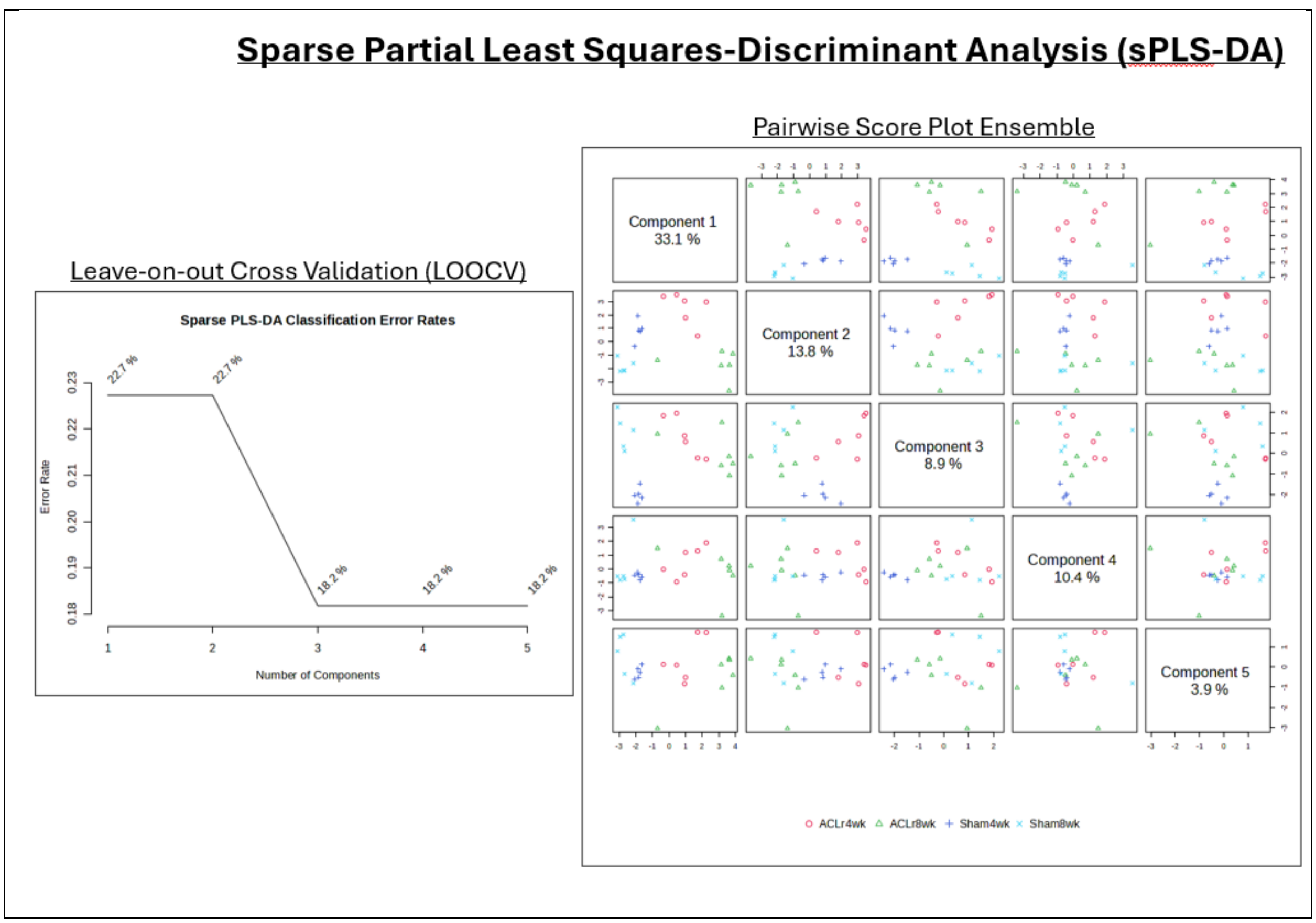

<u>Suppl. Figure 5A:</u> Sparse PLS-DA error was calculated with Leave-one-out Cross Validation for the first 5 components. A model ensembles consisting of the first 5 components highlights the relationship between the different features and groups.

Suppl. 4b – Loading Plot for Components 1-3 and Synchronized 3-Dimensionality:

Component 1 drove separation between OA and Sham control groups with tibial SBP density, and medial femoral cartilage degeneration as determining features (Fig. 4b). 4-week (mild OA) and 8-week (severe OA) ACLr separation was clear along Component 2 with changes to femur SBP bone density and volume, pain sensitization, and patella degeneration serving as determining features. A synchronized 3D Score Plot summarizes separation between groups with the first 3 principal components.

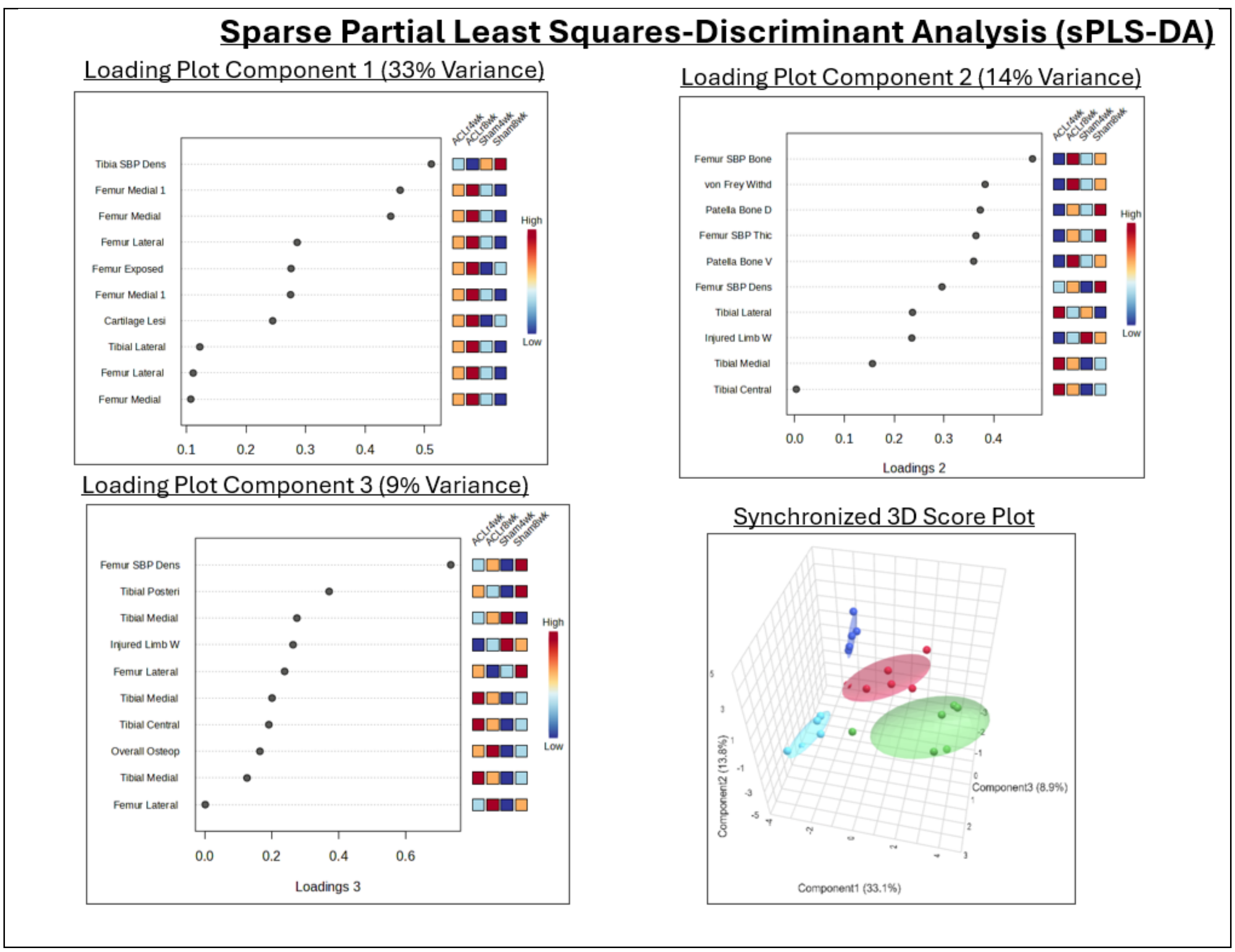

<u>Suppl. Figure 5B:</u> Sparse PLS-DA Component plots for the first 3 components, capturing 9-33% of total variance, with the associated 10 features in each component. A synchronized 3D score plot demonstrates strong separation of injury and sham and each timepoint along the first 3 components.

***Supplemental Methods 5 – Correlating pathology and pain*:** An exploratory Spearman's Correlation heatmap was first generated for cartilage and bone pathologies with weight bearing and von Frey pain related behaviors. Spearman's Rank Correlation was selected to negate the assumption that all relationships would be linear and normally distributed (given that multiple tissue parameters were identified with descriptive statistics as having non-Gaussian distributions). Associations were then filtered as greater than 0.5 (e.g., a mild monotonic correlation) (Fig. 5a).

Suppl. 5b – Correlation tables and summary description: Relationships identified in the correlation heatmaps were assessed for statistical significance (set as $p<0.05$) to identify statistically meaningful correlations with limb weight bearing function and pain sensitivity. Coefficients up to +1 represent strong positive monotonic relationships while coefficients down to -1 represent strong negative monotonic relationships. Pathologies with strong (0.55-0.75) and very-strong (0.75-0.99) correlations that were statistically significant were individually plotted to describe associations in the main body text. For example, patella bone density *vs* Von Frey or Femur SBP Volume *vs* weight bearing.

Supplemental References

1. Maerz, T. *et al.* Articular cartilage degeneration following anterior cruciate ligament injury: a comparison of surgical transection and noninvasive rupture as preclinical models of post-traumatic osteoarthritis. *Osteoarthritis Cartilage* **24**, 1918–1927 (2016).
2. Maerz, T. *et al.* Subchondral and epiphyseal bone remodeling following surgical transection and noninvasive rupture of the anterior cruciate ligament as models of post-traumatic osteoarthritis. *Osteoarthritis Cartilage* **24**, 698–708 (2016).
3. Brown, S. B. *et al.* Characterization of Post-Traumatic Osteoarthritis in Rats Following Anterior Cruciate Ligament Rupture by Non-Invasive Knee Injury (NIKI). *J. Orthop. Res. Off. Publ. Orthop. Res. Soc.* **38**, 356–367 (2020).
4. Bove, S. E. *et al.* Surgically induced osteoarthritis in the rat results in the development of both osteoarthritis-like joint pain and secondary hyperalgesia. *Osteoarthritis Cartilage* **14**, 1041–1048 (2006).
5. Thote, T. *et al.* Localized 3D analysis of cartilage composition and morphology in small animal models of joint degeneration. *Osteoarthritis Cartilage* **21**, 1132–1141 (2013).
6. Kaiser, J. M. *et al.* Mild Exercise Expedites Joint Clearance and Slows Joint Degradation in a Joint Instability Model of Osteoarthritis in Male Rats. *Osteoarthritis Cartilage* (2024) doi:10.1016/j.joca.2024.03.120.
7. Xie, L., Lin, A. S. P., Levenston, M. E. & Guldberg, R. E. Quantitative Assessment of Articular Cartilage Morphology via EPIC-μCT. *Osteoarthr. Cartil. OARS Osteoarthr. Res. Soc.* **17**, 313–320 (2009).
8. Mancipe Castro, L. M., Sequeira, A., García, A. J. & Guldberg, R. E. Articular cartilage- and synoviocyte-binding poly(ethylene glycol) nano-composite microgels as intra-articular drug

delivery vehicles for the treatment of osteoarthritis. *ACS Biomater. Sci. Eng.* **6**, 5084–5095 (2020).

9. Reece, D. S., Thote, T., Lin, A. S. P., Willett, N. J. & Guldberg, R. E. Contrast enhanced μCT imaging of early articular changes in a pre-clinical model of osteoarthritis. *Osteoarthritis Cartilage* **26**, 118–127 (2018).
10. Palmer, A. W., Guldberg, R. E. & Levenston, M. E. Analysis of cartilage matrix fixed charge density and three-dimensional morphology via contrast-enhanced microcomputed tomography. *Proc. Natl. Acad. Sci. U. S. A.* **103**, 19255–19260 (2006).
11. Xie, L., Lin, A. S. P., Guldberg, R. E. & Levenston, M. E. Nondestructive assessment of sGAG content and distribution in normal and degraded rat articular cartilage via EPIC-μCT. *Osteoarthritis Cartilage* **18**, 65–72 (2010).
12. Willett, N. J. *et al.* Quantitative pre-clinical screening of therapeutics for joint diseases using contrast enhanced micro-computed tomography. *Osteoarthritis Cartilage* **24**, 1604–1612 (2016).

SPONTANEOUS AND EVOKED PAIN-RELATED BEHAVIORS

Von Frey (tactile allodynia) – mixed effects analysis

Tukey's multiple comparisons test	Mean Diff.	95.00% CI of diff.	Summary	Adjusted P Value
Baseline				
Sham (L) vs. NIKI (L)	0.000			
Sham (L) vs. NIKI Contralateral (R)	0.000			
NIKI (L) vs. NIKI Contralateral (R)	0.000			
4 wk				
Sham (L) vs. NIKI (L)	0.3642	0.04080 to 0.6876	*	0.0293
Sham (L) vs. NIKI Contralateral (R)	-0.04473	-0.3733 to 0.2839	ns	0.9263
NIKI (L) vs. NIKI Contralateral (R)	-0.4090	-0.5874 to -0.2305	****	<0.0001
6 wk				
Sham (L) vs. NIKI (L)	0.3465	-0.0009768 to 0.6939	ns	0.0506
Sham (L) vs. NIKI Contralateral (R)	-0.2563	-0.6813 to 0.1688	ns	0.2869
NIKI (L) vs. NIKI Contralateral (R)	-0.6027	-0.9619 to -0.2435	**	0.0020
8 wk				
Sham (L) vs. NIKI (L)	0.1051	-0.4523 to 0.6625	ns	0.8696
Sham (L) vs. NIKI Contralateral (R)	0.2248	-0.3046 to 0.7542	ns	0.4985
NIKI (L) vs. NIKI Contralateral (R)	0.1197	-0.2879 to 0.5273	ns	0.7279
Sham (L)				
Baseline vs. 4 wk	-0.002240	-0.3653 to 0.3608	ns	>0.9999
Baseline vs. 6 wk	0.002338	-0.3778 to 0.3825	ns	>0.9999
Baseline vs. 8 wk	-0.1391	-0.7178 to 0.4396	ns	0.8378
4 wk vs. 6 wk	0.004579	-0.5954 to 0.6046	ns	>0.9999
4 wk vs. 8 wk	-0.1369	-1.024 to 0.7507	ns	0.9476
6 wk vs. 8 wk	-0.1414	-0.6279 to 0.3450	ns	0.7520
NIKI (L)				
Baseline vs. 4 wk	0.3620	0.2352 to 0.4887	****	<0.0001
Baseline vs. 6 wk	0.3488	0.1503 to 0.5473	**	0.0022
Baseline vs. 8 wk	-0.03400	-0.4403 to 0.3723	ns	0.9919
4 wk vs. 6 wk	0.05873	-0.2417 to 0.3592	ns	0.9208
4 wk vs. 8 wk	-0.3016	-0.9406 to 0.3374	ns	0.4543
6 wk vs. 8 wk	-0.4076	-0.9712 to 0.1560	ns	0.1657
NIKI Contralateral (R)				
Baseline vs. 4 wk	-0.04697	-0.2092 to 0.1152	ns	0.8450
Baseline vs. 6 wk	-0.2539	-0.6367 to 0.1289	ns	0.2245
Baseline vs. 8 wk	0.1653	-0.02724 to 0.3577	ns	0.0948

4 wk vs. 6 wk	-0.7682 to 0.2232	No	ns	0.3565
4 wk vs. 8 wk	-0.1908 to 0.4842	No	ns	0.5372
6 wk vs. 8 wk	0.1331 to 0.7053	Yes	**	0.0068

Test details	Mean 2	Mean Diff.	SE of diff.	N1	N2
Baseline					
Sham (L) vs. NIKI (L)	1.000	0.000	0.000	7	19
Sham (L) vs. NIKI Contralateral (R)	1.000	0.000	0.000	7	19
NIKI (L) vs. NIKI Contralateral (R)	1.000	0.000	0.000	19	19
4 wk					
Sham (L) vs. NIKI (L)	0.6380	0.3642	0.1141	7	19
Sham (L) vs. NIKI Contralateral (R)	1.047	-0.04473	0.1195	7	19
NIKI (L) vs. NIKI Contralateral (R)	1.047	-0.4090	0.07284	19	19
6 wk					
Sham (L) vs. NIKI (L)	0.6512	0.3465	0.1261	7	9
Sham (L) vs. NIKI Contralateral (R)	1.254	-0.2563	0.1623	7	9
NIKI (L) vs. NIKI Contralateral (R)	1.254	-0.6027	0.1347	9	9
8 wk					
Sham (L) vs. NIKI (L)	1.034	0.1051	0.2074	7	8
Sham (L) vs. NIKI Contralateral (R)	0.9143	0.2248	0.1928	7	10
NIKI (L) vs. NIKI Contralateral (R)	0.9143	0.1197	0.1558	8	10
Sham (L)					
Baseline vs. 4 wk	1.002	-0.002240	0.1049	7	7
Baseline vs. 6 wk	0.9977	0.002338	0.1098	7	7
Baseline vs. 8 wk	1.139	-0.1391	0.1672	7	7
4 wk vs. 6 wk	0.9977	0.004579	0.1733	7	7
4 wk vs. 8 wk	1.139	-0.1369	0.2564	7	7
6 wk vs. 8 wk	1.139	-0.1414	0.1405	7	7
NIKI (L)					
Baseline vs. 4 wk	0.6380	0.3620	0.04484	19	19
Baseline vs. 6 wk	0.6512	0.3488	0.06198	9	9
Baseline vs. 8 wk	1.034	-0.03400	0.1228	8	8
4 wk vs. 6 wk	0.6512	0.05873	0.09382	9	9
4 wk vs. 8 wk	1.034	-0.3016	0.1931	8	8
6 wk vs. 8 wk	1.034	-0.4076	0.1703	8	8
NIKI Contralateral (R)					
Baseline vs. 4 wk	1.047	-0.04697	0.05739	19	19
Baseline vs. 6 wk	1.254	-0.2539	0.1195	9	9
Baseline vs. 8 wk	0.8347	0.1653	0.06011	9	9
4 wk vs. 6 wk	1.254	-0.2725	0.1548	9	9
4 wk vs. 8 wk	0.8347	0.1467	0.1054	9	9

6 wk vs. 8 wk	0.8347	0.4192	0.08934	9		9

Injured Hindlimb Weight Bearing – mixed effects analysis

Bonferroni's multiple	Predicted (LS) mean diff.	95.00% CI of diff.	Below threshold?	Summary	Adjusted P Value	
Baseline						
Sham vs. NIKI	0.000	-0.08269 to 0.08269	No	ns	>0.9999	
4 wk						
Sham vs. NIKI	0.4162	0.3329 to 0.4994	Yes	****	<0.0001	
6 wk						
Sham vs. NIKI	0.3306	0.2381 to 0.4232	Yes	****	<0.0001	
8 wk						
Sham vs. NIKI	0.2368	0.1443 to 0.3293	Yes	****	<0.0001	
Sham						
Baseline vs. 4 wk	0.09890	-0.03042 to 0.2282	No	ns	0.2454	
Baseline vs. 6 wk	0.03121	-0.09811 to 0.1605	No	ns	>0.9999	
Baseline vs. 8 wk	-0.03514	-0.1645 to 0.09418	No	ns	>0.9999	
4 wk vs. 6 wk	-0.06769	-0.1970 to 0.06163	No	ns	0.9447	
4 wk vs. 8 wk	-0.1340	-0.2634 to -0.004723	Yes	*	0.0382	
6 wk vs. 8 wk	-0.06635	-0.1957 to 0.06297	No	ns	0.9938	
NIKI						
Baseline vs. 4 wk	0.5151	0.4374 to 0.5927	Yes	****	<0.0001	
Baseline vs. 6 wk	0.3619	0.2664 to 0.4573	Yes	****	<0.0001	
Baseline vs. 8 wk	0.2016	0.1062 to 0.2971	Yes	****	<0.0001	
4 wk vs. 6 wk	-0.1532	-0.2494 to -0.05703	Yes	***	0.0003	
4 wk vs. 8 wk	-0.3134	-0.4096 to -0.2173	Yes	****	<0.0001	
6 wk vs. 8 wk	-0.1602	-0.2684 to -0.05203	Yes	***	0.0010	
Test details	Predicted (LS) mean 1	Predicted (LS) mean 2	Predicted (LS) mean diff.	SE of diff.	N1	N2
Sham						
Baseline vs. 4 wk	1.000	0.9011	0.09890	0.04722	7	7
Baseline vs. 6 wk	1.000	0.9688	0.03121	0.04722	7	7
Baseline vs. 8 wk	1.000	1.035	-0.03514	0.04722	7	7
4 wk vs. 6 wk	0.9011	0.9688	-0.06769	0.04722	7	7
4 wk vs. 8 wk	0.9011	1.035	-0.1340	0.04722	7	7
6 wk vs. 8 wk	0.9688	1.035	-0.06635	0.04722	7	7
NIKI						
Baseline vs. 4 wk	1.000	0.4849	0.5151	0.02834	20	19
Baseline vs. 6 wk	1.000	0.6381	0.3619	0.03486	20	10
Baseline vs. 8 wk	1.000	0.7984	0.2016	0.03486	20	10
4 wk vs. 6 wk	0.4849	0.6381	-0.1532	0.03511	19	10
4 wk vs. 8 wk	0.4849	0.7984	-0.3134	0.03511	19	10

6 wk vs. 8 wk	0.6381	0.7984	-0.1602	0.03950	10	10

Uninjured Hindlimb Weight Bearing – mixed effects analysis

Bonferroni's multiple	Predicted (LS) mean diff.	95.00% CI of diff.	Below threshold?	Summary	Adjusted P Value
Sham					
Baseline vs. 4 wk	0.05336	-0.1080 to 0.2147	No	ns	>0.9999
Baseline vs. 6 wk	-0.06921	-0.2305 to 0.09212	No	ns	>0.9999
Baseline vs. 8 wk	-0.1761	-0.3374 to -0.01475	Yes	*	0.0252
4 wk vs. 6 wk	-0.1226	-0.2839 to 0.03876	No	ns	0.2532
4 wk vs. 8 wk	-0.2294	-0.3908 to -0.06811	Yes	**	0.0016
6 wk vs. 8 wk	-0.1069	-0.2682 to 0.05446	No	ns	0.4511
NIKI					
Baseline vs. 4 wk	-0.2294	-0.3264 to -0.1325	Yes	****	<0.0001
Baseline vs. 6 wk	-0.2837	-0.4037 to -0.1637	Yes	****	<0.0001
Baseline vs. 8 wk	-0.4439	-0.5639 to -0.3239	Yes	****	<0.0001
4 wk vs. 6 wk	-0.05428	-0.1751 to 0.06652	No	ns	>0.9999
4 wk vs. 8 wk	-0.2145	-0.3353 to -0.09368	Yes	****	<0.0001
6 wk vs. 8 wk	-0.1602	-0.2952 to -0.02522	Yes	*	0.0119

Test details	Predicted (LS) mean 1	Predicted (LS) mean 2	Predicted (LS) mean diff.	SE of diff.	N1	N2
Sham						
Baseline vs. 4 wk	1.000	0.9466	0.05336	0.05890	7	7
Baseline vs. 6 wk	1.000	1.069	-0.06921	0.05890	7	7
Baseline vs. 8 wk	1.000	1.176	-0.1761	0.05890	7	7
4 wk vs. 6 wk	0.9466	1.069	-0.1226	0.05890	7	7
4 wk vs. 8 wk	0.9466	1.176	-0.2294	0.05890	7	7
6 wk vs. 8 wk	1.069	1.176	-0.1069	0.05890	7	7
NIKI						
Baseline vs. 4 wk	1.000	1.229	-0.2294	0.03539	20	19
Baseline vs. 6 wk	1.000	1.284	-0.2837	0.04381	20	10
Baseline vs. 8 wk	1.000	1.444	-0.4439	0.04381	20	10
4 wk vs. 6 wk	1.229	1.284	-0.05428	0.04410	19	10
4 wk vs. 8 wk	1.229	1.444	-0.2145	0.04410	19	10
6 wk vs. 8 wk	1.284	1.444	-0.1602	0.04928	10	10

Front/Rear Limb Weight Bearing Ratio – mixed effects analysis

Bonferroni's multiple	Predicted (LS) mean diff.	95.00% CI of diff.	Below threshold?	Summary	Adjusted P Value
Sham					
Baseline vs. 4 wk	-0.01236	-0.2718 to 0.2471	No	ns	>0.9999

Baseline vs. 6 wk	0.04879	-0.2107 to 0.3083	No	ns	>0.9999
Baseline vs. 8 wk	0.1128	-0.1467 to 0.3722	No	ns	>0.9999
4 wk vs. 6 wk	0.06115	-0.1983 to 0.3206	No	ns	>0.9999
4 wk vs. 8 wk	0.1251	-0.1344 to 0.3846	No	ns	>0.9999
6 wk vs. 8 wk	0.06397	-0.1955 to 0.3235	No	ns	>0.9999
NIKI					
Baseline vs. 4 wk	-0.2685	-0.4220 to -0.1150	Yes	****	<0.0001
Baseline vs. 6 wk	-0.1395	-0.3298 to 0.05082	No	ns	0.2986
Baseline vs. 8 wk	-0.02459	-0.2149 to 0.1657	No	ns	>0.9999
4 wk vs. 6 wk	0.1290	-0.06131 to 0.3194	No	ns	0.4134
4 wk vs. 8 wk	0.2439	0.05361 to 0.4343	Yes	**	0.0054
6 wk vs. 8 wk	0.1149	-0.1022 to 0.3320	No	ns	0.9184

Test details	Predicted (LS) mean 1	Predicted (LS) mean 2	Predicted (LS) mean diff.	SE of diff.	N1	N2
Sham						
Baseline vs. 4 wk	1.000	1.012	-0.01236	0.09481	7	7
Baseline vs. 6 wk	1.000	0.9512	0.04879	0.09481	7	7
Baseline vs. 8 wk	1.000	0.8872	0.1128	0.09481	7	7
4 wk vs. 6 wk	1.012	0.9512	0.06115	0.09481	7	7
4 wk vs. 8 wk	1.012	0.8872	0.1251	0.09481	7	7
6 wk vs. 8 wk	0.9512	0.8872	0.06397	0.09481	7	7
NIKI						
Baseline vs. 4 wk	1.000	1.269	-0.2685	0.05609	20	20
Baseline vs. 6 wk	1.000	1.140	-0.1395	0.06954	20	10
Baseline vs. 8 wk	1.000	1.025	-0.02459	0.06954	20	10
4 wk vs. 6 wk	1.269	1.140	0.1290	0.06954	20	10
4 wk vs. 8 wk	1.269	1.025	0.2439	0.06954	20	10
6 wk vs. 8 wk	1.140	1.025	0.1149	0.07932	10	10

FEMORAL ARTICULAR CARTILAGE MORPHOMETRICS

Medial Cartilage Thickness:

Dunn's multiple comparisons test	Mean rank diff.	Significant?	Summary	Adjusted P Value	
Sham (4 wk) vs. Sham (8 wk)	5.800	No	ns	0.9472	A-B
Sham (4 wk) vs. ACLr (4 wk)	-5.833	No	ns	0.8276	A-C
Sham (4 wk) vs. ACLr (8 wk)	-8.167	No	ns	0.2268	A-D
Sham (8 wk) vs. ACLr (4 wk)	-11.63	Yes	*	0.0185	B-C
Sham (8 wk) vs. ACLr (8 wk)	-13.97	Yes	**	0.0023	B-D
ACLr (4 wk) vs. ACLr (8 wk)	-2.333	No	ns	>0.9999	C-D
Test details	Mean rank 1	Mean rank 2	Mean rank diff.	n1	n2

Sham (4 wk) vs. Sham (8 wk)	9.000	3.200	5.800	5	5
Sham (4 wk) vs. ACLr (4 wk)	9.000	14.83	-5.833	5	6
Sham (4 wk) vs. ACLr (8 wk)	9.000	17.17	-8.167	5	6
Sham (8 wk) vs. ACLr (4 wk)	3.200	14.83	-11.63	5	6
Sham (8 wk) vs. ACLr (8 wk)	3.200	17.17	-13.97	5	6
ACLr (4 wk) vs. ACLr (8 wk)	14.83	17.17	-2.333	6	6

Medial Cartilage Volume

Tukey's multiple comparisons test	Mean Diff.	95.00% CI of diff.	Below threshold?	Summary	Adjusted P Value	
Sham (4 wk) vs. Sham (8 wk)	0.02670	-0.2451 to 0.2985	No	ns	0.9923	A-B
Sham (4 wk) vs. ACLr (4 wk)	-0.1693	-0.4296 to 0.09096	No	ns	0.2885	A-C
Sham (4 wk) vs. ACLr (8 wk)	-0.3535	-0.6138 to -0.09328	Yes	**	0.0060	A-D
Sham (8 wk) vs. ACLr (4 wk)	-0.1960	-0.4563 to 0.06426	No	ns	0.1818	B-C
Sham (8 wk) vs. ACLr (8 wk)	-0.3802	-0.6405 to -0.1200	Yes	**	0.0032	B-D
ACLr (4 wk) vs. ACLr (8 wk)	-0.1842	-0.4324 to 0.06391	No	ns	0.1912	C-D
Test details	Mean 1	Mean 2	Mean Diff.	SE of diff.	n1	n2
Sham (4 wk) vs. Sham (8 wk)	0.6344	0.6077	0.02670	0.09618	5	5
Sham (4 wk) vs. ACLr (4 wk)	0.6344	0.8037	-0.1693	0.09208	5	6
Sham (4 wk) vs. ACLr (8 wk)	0.6344	0.9880	-0.3535	0.09208	5	6
Sham (8 wk) vs. ACLr (4 wk)	0.6077	0.8037	-0.1960	0.09208	5	6
Sham (8 wk) vs. ACLr (8 wk)	0.6077	0.9880	-0.3802	0.09208	5	6
ACLr (4 wk) vs. ACLr (8 wk)	0.8037	0.9880	-0.1842	0.08780	6	6

Medial Cartilage Attenuation

Tukey's multiple comparisons test	Mean Diff.	95.00% CI of diff.	Below threshold?	Summary	Adjusted P Value	
Sham (4 wk) vs. Sham (8 wk)	-0.05858	-0.3116 to 0.1944	No	ns	0.9126	A-B
Sham (4 wk) vs. ACLr (4 wk)	0.1185	-0.1238 to 0.3607	No	ns	0.5258	A-C
Sham (4 wk) vs. ACLr (8 wk)	0.1844	-0.05782 to 0.4267	No	ns	0.1749	A-D
Sham (8 wk) vs. ACLr (4 wk)	0.1771	-0.06519 to 0.4193	No	ns	0.2019	B-C
Sham (8 wk) vs. ACLr (8 wk)	0.2430	0.0007570 to 0.4852	Yes	*	0.0491	B-D
ACLr (4 wk) vs. ACLr (8 wk)	0.06595	-0.1650 to 0.2969	No	ns	0.8503	C-D
Test details	Mean 1	Mean 2	Mean Diff.	SE of diff.	n1	n2
Sham (4 wk) vs. Sham (8 wk)	2.076	2.135	-0.05858	0.08952	5	5
Sham (4 wk) vs. ACLr (4 wk)	2.076	1.958	0.1185	0.08571	5	6
Sham (4 wk) vs. ACLr (8 wk)	2.076	1.892	0.1844	0.08571	5	6
Sham (8 wk) vs. ACLr (4 wk)	2.135	1.958	0.1771	0.08571	5	6
Sham (8 wk) vs. ACLr (8 wk)	2.135	1.892	0.2430	0.08571	5	6
ACLr (4 wk) vs. ACLr (8 wk)	1.958	1.892	0.06595	0.08172	6	6

Medial 1/3 Medial Cartilage Thickness:

Dunn's multiple comparisons test	Mean rank diff.	Significant?	Summary	Adjusted P Value	
Sham (4 wk) vs. Sham (8 wk)	6.000	No	ns	0.8635	A-B
Sham (4 wk) vs. ACLr (4 wk)	-5.433	No	ns	>0.9999	A-C
Sham (4 wk) vs. ACLr (8 wk)	-7.267	No	ns	0.3871	A-D
Sham (8 wk) vs. ACLr (4 wk)	-11.43	Yes	*	0.0218	B-C
Sham (8 wk) vs. ACLr (8 wk)	-13.27	Yes	**	0.0044	B-D
ACLr (4 wk) vs. ACLr (8 wk)	-1.833	No	ns	>0.9999	C-D
Test details	Mean rank 1	Mean rank 2	Mean rank diff.	n1	n2
Sham (4 wk) vs. Sham (8 wk)	9.400	3.400	6.000	5	5
Sham (4 wk) vs. ACLr (4 wk)	9.400	14.83	-5.433	5	6
Sham (4 wk) vs. ACLr (8 wk)	9.400	16.67	-7.267	5	6
Sham (8 wk) vs. ACLr (4 wk)	3.400	14.83	-11.43	5	6
Sham (8 wk) vs. ACLr (8 wk)	3.400	16.67	-13.27	5	6
ACLr (4 wk) vs. ACLr (8 wk)	14.83	16.67	-1.833	6	6

Medial 1/3 Medial Cartilage Volume

Dunn's multiple comparisons test	Mean rank diff.	Significant?	Summary	Adjusted P Value	
Sham (4 wk) vs. Sham (8 wk)	4.600	No	ns	>0.9999	A-B
Sham (4 wk) vs. ACLr (4 wk)	-6.067	No	ns	0.7372	A-C
Sham (4 wk) vs. ACLr (8 wk)	-8.400	No	ns	0.1959	A-D
Sham (8 wk) vs. ACLr (4 wk)	-10.67	Yes	*	0.0400	B-C
Sham (8 wk) vs. ACLr (8 wk)	-13.00	Yes	**	0.0057	B-D
ACLr (4 wk) vs. ACLr (8 wk)	-2.333	No	ns	>0.9999	C-D
Test details	Mean rank 1	Mean rank 2	Mean rank diff.	n1	n2
Sham (4 wk) vs. Sham (8 wk)	8.600	4.000	4.600	5	5
Sham (4 wk) vs. ACLr (4 wk)	8.600	14.67	-6.067	5	6
Sham (4 wk) vs. ACLr (8 wk)	8.600	17.00	-8.400	5	6
Sham (8 wk) vs. ACLr (4 wk)	4.000	14.67	-10.67	5	6
Sham (8 wk) vs. ACLr (8 wk)	4.000	17.00	-13.00	5	6
ACLr (4 wk) vs. ACLr (8 wk)	14.67	17.00	-2.333	6	6

Medial 1/3 Medial Cartilage Attenuation

Tukey's multiple comparisons test	Mean Diff.	95.00% CI of diff.	Below threshold?	Summary	Adjusted P Value	
Sham (4 wk) vs. Sham (8 wk)	0.001620	-0.2732 to 0.2764	No	ns	>0.9999	A-B
Sham (4 wk) vs. ACLr (4 wk)	0.1225	-0.1406 to 0.3856	No	ns	0.5651	A-C
Sham (4 wk) vs. ACLr (8 wk)	0.2365	-0.02656 to 0.4996	No	ns	0.0869	A-D
Sham (8 wk) vs. ACLr (4 wk)	0.1209	-0.1422 to 0.3839	No	ns	0.5755	B-C

Sham (8 wk) vs. ACLr (8 wk)	0.2349	-0.02818 to 0.4980	No	ns	0.0898	B-D
ACLr (4 wk) vs. ACLr (8 wk)	0.1140	-0.1368 to 0.3649	No	ns	0.5837	C-D
Test details	Mean 1	Mean 2	Mean Diff.	SE of diff.	n1	n2
Sham (4 wk) vs. Sham (8 wk)	1.991	1.990	0.001620	0.09722	5	5
Sham (4 wk) vs. ACLr (4 wk)	1.991	1.869	0.1225	0.09308	5	6
Sham (4 wk) vs. ACLr (8 wk)	1.991	1.755	0.2365	0.09308	5	6
Sham (8 wk) vs. ACLr (4 wk)	1.990	1.869	0.1209	0.09308	5	6
Sham (8 wk) vs. ACLr (8 wk)	1.990	1.755	0.2349	0.09308	5	6
ACLr (4 wk) vs. ACLr (8 wk)	1.869	1.755	0.1140	0.08875	6	6

Lateral 1/3 Medial Cartilage Thickness:

Dunnett's T3 multiple comparisons test	Mean Diff.	95.00% CI of diff.	Below threshold?	Summary	Adjusted P Value	
Sham (4 wk) vs. Sham (8 wk)	0.01656	-0.001608 to 0.03473	No	ns	0.0728	A-B
Sham (4 wk) vs. ACLr (4 wk)	-0.007393	-0.03821 to 0.02342	No	ns	0.9500	A-C
Sham (4 wk) vs. ACLr (8 wk)	-0.04274	-0.09710 to 0.01161	No	ns	0.1261	A-D
Sham (8 wk) vs. ACLr (4 wk)	-0.02395	-0.05445 to 0.006545	No	ns	0.1266	B-C
Sham (8 wk) vs. ACLr (8 wk)	-0.05930	-0.1156 to -0.003008	Yes	*	0.0409	B-D
ACLr (4 wk) vs. ACLr (8 wk)	-0.03535	-0.09010 to 0.01940	No	ns	0.2705	C-D
Test details	Mean 1	Mean 2	Mean Diff.	SE of diff.	n1	n2
Sham (4 wk) vs. Sham (8 wk)	0.08774	0.07118	0.01656	0.004958	5	5
Sham (4 wk) vs. ACLr (4 wk)	0.08774	0.09513	-0.007393	0.009158	5	6
Sham (4 wk) vs. ACLr (8 wk)	0.08774	0.1305	-0.04274	0.01483	5	6
Sham (8 wk) vs. ACLr (4 wk)	0.07118	0.09513	-0.02395	0.008323	5	6
Sham (8 wk) vs. ACLr (8 wk)	0.07118	0.1305	-0.05930	0.01433	5	6
ACLr (4 wk) vs. ACLr (8 wk)	0.09513	0.1305	-0.03535	0.01627	6	6

Lateral 1/3 Medial Cartilage Volume

Dunnett's T3 multiple comparisons test	Mean Diff.	95.00% CI of diff.	Below threshold?	Summary	Adjusted P Value	
Sham (4 wk) vs. Sham (8 wk)	-0.06482	-0.09315 to -0.03649	Yes	***	0.0003	A-B
Sham (4 wk) vs. ACLr (4 wk)	-0.06423	-0.2066 to 0.07810	No	ns	0.4787	A-C
Sham (4 wk) vs. ACLr (8 wk)	-0.1195	-0.2318 to -0.007052	Yes	*	0.0396	A-D
Sham (8 wk) vs. ACLr (4 wk)	0.0005867	-0.1427 to 0.1439	No	ns	>0.9999	B-C
Sham (8 wk) vs. ACLr (8 wk)	-0.05463	-0.1606 to 0.05139	No	ns	0.4113	B-D
ACLr (4 wk) vs. ACLr (8 wk)	-0.05522	-0.2044 to 0.09397	No	ns	0.7773	C-D
Test details	Mean 1	Mean 2	Mean Diff.	SE of diff.	n1	n2
Sham (4 wk) vs. Sham (8 wk)	0.1620	0.2268	-0.06482	0.008419	5	5
Sham (4 wk) vs. ACLr (4 wk)	0.1620	0.2262	-0.06423	0.03624	5	6
Sham (4 wk) vs. ACLr (8 wk)	0.1620	0.2815	-0.1195	0.02862	5	6
Sham (8 wk) vs. ACLr (4 wk)	0.2268	0.2262	0.0005867	0.03649	5	6

Sham (8 wk) vs. ACLr (8 wk)	0.2268	0.2815	-0.05463	0.02893	5	6
ACLr (4 wk) vs. ACLr (8 wk)	0.2262	0.2815	-0.05522	0.04560	6	6

Lateral 1/3 Medial Cartilage Attenuation

Dunn's multiple comparisons test	Mean rank diff.	Significant?	Summary	Adjusted P Value	
Sham (4 wk) vs. Sham (8 wk)	-9.800	No	ns	0.1021	A-B
Sham (4 wk) vs. ACLr (4 wk)	-7.700	No	ns	0.3012	A-C
Sham (4 wk) vs. ACLr (8 wk)	-1.367	No	ns	>0.9999	A-D
Sham (8 wk) vs. ACLr (4 wk)	2.100	No	ns	>0.9999	B-C
Sham (8 wk) vs. ACLr (8 wk)	8.433	No	ns	0.1918	B-D
ACLr (4 wk) vs. ACLr (8 wk)	6.333	No	ns	0.5470	C-D
Test details	Mean rank 1	Mean rank 2	Mean rank diff.	n1	n2
Sham (4 wk) vs. Sham (8 wk)	6.800	16.60	-9.800	5	5
Sham (4 wk) vs. ACLr (4 wk)	6.800	14.50	-7.700	5	6
Sham (4 wk) vs. ACLr (8 wk)	6.800	8.167	-1.367	5	6
Sham (8 wk) vs. ACLr (4 wk)	16.60	14.50	2.100	5	6
Sham (8 wk) vs. ACLr (8 wk)	16.60	8.167	8.433	5	6
ACLr (4 wk) vs. ACLr (8 wk)	14.50	8.167	6.333	6	6

Lateral Cartilage Thickness

Dunnett's T3 multiple comparisons test	Mean Diff.	95.00% CI of diff.	Below threshold?	Summary	Adjusted P Value	
Sham (4 wk) vs. Sham (8 wk)	0.01398	-0.0006144 to 0.02857	No	ns	0.0605	A-B
Sham (4 wk) vs. ACLr (4 wk)	-0.02282	-0.05945 to 0.01381	No	ns	0.2027	A-C
Sham (4 wk) vs. ACLr (8 wk)	-0.03252	-0.07602 to 0.01098	No	ns	0.1497	A-D
Sham (8 wk) vs. ACLr (4 wk)	-0.03680	-0.07217 to -0.001428	Yes	*	0.0438	B-C
Sham (8 wk) vs. ACLr (8 wk)	-0.04650	-0.09233 to -0.0006726	Yes	*	0.0473	B-D
ACLr (4 wk) vs. ACLr (8 wk)	-0.009700	-0.05596 to 0.03656	No	ns	0.9725	C-D
Test details	Mean 1	Mean 2	Mean Diff.	SE of diff.	n1	n2
Sham (4 wk) vs. Sham (8 wk)	0.08948	0.07550	0.01398	0.004183	5	5
Sham (4 wk) vs. ACLr (4 wk)	0.08948	0.1123	-0.02282	0.008389	5	4
Sham (4 wk) vs. ACLr (8 wk)	0.08948	0.1220	-0.03252	0.01187	5	6
Sham (8 wk) vs. ACLr (4 wk)	0.07550	0.1123	-0.03680	0.008101	5	4
Sham (8 wk) vs. ACLr (8 wk)	0.07550	0.1220	-0.04650	0.01167	5	6
ACLr (4 wk) vs. ACLr (8 wk)	0.1123	0.1220	-0.009700	0.01375	4	6

Lateral Cartilage Volume

Tukey's multiple comparisons test	Mean Diff.	95.00% CI of diff.	Below threshold?	Summary	Adjusted P Value

Sham (4 wk) vs. Sham (8 wk)	0.08714	-0.1429 to 0.3172	No	ns	0.7079	A-B
Sham (4 wk) vs. ACLr (4 wk)	-0.2322	-0.4623 to -0.002115	Yes	*	0.0475	A-C
Sham (4 wk) vs. ACLr (8 wk)	-0.2610	-0.4813 to -0.04071	Yes	*	0.0174	A-D
Sham (8 wk) vs. ACLr (4 wk)	-0.3193	-0.5494 to -0.08925	Yes	**	0.0052	B-C
Sham (8 wk) vs. ACLr (8 wk)	-0.3481	-0.5684 to -0.1278	Yes	**	0.0017	B-D
ACLr (4 wk) vs. ACLr (8 wk)	-0.02880	-0.2491 to 0.1915	No	ns	0.9819	C-D
Test details	Mean 1	Mean 2	Mean Diff.	SE of diff.	n1	n2
Sham (4 wk) vs. Sham (8 wk)	0.5874	0.5003	0.08714	0.08094	5	5
Sham (4 wk) vs. ACLr (4 wk)	0.5874	0.8196	-0.2322	0.08094	5	5
Sham (4 wk) vs. ACLr (8 wk)	0.5874	0.8484	-0.2610	0.07750	5	6
Sham (8 wk) vs. ACLr (4 wk)	0.5003	0.8196	-0.3193	0.08094	5	5
Sham (8 wk) vs. ACLr (8 wk)	0.5003	0.8484	-0.3481	0.07750	5	6
ACLr (4 wk) vs. ACLr (8 wk)	0.8196	0.8484	-0.02880	0.07750	5	6

Lateral Cartilage Attenuation

Dunn's multiple comparisons test	Mean rank diff.	Significant?	Summary	Adjusted P Value	
Sham (4 wk) vs. Sham (8 wk)	1.000	No	ns	>0.9999	A-B
Sham (4 wk) vs. ACLr (4 wk)	8.800	No	ns	0.1496	A-C
Sham (4 wk) vs. ACLr (8 wk)	10.03	Yes	*	0.0455	A-D
Sham (8 wk) vs. ACLr (4 wk)	7.800	No	ns	0.2811	B-C
Sham (8 wk) vs. ACLr (8 wk)	9.033	No	ns	0.0972	B-D
ACLr (4 wk) vs. ACLr (8 wk)	1.233	No	ns	>0.9999	C-D
Test details	Mean rank 1	Mean rank 2	Mean rank diff.	n1	n2
Sham (4 wk) vs. Sham (8 wk)	16.20	15.20	1.000	5	5
Sham (4 wk) vs. ACLr (4 wk)	16.20	7.400	8.800	5	5
Sham (4 wk) vs. ACLr (8 wk)	16.20	6.167	10.03	5	6
Sham (8 wk) vs. ACLr (4 wk)	15.20	7.400	7.800	5	5
Sham (8 wk) vs. ACLr (8 wk)	15.20	6.167	9.033	5	6
ACLr (4 wk) vs. ACLr (8 wk)	7.400	6.167	1.233	5	6

Femur Exposed Bone Area

Dunnett's T3 multiple comparisons test	Mean Diff.	95.00% CI of diff.	Below threshold?	Summary	Adjusted P Value	
Sham (4 wk) vs. Sham (8 wk)	-0.05568	-0.1770 to 0.06559	No	ns	0.3995	A-B
Sham (4 wk) vs. ACLr (4 wk)	-0.5151	-1.174 to 0.1435	No	ns	0.1192	A-C
Sham (4 wk) vs. ACLr (8 wk)	-0.7211	-1.174 to -0.2681	Yes	**	0.0072	A-D
Sham (8 wk) vs. ACLr (4 wk)	-0.4594	-1.127 to 0.2079	No	ns	0.1770	B-C
Sham (8 wk) vs. ACLr (8 wk)	-0.6654	-1.100 to -0.2310	Yes	**	0.0068	B-D
ACLr (4 wk) vs. ACLr (8 wk)	-0.2060	-0.8716 to 0.4597	No	ns	0.8787	C-D
Test details	Mean 1	Mean 2	Mean Diff.	SE of diff.	n1	n2

Sham (4 wk) vs. Sham (8 wk)	0.004332	0.06001	-0.05568	0.02778	5	5
Sham (4 wk) vs. ACLr (4 wk)	0.004332	0.5195	-0.5151	0.1677	5	6
Sham (4 wk) vs. ACLr (8 wk)	0.004332	0.7254	-0.7211	0.1153	5	6
Sham (8 wk) vs. ACLr (4 wk)	0.06001	0.5195	-0.4594	0.1699	5	6
Sham (8 wk) vs. ACLr (8 wk)	0.06001	0.7254	-0.6654	0.1185	5	6
ACLr (4 wk) vs. ACLr (8 wk)	0.5195	0.7254	-0.2060	0.2035	6	6

Femur Lesion Area

Dunnett's T3 multiple comparisons test	Mean Diff.	95.00% CI of diff.	Below threshold?	Summary	Adjusted P Value	
Sham (4 wk) vs. Sham (8 wk)	-0.01212	-0.03724 to 0.01300	No	ns	0.3630	A-B
Sham (4 wk) vs. ACLr (4 wk)	-0.1622	-0.4176 to 0.09318	No	ns	0.2224	A-C
Sham (4 wk) vs. ACLr (8 wk)	-0.3078	-0.5368 to -0.07871	Yes	*	0.0152	A-D
Sham (8 wk) vs. ACLr (4 wk)	-0.1501	-0.4064 to 0.1062	No	ns	0.2749	B-C
Sham (8 wk) vs. ACLr (8 wk)	-0.2956	-0.5257 to -0.06558	Yes	*	0.0183	B-D
ACLr (4 wk) vs. ACLr (8 wk)	-0.1455	-0.4249 to 0.1339	No	ns	0.4990	C-D
Test details	Mean 1	Mean 2	Mean Diff.	SE of diff.	n1	n2
Sham (4 wk) vs. Sham (8 wk)	0.002288	0.01441	-0.01212	0.005754	5	5
Sham (4 wk) vs. ACLr (4 wk)	0.002288	0.1645	-0.1622	0.06503	5	6
Sham (4 wk) vs. ACLr (8 wk)	0.002288	0.3100	-0.3078	0.05832	5	6
Sham (8 wk) vs. ACLr (4 wk)	0.01441	0.1645	-0.1501	0.06526	5	6
Sham (8 wk) vs. ACLr (8 wk)	0.01441	0.3100	-0.2956	0.05858	5	6
ACLr (4 wk) vs. ACLr (8 wk)	0.1645	0.3100	-0.1455	0.08733	6	6

Femur Lesion Volume

Tukey's multiple comparisons test	Mean Diff.	95.00% CI of diff.	Below threshold?	Summary	Adjusted P Value	
Sham (4 wk) vs. Sham (8 wk)	-0.0007803	-0.004693 to 0.003132	No	ns	0.9405	A-B
Sham (4 wk) vs. ACLr (4 wk)	-0.003230	-0.006976 to 0.0005158	No	ns	0.1048	A-C
Sham (4 wk) vs. ACLr (8 wk)	-0.005777	-0.009689 to -0.001865	Yes	**	0.0031	A-D
Sham (8 wk) vs. ACLr (4 wk)	-0.002450	-0.006195 to 0.001296	No	ns	0.2816	B-C
Sham (8 wk) vs. ACLr (8 wk)	-0.004997	-0.008909 to -0.001084	Yes	*	0.0101	B-D
ACLr (4 wk) vs. ACLr (8 wk)	-0.002547	-0.006293 to 0.001199	No	ns	0.2517	C-D
Test details	Mean 1	Mean 2	Mean Diff.	SE of diff.	n1	n2
Sham (4 wk) vs. Sham (8 wk)	8.875e-005	0.0008690	-0.0007803	0.001376	5	5
Sham (4 wk) vs. ACLr (4 wk)	8.875e-005	0.003319	-0.003230	0.001318	5	6
Sham (4 wk) vs. ACLr (8 wk)	8.875e-005	0.005866	-0.005777	0.001376	5	5
Sham (8 wk) vs. ACLr (4 wk)	0.0008690	0.003319	-0.002450	0.001318	5	6
Sham (8 wk) vs. ACLr (8 wk)	0.0008690	0.005866	-0.004997	0.001376	5	5
ACLr (4 wk) vs. ACLr (8 wk)	0.003319	0.005866	-0.002547	0.001318	6	5

TIBIAL ARTICULAR CARTILAGE MORPHOMETRICS

Medial Cartilage Thickness

Tukey's multiple comparisons test	Mean Diff.	95.00% CI of diff.	Below threshold?	Summary	Adjusted P Value	
Sham (4 wk) vs. Sham (8 wk)	-0.01196	-0.07474 to 0.05081	No	ns	0.9484	A-B
Sham (4 wk) vs. ACLr (4 wk)	-0.04100	-0.1066 to 0.02457	No	ns	0.3203	A-C
Sham (4 wk) vs. ACLr (8 wk)	-0.07236	-0.1351 to -0.009588	Yes	*	0.0207	A-D
Sham (8 wk) vs. ACLr (4 wk)	-0.02904	-0.09181 to 0.03374	No	ns	0.5703	B-C
Sham (8 wk) vs. ACLr (8 wk)	-0.06040	-0.1203 to -0.0005460	Yes	*	0.0475	B-D
ACLr (4 wk) vs. ACLr (8 wk)	-0.03136	-0.09414 to 0.03141	No	ns	0.5083	C-D
Test details	Mean 1	Mean 2	Mean Diff.	SE of diff.	n1	n2
Sham (4 wk) vs. Sham (8 wk)	0.1939	0.2059	-0.01196	0.02221	5	6
Sham (4 wk) vs. ACLr (4 wk)	0.1939	0.2349	-0.04100	0.02320	5	5
Sham (4 wk) vs. ACLr (8 wk)	0.1939	0.2663	-0.07236	0.02221	5	6
Sham (8 wk) vs. ACLr (4 wk)	0.2059	0.2349	-0.02904	0.02221	6	5
Sham (8 wk) vs. ACLr (8 wk)	0.2059	0.2663	-0.06040	0.02118	6	6
ACLr (4 wk) vs. ACLr (8 wk)	0.2349	0.2663	-0.03136	0.02221	5	6

Medial Cartilage Volume

Tukey's multiple comparisons test	Mean Diff.	95.00% CI of diff.	Below threshold?	Summary	Adjusted P Value	
Sham (4 wk) vs. Sham (8 wk)	0.1515	-0.1684 to 0.4714	No	ns	0.5476	A-B
Sham (4 wk) vs. ACLr (4 wk)	0.04769	-0.2586 to 0.3540	No	ns	0.9702	A-C
Sham (4 wk) vs. ACLr (8 wk)	0.009520	-0.3104 to 0.3294	No	ns	0.9998	A-D
Sham (8 wk) vs. ACLr (4 wk)	-0.1039	-0.4101 to 0.2024	No	ns	0.7713	B-C
Sham (8 wk) vs. ACLr (8 wk)	-0.1420	-0.4619 to 0.1779	No	ns	0.5980	B-D
ACLr (4 wk) vs. ACLr (8 wk)	-0.03817	-0.3444 to 0.2681	No	ns	0.9842	C-D
Test details	Mean 1	Mean 2	Mean Diff.	SE of diff.	n1	n2
Sham (4 wk) vs. Sham (8 wk)	1.211	1.059	0.1515	0.1125	5	5
Sham (4 wk) vs. ACLr (4 wk)	1.211	1.163	0.04769	0.1077	5	6
Sham (4 wk) vs. ACLr (8 wk)	1.211	1.201	0.009520	0.1125	5	5
Sham (8 wk) vs. ACLr (4 wk)	1.059	1.163	-0.1039	0.1077	5	6
Sham (8 wk) vs. ACLr (8 wk)	1.059	1.201	-0.1420	0.1125	5	5
ACLr (4 wk) vs. ACLr (8 wk)	1.163	1.201	-0.03817	0.1077	6	5

Medial Cartilage Attenuation

Tukey's multiple comparisons test	Mean Diff.	95.00% CI of diff.	Below threshold?	Summary	Adjusted P Value	
Sham (4 wk) vs. Sham (8 wk)	-0.04972	-0.3133 to 0.2138	No	ns	0.9506	A-B

Sham (4 wk) vs. ACLr (4 wk)	-0.2322	-0.4958 to 0.03131	No	ns	0.0960	A-C
Sham (4 wk) vs. ACLr (8 wk)	-0.07712	-0.3407 to 0.1864	No	ns	0.8430	A-D
Sham (8 wk) vs. ACLr (4 wk)	-0.1825	-0.4338 to 0.06877	No	ns	0.2079	B-C
Sham (8 wk) vs. ACLr (8 wk)	-0.02740	-0.2787 to 0.2239	No	ns	0.9897	B-D
ACLr (4 wk) vs. ACLr (8 wk)	0.1551	-0.09617 to 0.4064	No	ns	0.3337	C-D
Test details	Mean 1	Mean 2	Mean Diff.	SE of diff.	n1	n2
Sham (4 wk) vs. Sham (8 wk)	1.876	1.926	-0.04972	0.09373	5	6
Sham (4 wk) vs. ACLr (4 wk)	1.876	2.109	-0.2322	0.09373	5	6
Sham (4 wk) vs. ACLr (8 wk)	1.876	1.954	-0.07712	0.09373	5	6
Sham (8 wk) vs. ACLr (4 wk)	1.926	2.109	-0.1825	0.08937	6	6
Sham (8 wk) vs. ACLr (8 wk)	1.926	1.954	-0.02740	0.08937	6	6
ACLr (4 wk) vs. ACLr (8 wk)	2.109	1.954	0.1551	0.08937	6	6

Medial 1/3 Medial Cartilage Thickness

Tukey's multiple comparisons test	Mean Diff.	95.00% CI of diff.	Below threshold?	Summary	Adjusted P Value	
Sham (4 wk) vs. Sham (8 wk)	0.02526	-0.01355 to 0.06406	No	ns	0.2906	A-B
Sham (4 wk) vs. ACLr (4 wk)	-0.03158	-0.07038 to 0.007230	No	ns	0.1362	A-C
Sham (4 wk) vs. ACLr (8 wk)	-0.03751	-0.07632 to 0.001297	No	ns	0.0603	A-D
Sham (8 wk) vs. ACLr (4 wk)	-0.05683	-0.09383 to -0.01983	Yes	**	0.0019	B-C
Sham (8 wk) vs. ACLr (8 wk)	-0.06277	-0.09977 to -0.02577	Yes	***	0.0007	B-D
ACLr (4 wk) vs. ACLr (8 wk)	-0.005933	-0.04293 to 0.03107	No	ns	0.9686	C-D
Test details	Mean 1	Mean 2	Mean Diff.	SE of diff.	n1	n2
Sham (4 wk) vs. Sham (8 wk)	0.1570	0.1318	0.02526	0.01380	5	6
Sham (4 wk) vs. ACLr (4 wk)	0.1570	0.1886	-0.03158	0.01380	5	6
Sham (4 wk) vs. ACLr (8 wk)	0.1570	0.1946	-0.03751	0.01380	5	6
Sham (8 wk) vs. ACLr (4 wk)	0.1318	0.1886	-0.05683	0.01316	6	6
Sham (8 wk) vs. ACLr (8 wk)	0.1318	0.1946	-0.06277	0.01316	6	6
ACLr (4 wk) vs. ACLr (8 wk)	0.1886	0.1946	-0.005933	0.01316	6	6

Medial 1/3 Medial Cartilage Volume

Tukey's multiple comparisons test	Mean Diff.	95.00% CI of diff.	Below threshold?	Summary	Adjusted P Value	
Sham (4 wk) vs. Sham (8 wk)	0.07110	0.0005964 to 0.1416	Yes	*	0.0477	A-B
Sham (4 wk) vs. ACLr (4 wk)	0.1453	0.07776 to 0.2128	Yes	****	<0.0001	A-C
Sham (4 wk) vs. ACLr (8 wk)	0.009480	-0.06102 to 0.07998	No	ns	0.9804	A-D
Sham (8 wk) vs. ACLr (4 wk)	0.07416	0.006661 to 0.1417	Yes	*	0.0286	B-C
Sham (8 wk) vs. ACLr (8 wk)	-0.06162	-0.1321 to 0.008884	No	ns	0.0986	B-D
ACLr (4 wk) vs. ACLr (8 wk)	-0.1358	-0.2033 to -0.06828	Yes	***	0.0001	C-D
Test details	Mean 1	Mean 2	Mean Diff.	SE of diff.	n1	n2
Sham (4 wk) vs. Sham (8 wk)	0.3339	0.2628	0.07110	0.02480	5	5

Sham (4 wk) vs. ACLr (4 wk)	0.3339	0.1886	0.1453	0.02375	5	6
Sham (4 wk) vs. ACLr (8 wk)	0.3339	0.3244	0.009480	0.02480	5	5
Sham (8 wk) vs. ACLr (4 wk)	0.2628	0.1886	0.07416	0.02375	5	6
Sham (8 wk) vs. ACLr (8 wk)	0.2628	0.3244	-0.06162	0.02480	5	5
ACLr (4 wk) vs. ACLr (8 wk)	0.1886	0.3244	-0.1358	0.02375	6	5

Medial 1/3 Medial Cartilage Attenuation

Tukey's multiple comparisons test	Mean Diff.	95.00% CI of diff.	Below threshold?	Summary	Adjusted P Value	
Sham (4 wk) vs. Sham (8 wk)	-0.05723	-0.3762 to 0.2617	No	ns	0.9570	A-B
Sham (4 wk) vs. ACLr (4 wk)	-0.3751	-0.6941 to -0.05614	Yes	*	0.0178	A-C
Sham (4 wk) vs. ACLr (8 wk)	-0.09761	-0.4166 to 0.2214	No	ns	0.8248	A-D
Sham (8 wk) vs. ACLr (4 wk)	-0.3179	-0.6220 to -0.01375	Yes	*	0.0386	B-C
Sham (8 wk) vs. ACLr (8 wk)	-0.04038	-0.3445 to 0.2637	No	ns	0.9817	B-D
ACLr (4 wk) vs. ACLr (8 wk)	0.2775	-0.02663 to 0.5816	No	ns	0.0812	C-D
Test details	Mean 1	Mean 2	Mean Diff.	SE of diff.	n1	n2
Sham (4 wk) vs. Sham (8 wk)	1.840	1.898	-0.05723	0.1134	5	6
Sham (4 wk) vs. ACLr (4 wk)	1.840	2.216	-0.3751	0.1134	5	6
Sham (4 wk) vs. ACLr (8 wk)	1.840	1.938	-0.09761	0.1134	5	6
Sham (8 wk) vs. ACLr (4 wk)	1.898	2.216	-0.3179	0.1082	6	6
Sham (8 wk) vs. ACLr (8 wk)	1.898	1.938	-0.04038	0.1082	6	6
ACLr (4 wk) vs. ACLr (8 wk)	2.216	1.938	0.2775	0.1082	6	6

Central 1/3 Medial Cartilage Thickness

Tukey's multiple comparisons test	Mean Diff.	95.00% CI of diff.	Below threshold?	Summary	Adjusted P Value	
Sham (4 wk) vs. Sham (8 wk)	0.02572	-0.02577 to 0.07721	No	ns	0.5118	A-B
Sham (4 wk) vs. ACLr (4 wk)	-0.04025	-0.09173 to 0.01124	No	ns	0.1597	A-C
Sham (4 wk) vs. ACLr (8 wk)	-0.08036	-0.1318 to -0.02888	Yes	**	0.0016	A-D
Sham (8 wk) vs. ACLr (4 wk)	-0.06597	-0.1151 to -0.01688	Yes	**	0.0064	B-C
Sham (8 wk) vs. ACLr (8 wk)	-0.1061	-0.1552 to -0.05699	Yes	****	<0.0001	B-D
ACLr (4 wk) vs. ACLr (8 wk)	-0.04012	-0.08921 to 0.008973	No	ns	0.1338	C-D
Test details	Mean 1	Mean 2	Mean Diff.	SE of diff.	n1	n2
Sham (4 wk) vs. Sham (8 wk)	0.2076	0.1819	0.02572	0.01831	5	6
Sham (4 wk) vs. ACLr (4 wk)	0.2076	0.2479	-0.04025	0.01831	5	6
Sham (4 wk) vs. ACLr (8 wk)	0.2076	0.2880	-0.08036	0.01831	5	6
Sham (8 wk) vs. ACLr (4 wk)	0.1819	0.2479	-0.06597	0.01746	6	6
Sham (8 wk) vs. ACLr (8 wk)	0.1819	0.2880	-0.1061	0.01746	6	6
ACLr (4 wk) vs. ACLr (8 wk)	0.2479	0.2880	-0.04012	0.01746	6	6

Central 1/3 Medial Cartilage Volume

Tukey's multiple comparisons test	Mean Diff.	95.00% CI of diff.	Below threshold?	Summary	Adjusted P Value	
Sham (4 wk) vs. Sham (8 wk)	-0.03440	-0.2163 to 0.1475	No	ns	0.9495	A-B
Sham (4 wk) vs. ACLr (4 wk)	-0.1087	-0.2828 to 0.06550	No	ns	0.3221	A-C
Sham (4 wk) vs. ACLr (8 wk)	-0.1729	-0.3471 to 0.001232	No	ns	0.0520	A-D
Sham (8 wk) vs. ACLr (4 wk)	-0.07427	-0.2484 to 0.09990	No	ns	0.6315	B-C
Sham (8 wk) vs. ACLr (8 wk)	-0.1385	-0.3127 to 0.03563	No	ns	0.1481	B-D
ACLr (4 wk) vs. ACLr (8 wk)	-0.06427	-0.2303 to 0.1018	No	ns	0.6977	C-D
Test details	Mean 1	Mean 2	Mean Diff.	SE of diff.	n1	n2
Sham (4 wk) vs. Sham (8 wk)	0.4717	0.5061	-0.03440	0.06436	5	5
Sham (4 wk) vs. ACLr (4 wk)	0.4717	0.5804	-0.1087	0.06162	5	6
Sham (4 wk) vs. ACLr (8 wk)	0.4717	0.6446	-0.1729	0.06162	5	6
Sham (8 wk) vs. ACLr (4 wk)	0.5061	0.5804	-0.07427	0.06162	5	6
Sham (8 wk) vs. ACLr (8 wk)	0.5061	0.6446	-0.1385	0.06162	5	6
ACLr (4 wk) vs. ACLr (8 wk)	0.5804	0.6446	-0.06427	0.05876	6	6

Central 1/3 Medial Cartilage Attenuation

Tukey's multiple comparisons test	Mean Diff.	95.00% CI of diff.	Below threshold?	Summary	Adjusted P Value	
Sham (4 wk) vs. Sham (8 wk)	-0.07917	-0.3514 to 0.1931	No	ns	0.8454	A-B
Sham (4 wk) vs. ACLr (4 wk)	-0.2829	-0.5551 to -0.01057	Yes	*	0.0400	A-C
Sham (4 wk) vs. ACLr (8 wk)	-0.1107	-0.3829 to 0.1616	No	ns	0.6685	A-D
Sham (8 wk) vs. ACLr (4 wk)	-0.2037	-0.4633 to 0.05592	No	ns	0.1574	B-C
Sham (8 wk) vs. ACLr (8 wk)	-0.03150	-0.2911 to 0.2281	No	ns	0.9859	B-D
ACLr (4 wk) vs. ACLr (8 wk)	0.1722	-0.08742 to 0.4318	No	ns	0.2756	C-D
Test details	Mean 1	Mean 2	Mean Diff.	SE of diff.	n1	n2
Sham (4 wk) vs. Sham (8 wk)	1.846	1.925	-0.07917	0.09683	5	6
Sham (4 wk) vs. ACLr (4 wk)	1.846	2.129	-0.2829	0.09683	5	6
Sham (4 wk) vs. ACLr (8 wk)	1.846	1.957	-0.1107	0.09683	5	6
Sham (8 wk) vs. ACLr (4 wk)	1.925	2.129	-0.2037	0.09233	6	6
Sham (8 wk) vs. ACLr (8 wk)	1.925	1.957	-0.03150	0.09233	6	6
ACLr (4 wk) vs. ACLr (8 wk)	2.129	1.957	0.1722	0.09233	6	6

Lateral Cartilage Thickness

Tukey's multiple comparisons test	Mean Diff.	95.00% CI of diff.	Below threshold?	Summary	Adjusted P Value	
Sham (4 wk) vs. Sham (8 wk)	0.03411	-0.01441 to 0.08263	No	ns	0.2273	A-B
Sham (4 wk) vs. ACLr (4 wk)	-0.02580	-0.07249 to 0.02089	No	ns	0.4203	A-C
Sham (4 wk) vs. ACLr (8 wk)	-0.05245	-0.09914 to -0.005757	Yes	*	0.0248	A-D
Sham (8 wk) vs. ACLr (4 wk)	-0.05991	-0.1037 to -0.01611	Yes	**	0.0059	B-C

Sham (8 wk) vs. ACLr (8 wk)	-0.08656	-0.1304 to -0.04276	Yes	***	0.0002	B-D
ACLr (4 wk) vs. ACLr (8 wk)	-0.02665	-0.06841 to 0.01511	No	ns	0.3010	C-D
Test details	Mean 1	Mean 2	Mean Diff.	SE of diff.	n1	n2
Sham (4 wk) vs. Sham (8 wk)	0.1835	0.1493	0.03411	0.01707	4	5
Sham (4 wk) vs. ACLr (4 wk)	0.1835	0.2093	-0.02580	0.01643	4	6
Sham (4 wk) vs. ACLr (8 wk)	0.1835	0.2359	-0.05245	0.01643	4	6
Sham (8 wk) vs. ACLr (4 wk)	0.1493	0.2093	-0.05991	0.01541	5	6
Sham (8 wk) vs. ACLr (8 wk)	0.1493	0.2359	-0.08656	0.01541	5	6
ACLr (4 wk) vs. ACLr (8 wk)	0.2093	0.2359	-0.02665	0.01469	6	6

Lateral Cartilage Volume

Tukey's multiple comparisons test	Mean Diff.	95.00% CI of diff.	Below threshold?	Summary	Adjusted P Value	
Sham (4 wk) vs. Sham (8 wk)	-0.1321	-0.5156 to 0.2514	No	ns	0.7658	A-B
Sham (4 wk) vs. ACLr (4 wk)	-0.2806	-0.6641 to 0.1029	No	ns	0.2011	A-C
Sham (4 wk) vs. ACLr (8 wk)	-0.4276	-0.8111 to -0.04412	Yes	*	0.0258	A-D
Sham (8 wk) vs. ACLr (4 wk)	-0.1485	-0.4915 to 0.1945	No	ns	0.6206	B-C
Sham (8 wk) vs. ACLr (8 wk)	-0.2955	-0.6385 to 0.04752	No	ns	0.1059	B-D
ACLr (4 wk) vs. ACLr (8 wk)	-0.1470	-0.4900 to 0.1960	No	ns	0.6279	C-D
Test details	Mean 1	Mean 2	Mean Diff.	SE of diff.	n1	n2
Sham (4 wk) vs. Sham (8 wk)	0.9975	1.130	-0.1321	0.1357	4	6
Sham (4 wk) vs. ACLr (4 wk)	0.9975	1.278	-0.2806	0.1357	4	6
Sham (4 wk) vs. ACLr (8 wk)	0.9975	1.425	-0.4276	0.1357	4	6
Sham (8 wk) vs. ACLr (4 wk)	1.130	1.278	-0.1485	0.1214	6	6
Sham (8 wk) vs. ACLr (8 wk)	1.130	1.425	-0.2955	0.1214	6	6
ACLr (4 wk) vs. ACLr (8 wk)	1.278	1.425	-0.1470	0.1214	6	6

Lateral Cartilage Attenuation

Dunn's multiple comparisons test	Mean rank diff.	Significant?	Summary	Adjusted P Value	
Sham (4 wk) vs. Sham (8 wk)	1.667	No	ns	>0.9999	A-B
Sham (4 wk) vs. ACLr (4 wk)	-5.167	No	ns	>0.9999	A-C
Sham (4 wk) vs. ACLr (8 wk)	4.417	No	ns	>0.9999	A-D
Sham (8 wk) vs. ACLr (4 wk)	-6.833	No	ns	0.4097	B-C
Sham (8 wk) vs. ACLr (8 wk)	2.750	No	ns	>0.9999	B-D
ACLr (4 wk) vs. ACLr (8 wk)	9.583	No	ns	0.0634	C-D
Test details	Mean rank 1	Mean rank 2	Mean rank diff.	n1	n2
Sham (4 wk) vs. Sham (8 wk)	11.75	10.08	1.667	4	6
Sham (4 wk) vs. ACLr (4 wk)	11.75	16.92	-5.167	4	6
Sham (4 wk) vs. ACLr (8 wk)	11.75	7.333	4.417	4	6
Sham (8 wk) vs. ACLr (4 wk)	10.08	16.92	-6.833	6	6

Sham (8 wk) vs. ACLr (8 wk)	10.08	7.333	2.750	6	6
ACLr (4 wk) vs. ACLr (8 wk)	16.92	7.333	9.583	6	6

Tibia Lesion Area

Dunnett's T3 multiple comparisons test	Mean Diff.	95.00% CI of diff.	Below threshold?	Summary	Adjusted P Value	
Sham (4 wk) vs. Sham (8 wk)	-0.004770	-0.01864 to 0.009102	No	ns	0.6997	A-B
Sham (4 wk) vs. ACLr (4 wk)	-0.006514	-0.02302 to 0.009991	No	ns	0.5912	A-C
Sham (4 wk) vs. ACLr (8 wk)	-0.0002677	-0.001652 to 0.001117	No	ns	0.9697	A-D
Sham (8 wk) vs. ACLr (4 wk)	-0.001744	-0.01930 to 0.01581	No	ns	0.9996	B-C
Sham (8 wk) vs. ACLr (8 wk)	0.004502	-0.009438 to 0.01844	No	ns	0.7443	B-D
ACLr (4 wk) vs. ACLr (8 wk)	0.006246	-0.01032 to 0.02281	No	ns	0.6283	C-D
Test details	Mean 1	Mean 2	Mean Diff.	SE of diff.	n1	n2
Sham (4 wk) vs. Sham (8 wk)	9.734e-005	0.004867	-0.004770	0.003532	5	6
Sham (4 wk) vs. ACLr (4 wk)	9.734e-005	0.006611	-0.006514	0.004202	5	6
Sham (4 wk) vs. ACLr (8 wk)	9.734e-005	0.0003650	-0.0002677	0.0003778	5	6
Sham (8 wk) vs. ACLr (4 wk)	0.004867	0.006611	-0.001744	0.005488	6	6
Sham (8 wk) vs. ACLr (8 wk)	0.004867	0.0003650	0.004502	0.003549	6	6
ACLr (4 wk) vs. ACLr (8 wk)	0.006611	0.0003650	0.006246	0.004217	6	6

Tibia Surface Roughness

Tukey's multiple comparisons test	Mean Diff.	95.00% CI of diff.	Below threshold?	Summary	Adjusted P Value	
Sham (4 wk) vs. Sham (8 wk)	-7.416	-16.73 to 1.897	No	ns	0.1475	A-B
Sham (4 wk) vs. ACLr (4 wk)	-12.76	-22.07 to -3.445	Yes	**	0.0056	A-C
Sham (4 wk) vs. ACLr (8 wk)	-5.077	-14.80 to 4.650	No	ns	0.4719	A-D
Sham (8 wk) vs. ACLr (4 wk)	-5.343	-14.22 to 3.537	No	ns	0.3521	B-C
Sham (8 wk) vs. ACLr (8 wk)	2.339	-6.974 to 11.65	No	ns	0.8919	B-D
ACLr (4 wk) vs. ACLr (8 wk)	7.681	-1.632 to 16.99	No	ns	0.1279	C-D
Test details	Mean 1	Mean 2	Mean Diff.	SE of diff.	n1	n2
Sham (4 wk) vs. Sham (8 wk)	13.37	20.78	-7.416	3.295	5	6
Sham (4 wk) vs. ACLr (4 wk)	13.37	26.13	-12.76	3.295	5	6
Sham (4 wk) vs. ACLr (8 wk)	13.37	18.44	-5.077	3.442	5	5
Sham (8 wk) vs. ACLr (4 wk)	20.78	26.13	-5.343	3.142	6	6
Sham (8 wk) vs. ACLr (8 wk)	20.78	18.44	2.339	3.295	6	5
ACLr (4 wk) vs. ACLr (8 wk)	26.13	18.44	7.681	3.295	6	5

Overall Osteophyte Volume

Tukey's multiple comparisons test	Mean Diff.	95.00% CI of diff.	Below threshold?	Summary	Adjusted P Value	
Sham (4 wk) vs. Sham (8 wk)	-0.1212	-0.4341 to 0.1917	No	ns	0.6935	A-B
Sham (4 wk) vs. ACLr (4 wk)	-0.3906	-0.6902 to -0.09105	Yes	**	0.0086	A-C
Sham (4 wk) vs. ACLr (8 wk)	-0.4381	-0.7510 to -0.1252	Yes	**	0.0048	A-D
Sham (8 wk) vs. ACLr (4 wk)	-0.2694	-0.5690 to 0.03019	No	ns	0.0864	B-C
Sham (8 wk) vs. ACLr (8 wk)	-0.3169	-0.6298 to -0.003992	Yes	*	0.0466	B-D
ACLr (4 wk) vs. ACLr (8 wk)	-0.04751	-0.3471 to 0.2521	No	ns	0.9686	C-D
Test details	Mean 1	Mean 2	Mean Diff.	SE of diff.	n1	n2
Sham (4 wk) vs. Sham (8 wk)	0.2106	0.3318	-0.1212	0.1101	5	5
Sham (4 wk) vs. ACLr (4 wk)	0.2106	0.6012	-0.3906	0.1054	5	6
Sham (4 wk) vs. ACLr (8 wk)	0.2106	0.6487	-0.4381	0.1101	5	5
Sham (8 wk) vs. ACLr (4 wk)	0.3318	0.6012	-0.2694	0.1054	5	6
Sham (8 wk) vs. ACLr (8 wk)	0.3318	0.6487	-0.3169	0.1101	5	5
ACLr (4 wk) vs. ACLr (8 wk)	0.6012	0.6487	-0.04751	0.1054	6	5

Cartilaginous Osteophyte Volume

Tukey's multiple comparisons test	Mean Diff.	95.00% CI of diff.	Below threshold?	Summary	Adjusted P Value	
Sham (4 wk) vs. Sham (8 wk)	6.000e-005	-0.05107 to 0.05119	No	ns	>0.9999	A-B
Sham (4 wk) vs. ACLr (4 wk)	-0.03060	-0.08173 to 0.02053	No	ns	0.3533	A-C
Sham (4 wk) vs. ACLr (8 wk)	-0.05676	-0.1057 to -0.007814	Yes	*	0.0201	A-D
Sham (8 wk) vs. ACLr (4 wk)	-0.03066	-0.08179 to 0.02047	No	ns	0.3517	B-C
Sham (8 wk) vs. ACLr (8 wk)	-0.05682	-0.1058 to -0.007874	Yes	*	0.0200	B-D
ACLr (4 wk) vs. ACLr (8 wk)	-0.02616	-0.07511 to 0.02279	No	ns	0.4483	C-D
Test details	Mean 1	Mean 2	Mean Diff.	SE of diff.	n1	n2
Sham (4 wk) vs. Sham (8 wk)	0.01522	0.01516	6.000e-005	0.01799	5	5
Sham (4 wk) vs. ACLr (4 wk)	0.01522	0.04582	-0.03060	0.01799	5	5
Sham (4 wk) vs. ACLr (8 wk)	0.01522	0.07198	-0.05676	0.01722	5	6
Sham (8 wk) vs. ACLr (4 wk)	0.01516	0.04582	-0.03066	0.01799	5	5
Sham (8 wk) vs. ACLr (8 wk)	0.01516	0.07198	-0.05682	0.01722	5	6
ACLr (4 wk) vs. ACLr (8 wk)	0.04582	0.07198	-0.02616	0.01722	5	6

Mineralized Osteophyte Volume

Tukey's multiple comparisons test	Mean Diff.	95.00% CI of diff.	Below threshold?	Summary	Adjusted P Value	
Sham (4 wk) vs. Sham (8 wk)	-0.06285	-0.3183 to 0.1926	No	ns	0.8975	A-B

Sham (4 wk) vs. ACLr (4 wk)	-0.3201	-0.5756 to -0.06467	Yes	*	0.0114	A-C
Sham (4 wk) vs. ACLr (8 wk)	-0.3699	-0.6367 to -0.1030	Yes	**	0.0051	A-D
Sham (8 wk) vs. ACLr (4 wk)	-0.2573	-0.5009 to -0.01371	Yes	*	0.0363	B-C
Sham (8 wk) vs. ACLr (8 wk)	-0.3070	-0.5625 to -0.05154	Yes	*	0.0155	B-D
ACLr (4 wk) vs. ACLr (8 wk)	-0.04972	-0.3052 to 0.2057	No	ns	0.9453	C-D
Test details	Mean 1	Mean 2	Mean Diff.	SE of diff.	n1	n2
Sham (4 wk) vs. Sham (8 wk)	0.1954	0.2582	-0.06285	0.09039	5	6
Sham (4 wk) vs. ACLr (4 wk)	0.1954	0.5155	-0.3201	0.09039	5	6
Sham (4 wk) vs. ACLr (8 wk)	0.1954	0.5652	-0.3699	0.09441	5	5
Sham (8 wk) vs. ACLr (4 wk)	0.2582	0.5155	-0.2573	0.08618	6	6
Sham (8 wk) vs. ACLr (8 wk)	0.2582	0.5652	-0.3070	0.09039	6	5
ACLr (4 wk) vs. ACLr (8 wk)	0.5155	0.5652	-0.04972	0.09039	6	5

Tibia SBP.Th

Tukey's multiple comparisons test	Mean Diff.	95.00% CI of diff.	Below threshold?	Summary	Adjusted P Value	
Sham (4 wk) vs. Sham (8 wk)	-0.01199	-0.04512 to 0.02115	No	ns	0.7416	A-B
Sham (4 wk) vs. ACLr (4 wk)	0.01447	-0.01866 to 0.04761	No	ns	0.6172	A-C
Sham (4 wk) vs. ACLr (8 wk)	0.02421	-0.008927 to 0.05734	No	ns	0.2038	A-D
Sham (8 wk) vs. ACLr (4 wk)	0.02646	-0.005133 to 0.05805	No	ns	0.1206	B-C
Sham (8 wk) vs. ACLr (8 wk)	0.03619	0.004600 to 0.06778	Yes	*	0.0214	B-D
ACLr (4 wk) vs. ACLr (8 wk)	0.009733	-0.02186 to 0.04132	No	ns	0.8219	C-D
Test details	Mean 1	Mean 2	Mean Diff.	SE of diff.	n1	n2
Sham (4 wk) vs. Sham (8 wk)	0.1832	0.1952	-0.01199	0.01178	5	6
Sham (4 wk) vs. ACLr (4 wk)	0.1832	0.1688	0.01447	0.01178	5	6
Sham (4 wk) vs. ACLr (8 wk)	0.1832	0.1590	0.02421	0.01178	5	6
Sham (8 wk) vs. ACLr (4 wk)	0.1952	0.1688	0.02646	0.01124	6	6
Sham (8 wk) vs. ACLr (8 wk)	0.1952	0.1590	0.03619	0.01124	6	6
ACLr (4 wk) vs. ACLr (8 wk)	0.1688	0.1590	0.009733	0.01124	6	6

Tibia SBP.BV

Tukey's multiple comparisons test	Mean Diff.	95.00% CI of diff.	Below threshold?	Summary	Adjusted P Value	
Sham (4 wk) vs. Sham (8 wk)	-0.3162	-0.6442 to 0.01176	No	ns	0.0612	A-B
Sham (4 wk) vs. ACLr (4 wk)	0.05987	-0.2681 to 0.3879	No	ns	0.9549	A-C
Sham (4 wk) vs. ACLr (8 wk)	0.1037	-0.2243 to 0.4317	No	ns	0.8106	A-D
Sham (8 wk) vs. ACLr (4 wk)	0.3761	0.06338 to 0.6889	Yes	*	0.0152	B-C
Sham (8 wk) vs. ACLr (8 wk)	0.4199	0.1072 to 0.7327	Yes	**	0.0064	B-D
ACLr (4 wk) vs. ACLr (8 wk)	0.04382	-0.2689 to 0.3566	No	ns	0.9787	C-D
Test details	Mean 1	Mean 2	Mean Diff.	SE of diff.	n1	n2
Sham (4 wk) vs. Sham (8 wk)	0.7308	1.047	-0.3162	0.1167	5	6

Sham (4 wk) vs. ACLr (4 wk)	0.7308	0.6710	0.05987	0.1167	5	6
Sham (4 wk) vs. ACLr (8 wk)	0.7308	0.6272	0.1037	0.1167	5	6
Sham (8 wk) vs. ACLr (4 wk)	1.047	0.6710	0.3761	0.1112	6	6
Sham (8 wk) vs. ACLr (8 wk)	1.047	0.6272	0.4199	0.1112	6	6
ACLr (4 wk) vs. ACLr (8 wk)	0.6710	0.6272	0.04382	0.1112	6	6

Tibia SBP.TMD

Tukey's multiple comparisons test	Mean Diff.	95.00% CI of diff.	Below threshold?	Summary	Adjusted P Value	
Sham (4 wk) vs. Sham (8 wk)	-4.518	-20.91 to 11.87	No	ns	0.8648	A-B
Sham (4 wk) vs. ACLr (4 wk)	14.00	-2.394 to 30.39	No	ns	0.1108	A-C
Sham (4 wk) vs. ACLr (8 wk)	32.71	16.32 to 49.10	Yes	***	0.0001	A-D
Sham (8 wk) vs. ACLr (4 wk)	18.51	2.886 to 34.14	Yes	*	0.0169	B-C
Sham (8 wk) vs. ACLr (8 wk)	37.22	21.60 to 52.85	Yes	****	<0.0001	B-D
ACLr (4 wk) vs. ACLr (8 wk)	18.71	3.082 to 34.34	Yes	*	0.0157	C-D
Test details	Mean 1	Mean 2	Mean Diff.	SE of diff.	n1	n2
Sham (4 wk) vs. Sham (8 wk)	618.4	622.9	-4.518	5.829	5	6
Sham (4 wk) vs. ACLr (4 wk)	618.4	604.4	14.00	5.829	5	6
Sham (4 wk) vs. ACLr (8 wk)	618.4	585.7	32.71	5.829	5	6
Sham (8 wk) vs. ACLr (4 wk)	622.9	604.4	18.51	5.558	6	6
Sham (8 wk) vs. ACLr (8 wk)	622.9	585.7	37.22	5.558	6	6
ACLr (4 wk) vs. ACLr (8 wk)	604.4	585.7	18.71	5.558	6	6

Posterior Horn Bone Volume

Tukey's multiple comparisons test	Mean Diff.	95.00% CI of diff.	Below threshold?	Summary	Adjusted P Value	
Sham (4 wk) vs. Sham (8 wk)	-0.5646	-0.8958 to -0.2335	Yes	***	0.0008	A-B
Sham (4 wk) vs. ACLr (4 wk)	-0.3461	-0.6920 to -0.0002077	Yes	*	0.0498	A-C
Sham (4 wk) vs. ACLr (8 wk)	-0.09746	-0.4434 to 0.2484	No	ns	0.8530	A-D
Sham (8 wk) vs. ACLr (4 wk)	0.2185	-0.1126 to 0.5497	No	ns	0.2746	B-C
Sham (8 wk) vs. ACLr (8 wk)	0.4672	0.1360 to 0.7983	Yes	**	0.0046	B-D
ACLr (4 wk) vs. ACLr (8 wk)	0.2486	-0.09725 to 0.5945	No	ns	0.2114	C-D
Test details	Mean 1	Mean 2	Mean Diff.	SE of diff.	n1	n2
Sham (4 wk) vs. Sham (8 wk)	1.575	2.140	-0.5646	0.1165	5	6
Sham (4 wk) vs. ACLr (4 wk)	1.575	1.921	-0.3461	0.1217	5	5
Sham (4 wk) vs. ACLr (8 wk)	1.575	1.673	-0.09746	0.1217	5	5
Sham (8 wk) vs. ACLr (4 wk)	2.140	1.921	0.2185	0.1165	6	5
Sham (8 wk) vs. ACLr (8 wk)	2.140	1.673	0.4672	0.1165	6	5
ACLr (4 wk) vs. ACLr (8 wk)	1.921	1.673	0.2486	0.1217	5	5

Medial Posterior Horn Bone Density

Tukey's multiple comparisons test	Mean Diff.	95.00% CI of diff.	Below threshold?	Summary	Adjusted P Value	
Sham (4 wk) vs. Sham (8 wk)	-9.559	-23.21 to 4.090	No	ns	0.2321	A-B
Sham (4 wk) vs. ACLr (4 wk)	1.321	-12.93 to 15.58	No	ns	0.9935	A-C
Sham (4 wk) vs. ACLr (8 wk)	5.479	-8.170 to 19.13	No	ns	0.6737	A-D
Sham (8 wk) vs. ACLr (4 wk)	10.88	-2.768 to 24.53	No	ns	0.1468	B-C
Sham (8 wk) vs. ACLr (8 wk)	15.04	2.024 to 28.05	Yes	*	0.0204	B-D
ACLr (4 wk) vs. ACLr (8 wk)	4.157	-9.491 to 17.81	No	ns	0.8246	C-D
Test details	Mean 1	Mean 2	Mean Diff.	SE of diff.	n1	n2
Sham (4 wk) vs. Sham (8 wk)	593.7	603.2	-9.559	4.829	5	6
Sham (4 wk) vs. ACLr (4 wk)	593.7	592.4	1.321	5.044	5	5
Sham (4 wk) vs. ACLr (8 wk)	593.7	588.2	5.479	4.829	5	6
Sham (8 wk) vs. ACLr (4 wk)	603.2	592.4	10.88	4.829	6	5
Sham (8 wk) vs. ACLr (8 wk)	603.2	588.2	15.04	4.604	6	6
ACLr (4 wk) vs. ACLr (8 wk)	592.4	588.2	4.157	4.829	5	6

Femur SBP.Th

Tukey's multiple comparisons test	Mean Diff.	95.00% CI of diff.	Below threshold?	Summary	Adjusted P Value	
Sham (4 wk) vs. Sham (8 wk)	-0.008200	-0.03467 to 0.01827	No	ns	0.8148	A-B
Sham (4 wk) vs. ACLr (4 wk)	0.03095	0.005606 to 0.05629	Yes	*	0.0140	A-C
Sham (4 wk) vs. ACLr (8 wk)	-0.004580	-0.03105 to 0.02189	No	ns	0.9598	A-D
Sham (8 wk) vs. ACLr (4 wk)	0.03915	0.01381 to 0.06449	Yes	**	0.0020	B-C
Sham (8 wk) vs. ACLr (8 wk)	0.003620	-0.02285 to 0.03009	No	ns	0.9794	B-D
ACLr (4 wk) vs. ACLr (8 wk)	-0.03553	-0.06087 to -0.01019	Yes	**	0.0048	C-D
Test details	Mean 1	Mean 2	Mean Diff.	SE of diff.	n1	n2
Sham (4 wk) vs. Sham (8 wk)	0.2189	0.2271	-0.008200	0.009311	5	5
Sham (4 wk) vs. ACLr (4 wk)	0.2189	0.1879	0.03095	0.008915	5	6
Sham (4 wk) vs. ACLr (8 wk)	0.2189	0.2235	-0.004580	0.009311	5	5
Sham (8 wk) vs. ACLr (4 wk)	0.2271	0.1879	0.03915	0.008915	5	6
Sham (8 wk) vs. ACLr (8 wk)	0.2271	0.2235	0.003620	0.009311	5	5
ACLr (4 wk) vs. ACLr (8 wk)	0.1879	0.2235	-0.03553	0.008915	6	5

Femur SBP.BV

Tukey's multiple comparisons test	Mean Diff.	95.00% CI of diff.	Below threshold?	Summary	Adjusted P Value	
Sham (4 wk) vs. Sham (8 wk)	-0.4416	-0.8962 to 0.01298	No	ns	0.0585	A-B
Sham (4 wk) vs. ACLr (4 wk)	0.1791	-0.2562 to 0.6143	No	ns	0.6533	A-C
Sham (4 wk) vs. ACLr (8 wk)	-0.5506	-1.005 to -0.09602	Yes	*	0.0149	A-D
Sham (8 wk) vs. ACLr (4 wk)	0.6207	0.1854 to 1.056	Yes	**	0.0042	B-C

Sham (8 wk) vs. ACLr (8 wk)	-0.1090	-0.5636 to 0.3456	No	ns	0.9027	B-D
ACLr (4 wk) vs. ACLr (8 wk)	-0.7297	-1.165 to -0.2944	Yes	***	0.0009	C-D
Test details	Mean 1	Mean 2	Mean Diff.	SE of diff.	n1	n2
Sham (4 wk) vs. Sham (8 wk)	1.857	2.299	-0.4416	0.1599	5	5
Sham (4 wk) vs. ACLr (4 wk)	1.857	1.678	0.1791	0.1531	5	6
Sham (4 wk) vs. ACLr (8 wk)	1.857	2.408	-0.5506	0.1599	5	5
Sham (8 wk) vs. ACLr (4 wk)	2.299	1.678	0.6207	0.1531	5	6
Sham (8 wk) vs. ACLr (8 wk)	2.299	2.408	-0.1090	0.1599	5	5
ACLr (4 wk) vs. ACLr (8 wk)	1.678	2.408	-0.7297	0.1531	6	5

Femur SBP.TMD

Tukey's multiple comparisons test	Mean Diff.	95.00% CI of diff.	Below threshold?	Summary	Adjusted P Value	
Sham (4 wk) vs. Sham (8 wk)	-21.71	-29.84 to -13.58	Yes	****	<0.0001	A-B
Sham (4 wk) vs. ACLr (4 wk)	-7.190	-14.97 to 0.5912	No	ns	0.0757	A-C
Sham (4 wk) vs. ACLr (8 wk)	-11.87	-20.00 to -3.745	Yes	**	0.0034	A-D
Sham (8 wk) vs. ACLr (4 wk)	14.52	6.740 to 22.30	Yes	***	0.0003	B-C
Sham (8 wk) vs. ACLr (8 wk)	9.838	1.711 to 17.96	Yes	*	0.0149	B-D
ACLr (4 wk) vs. ACLr (8 wk)	-4.682	-12.46 to 3.099	No	ns	0.3488	C-D
Test details	Mean 1	Mean 2	Mean Diff.	SE of diff.	n1	n2
Sham (4 wk) vs. Sham (8 wk)	618.1	639.8	-21.71	2.859	5	5
Sham (4 wk) vs. ACLr (4 wk)	618.1	625.2	-7.190	2.737	5	6
Sham (4 wk) vs. ACLr (8 wk)	618.1	629.9	-11.87	2.859	5	5
Sham (8 wk) vs. ACLr (4 wk)	639.8	625.2	14.52	2.737	5	6
Sham (8 wk) vs. ACLr (8 wk)	639.8	629.9	9.838	2.859	5	5
ACLr (4 wk) vs. ACLr (8 wk)	625.2	629.9	-4.682	2.737	6	5

Patella Bone Volume

Tukey's multiple comparisons test	Mean Diff.	95.00% CI of diff.	Below threshold?	Summary	Adjusted P Value	
Sham vs. ACLR (4 wk)	1.141	-0.3285 to 2.610	No	ns	0.1395	A-B
Sham vs. ACLR (8 wk)	-1.107	-2.514 to 0.2999	No	ns	0.1333	A-C
ACLR (4 wk) vs. ACLR (8 wk)	-2.248	-3.655 to -0.8410	Yes	**	0.0027	B-C
Test details	Mean 1	Mean 2	Mean Diff.	SE of diff.	n1	n2
Sham vs. ACLR (4 wk)	11.46	10.32	1.141	0.5565	5	5
Sham vs. ACLR (8 wk)	11.46	12.56	-1.107	0.5328	5	6
ACLR (4 wk) vs. ACLR (8 wk)	10.32	12.56	-2.248	0.5328	5	6

Patella Bone Density

Tukey's multiple comparisons test	Mean Diff.	95.00% CI of diff.	Below threshold?	Summary	Adjusted P Value	
Sham vs. ACLR (4 wk)	41.40	29.70 to 53.10	Yes	****	<0.0001	A-B
Sham vs. ACLR (8 wk)	26.14	14.94 to 37.34	Yes	****	<0.0001	A-C
ACLR (4 wk) vs. ACLR (8 wk)	-15.26	-26.46 to -4.060	Yes	**	0.0085	B-C
Test details	Mean 1	Mean 2	Mean Diff.	SE of diff.	n1	n2
Sham vs. ACLR (4 wk)	632.9	591.5	41.40	4.431	5	5
Sham vs. ACLR (8 wk)	632.9	606.8	26.14	4.242	5	6
ACLR (4 wk) vs. ACLR (8 wk)	591.5	606.8	-15.26	4.242	5	6
