# Supplementary material for: Structural osteoarthritis pathogenesis correlates with distinct pain and dysfunction profiles after ACL injury in rats
Source: Lab Anim (NY). 2026 Jul 1;55(9):344–53. doi: 10.1038/s41684-026-01762-1 (PMC13518234; doi:10.1038/s41684-026-01762-1)
Supplement: Supplementary file 2 [file 41684_2026_1762_MOESM2_ESM.pdf]

Corresponding author(s): *Double-anonymous peer review submissions: write DAPR and your manuscript number here instead of author names.*

Last updated by author(s): Nov 14, 2025

Reporting Summary

Nature Portfolio wishes to improve the reproducibility of the work that we publish. This form provides structure for consistency and transparency in reporting. For further information on Nature Portfolio policies, see our Editorial Policies and the Editorial Policy Checklist.

Statistics

For all statistical analyses, confirm that the following items are present in the figure legend, table legend, main text, or Methods section.

n/a	Confirmed	
☐	☒	The exact sample size (*n*) for each experimental group/condition, given as a discrete number and unit of measurement
☐	☒	A statement on whether measurements were taken from distinct samples or whether the same sample was measured repeatedly
☐	☒	The statistical test(s) used AND whether they are one- or two-sided<br>*Only common tests should be described solely by name; describe more complex techniques in the Methods section.*
☐	☒	A description of all covariates tested
☐	☒	A description of any assumptions or corrections, such as tests of normality and adjustment for multiple comparisons
☐	☒	A full description of the statistical parameters including central tendency (e.g. means) or other basic estimates (e.g. regression coefficient) AND variation (e.g. standard deviation) or associated estimates of uncertainty (e.g. confidence intervals)
☐	☒	For null hypothesis testing, the test statistic (e.g. *F*, *t*, *r*) with confidence intervals, effect sizes, degrees of freedom and *P* value noted<br>*Give P values as exact values whenever suitable.*
☒	☐	For Bayesian analysis, information on the choice of priors and Markov chain Monte Carlo settings
☒	☐	For hierarchical and complex designs, identification of the appropriate level for tests and full reporting of outcomes
☐	☒	Estimates of effect sizes (e.g. Cohen's *d*, Pearson's *r*), indicating how they were calculated

Our web collection on statistics for biologists contains articles on many of the points above.

Software and code

Policy information about availability of computer code

Data collection	Bioseb DWB 2.0 and EvF systems (Bioseb, Park, FL, USA). Scanco VivaCT80 (Scanco Medical, Wangen-Brüttisellen, Switzerland)
Data analysis	GraphPad Prism 10.3.0 (GraphPad Software, Boston, MA, USA). (Metaboanalyst 6.0, Montreal, QC, Canada)

For manuscripts utilizing custom algorithms or software that are central to the research but not yet described in published literature, software must be made available to editors and reviewers. We strongly encourage code deposition in a community repository (e.g. GitHub). See the Nature Portfolio guidelines for submitting code & software for further information.

Data

Policy information about availability of data

All manuscripts must include a data availability statement. This statement should provide the following information, where applicable:

- Accession codes, unique identifiers, or web links for publicly available datasets
- A description of any restrictions on data availability
- For clinical datasets or third party data, please ensure that the statement adheres to our policy

Data are available per request via email with the corresponding PI

Research involving human participants, their data, or biological material

Policy information about studies with human participants or human data. See also policy information about sex, gender (identity/presentation), and sexual orientation and race, ethnicity and racism.

Reporting on sex and gender	Human participants were not used in this study
Reporting on race, ethnicity, or other socially relevant groupings	Human participants were not used in this study
Population characteristics	Human participants were not used in this study
Recruitment	Human participants were not used in this study
Ethics oversight	Human participants were not used in this study

Note that full information on the approval of the study protocol must also be provided in the manuscript.

Field-specific reporting

Please select the one below that is the best fit for your research. If you are not sure, read the appropriate sections before making your selection.

☒ Life sciences ☐ Behavioural & social sciences ☐ Ecological, evolutionary & environmental sciences

For a reference copy of the document with all sections, see nature.com/documents/nr-reporting-summary-flat.pdf

Life sciences study design

All studies must disclose on these points even when the disclosure is negative.

Sample size	An initial pilot study was conducted to due to a lack of a priori data, from which, a power analysis was conducted to estimate a projected sample size of n=10 for behavioral measures
Data exclusions	Data was excluded from behavioral testing only in the case of failed collection (e.g., failed computer memory with weight bearing data) and changes to sample sizes are noted in the captions. Structural data was excluded if damage was incurred during tissue isolation (e.g.,patella at 4-weeks in Sham groups), and is noted in captions.
Replication	Experiments were not reproduced due to the use of ethically and financially rat models. Validity of this injury modality was as initially conducted in a non-survival pilot study, which is described in supplemental content.
Randomization	Randomization was conducted using an Excel Randomization function using the Animal Identification numbers as inputs.
Blinding	Investigators were blinded to treatment group during collection of behavioral testing and computed tomography analysis. Details are described in Methods.

Reporting for specific materials, systems and methods

We require information from authors about some types of materials, experimental systems and methods used in many studies. Here, indicate whether each material, system or method listed is relevant to your study. If you are not sure if a list item applies to your research, read the appropriate section before selecting a response.

Materials & experimental systems

n/a	Involved in the study	
☒	☐	Antibodies
☒	☐	Eukaryotic cell lines
☒	☐	Palaeontology and archaeology
☐	☒	Animals and other organisms
☒	☐	Clinical data
☒	☐	Dual use research of concern
☒	☐	Plants

Methods

n/a	Involved in the study	
☒	☐	ChIP-seq
☒	☐	Flow cytometry
☒	☐	MRI-based neuroimaging

Animals and other research organisms

Policy information about studies involving animals; ARRIVE guidelines recommended for reporting animal research, and Sex and Gender in Research

Laboratory animals	Male Lewis
Wild animals	Wild animals were not used in this study
Reporting on sex	Male Lewis rats were selected for their propensity to rapidly develop a consistent osteoarthritis pathology due a heavy body weight. Lewis rats exhibit high sexual dimorphism, resulting in inconsistencies in joint disease progression when comparing between sex in this model, which is not reflective of human comparative disease.
Field-collected samples	Field-collected samples were not used in this study
Ethics oversight	University of Oregon IACUC provided oversight of this work

Note that full information on the approval of the study protocol must also be provided in the manuscript.

Plants

Seed stocks	Plants were not used in this study
Novel plant genotypes	Plants were not used in this study
Authentication	Plants were not used in this study
